# Network Pharmacology Identifies the Mechanisms of Sang-Xing-Zhi-Ke-Fang against Pharyngitis

**DOI:** 10.1155/2020/2421916

**Published:** 2020-10-12

**Authors:** Yinhe Deng, Quanjiang Li, Menglin Li, Tiantian Han, Guixian Li, Qiong Liu

**Affiliations:** ^1^College of First Clinical Medical, Guangzhou University of Chinese Medicine, Guangzhou 51000, China; ^2^International Medical Department, The First Affiliated Hospital of Guangzhou University of Chinese Medicine, Guangzhou 51000, China

## Abstract

**Background:**

Sang-Xing-Zhi-Ke-Fang (SXZKF) demonstrates good therapeutic effect against pharyngitis. Nevertheless, the pharmacological mechanism underlying its effectiveness is still unclear.

**Objective:**

To investigate the underlying mechanisms of SXZKF against pharyngitis using network pharmacology method.

**Methods:**

Bioactive ingredients of SXZKF were collected and screened using published literature and two public databases. Using four public databases, the overlapping genes between these bioactive compound-related and pharyngitis-related genes were identified by Venn diagram. Protein-protein interaction (PPI) was obtained using “Search Tool for the Retrieval of Interacting Genes (STRING)” database. “Database for Annotation, Visualization, and Integrated Discovery ver. 6.8 (DAVID 6.8)” was used to perform Kyoto Encyclopedia of Genes and Genomes (KEGG) pathway enrichment analysis to explore the molecular mechanisms of SXZKF against pharyngitis. Finally, Cytoscape 3.7.2 software was used to construct and visualize the networks.

**Result:**

A total of 102 bioactive compounds were identified. Among them, 886 compounds-related and 6258 pharyngitis-related genes were identified, including 387 overlapping genes. Sixty-three core targets were obtained, including ALB, PPAR*γ*, MAPK3, EGF, and PTGS2. Signaling pathways closely related to mechanisms of SXZKF for pharyngitis were identified, including serotonergic synapse, VEGF signaling pathway, Fc epsilon RI signaling pathway, Ras signaling pathway, MAPK signaling pathway, and influenza A.

**Conclusion:**

This is the first identification of in-depth study of SXZKF against pharyngitis using network pharmacology. This new evidence could be informative in providing new support on the clinical effects of SXZKF on pharyngitis and for the development of personalized medicine for pharyngitis.

## 1. Introduction

Pharyngitis, a collective term for inflammation caused by various microorganisms in the pharynx, still has a high incidence even with the rapid development of modern medicine [1]. In China, acute pharyngitis is mainly treated with antiviral drugs, antibiotics or traditional Chinese medicine (TCM) [2, 3]. TCM plays an increasingly important role in the treatment of chronic pharyngitis, for the etiology of chronic pharyngitis is complex, and many studies have shown that TCM shows significant effect on this disease [4, 5].

Sang-Xing-Zhi-Ke-Fang is a concoction based on Sang-Xing-Tang, which is originated form Wen Bing Tiao BiannBia that had been widely used for more than 200 years in China. SXZKF has been patented in China (patent number: 201910302604.4) in 2019 and the oral lozenge based on it is being developed. SXZKF is composed of Mulberry Leaf, Armeniacae Amarum Semen, Radix Glehniae, Radix Rehmanniae Praeparata, Loguat Leaf, Fritillary Bulb, Eriobotrya japonica Thunb, Ligusticum chuanxiong hort, Earthworm Lumbricus, Mentha haplocalyx Briq, and Exocarpium Citrus Grandis. Although we have previously verified that SXZKF was effective against pharyngitis [6], the active ingredients and molecular mechanisms of SXZKF in the treatment of pharyngitis remains unknown.

Network pharmacology is a new discipline based on the theory of systems biology, analyzing the network of biological systems, and selecting specific signal nodes for multitarget drug molecular design [7]. Based on the ideas and methodologies of network pharmacology, this study aimed to obtain the main active ingredients of SXZKF, screened out the core targets and main biological pathways, and explored the molecular mechanisms of SXZKF in the treatment of pharyngitis.

## 2. Material and Methods

### 2.1. Screening of Bioactive Compounds of SXZKF

Information of the bioactive compounds in SXZKF was obtained from Traditional Chinese Medicine Systems Pharmacology Database and Analysis Platform (TCMSP, http://lsp.nwu.edu.cn/tcmsp.php) and Bioinformatics Analysis Tool for Molecular mechanism of Traditional Chinese Medicine (BATMAN-TCM, http://bionet.ncpsb.org/batman-tcm/) [8]. The active ingredients of SXZKF were filtered using the following criteria: (i) oral bioavailability (OB) ≥ 30% and (ii) drug-likeness (DL) ≥ 0.18 [9, 10]. Other ingredients were obtained through literature analysis [11, 12].

### 2.2. Screening of Target Genes of Bioactive Compounds and Pharyngitis

Target genes of bioactive compounds of SXZKF were obtained from TCMSP, and the Universal Protein Database (UniProt, https://www.uniprot.org/) was then used to remove nonhuman target gene names. To ensure the reliability of prediction, only target genes with “Reviewed/Swiss-Prot” rots “Human” in UniProt would be selected [13, 14]. The targets related to pharyngitis were obtained from Comparative Toxicogenomics Database (CTD, http://ctdbase.org/) [15] and The Human Gene Database (GeneCards, http://www.genecards.org/). By searching the keyword “pharyngitis,” target genes of pharyngitis were identified.

### 2.3. Establishment of Herbs-Compounds-Targets (H-C-T) Network

The overlapping target genes relating to both the bioactive compounds and pharyngitis were identified and visualized by Venn diagrams (http://bioinformatics.psb.ugent.be/webtools/Venn/). Then, Cytoscape 3.7.2 software was used to visualize and analyze the network of interactions between overlapping genes, bioactive compounds, and herbs; we named it the H-C-T network.

### 2.4. Analysis of Network Topological Features

STRING database (https://string-db.org/) was used to obtain the protein-protein interactions (PPI) by uploading 387 overlapping targets between active compounds and pharyngitis. Species was limited to “*Homo sapiens*” with a confidence score >0.4. Cytoscape 3.7.2 was used to construct the network of PPI, and then the topological features of the network were analyzed to screen out the core targets that play a crucial part in the PPI network. The degree centrality (DC), betweenness centrality (BC), and closeness centrality (CC) were analyzed for each node using the plugin cytoNCA in Cytoscape 3.7.2 [16]. Preliminary screening was carried out for nodes with a DC equal or larger than the median of two. Finally, the nodes with BC and CC, both larger than the median of two, were identified as the core targets [17].

### 2.5. Pathway Analyses and Construction of Compounds-Targets-Pathways (C-T-P) Network

**Figure 1:**
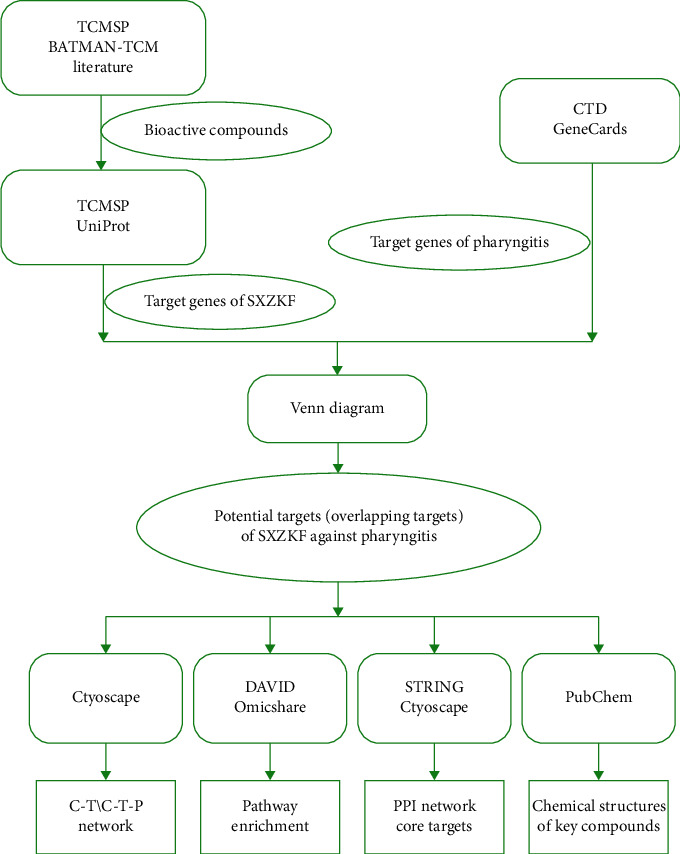
Workflow of this study.

Pathway enrichment analysis for overlapping genes was performed using DAVID ver. 6.8 (https://david.ncifcrf.gov/) with the “*Homo sapiens*” setting. Generally, the results of KEGG pathway enrichment were considered to have statistically significant and necessary functional mechanisms of pharyngitis when *P* < 0.05. To ensure the accuracy of this study, we set the screening criteria as *P* < 0.01. Bubble chart of the concerned KEGG pathways was plotted using the OmicShare tools (https://www.omicshare.com/tools). Finally, Cytoscape 3.7.2 was used to establish and visualize the network of interactions between compounds, targets, and signaling pathways that are closely related to pharyngitis. The complete workflow of the analysis is given in Figure 1.

## 3. Results

### 3.1. Bioactive Compounds of SXZKF

Ninety-three bioactive compounds were identified from TCMSP and BATMAN-TCM, while another nine bioactive compounds were identified through literature survey. Among these, 29 compounds were identified from Mulberry Leaf, 19 compounds from Semen *Armeniacae Amarum Semen*, 8 compounds from *Radix Glehniae*, 2 compounds from *Radix Rehmanniae Praeparata*, 18 compounds from Loguat Leaf, 10 compounds from *Mentha haplocalyx Briq*, 7 compounds from *Fritillary Bulb*, 7 compounds from *Ligusticum chuanxiong hort,* 10 compounds from *Exocarpium Citrus Grandis*, and 9 compounds from Earthworm Lumbricus (Table S1).

### 3.2. Target Genes of SXZKF and Pharyngitis

**Figure 2:**
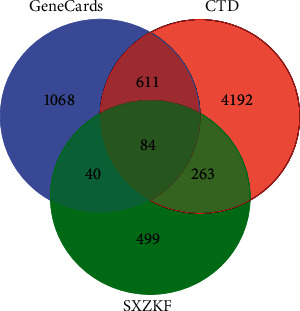
387 overlapping genes between SXZKF compounds-related targets and pharyngitis-related targets from GeneCards and CTD.

A total of 886 targets genes (Table S2) of 63 bioactive compounds were retrieved from TCMSP and screened by UniProt, and no genes were related to another 39 compounds. On the other hand, 6258 targets related to pharyngitis were obtained (5150 targets from CTD, 1803 targets from GeneCards; among them 695 duplicate targets), and the information is listed in Table S3. As shown in Figure 2, the Venn diagram showed that 387 overlapping target genes were identified by matching the SXZKF compounds-related target genes with CTD and GeneCards pharyngitis-related target genes.

### 3.3. Analysis of H-C-T Network

**Figure 3:**
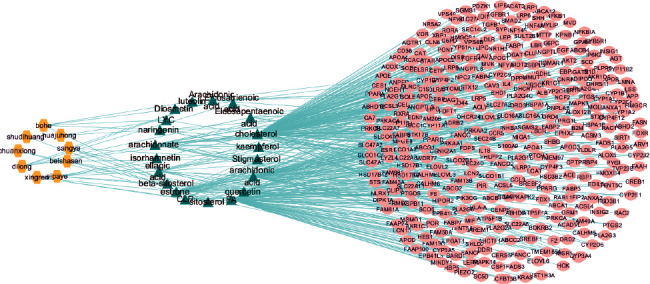
H-C-T network. Yellow nodes represent the herbs of SXZKF, green nodes represent the bioactive compounds of SXZKF, and pink nodes represent the potential targets of SXZKF against pharyngitis.

Based on the Venn diagram and the results of TCMSP retrieval, we can obtain that the 387 potential targets were linked to 19 compounds of SXZKF against pharyngitis, as shown in Table S4. To get a better standing of the interactions between herbs, compounds, and target genes, a visual network with 415 nodes (including 9 herbs, 19 compounds, and 387 targets) and 546 edges was established by Cytoscape as shown in Figure3.

### 3.4. PPI Network and Core Targets

**Figure 4:**
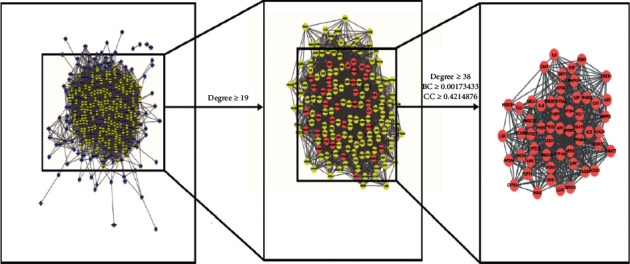
Screening of core targets by analyzing topological features of the PPI network. Red nodes represent the core targets.

We used the plugin cytoNCA in the Cytoscape 3.7.2 to calculate the degree centrality (DC), betweenness centrality (BC) and closeness centrality (CC) for each node in PPI network, the results are shown in Table S5. According to these values, the targets with DC, BC, and CC larger than the median of two were used as core targets for subsequent analysis (as shown in Figure 4). As displayed in Table 1, 63 core targets were finally obtained.

### 3.5. Pathway Enrichment Analysis

**Figure 5:**
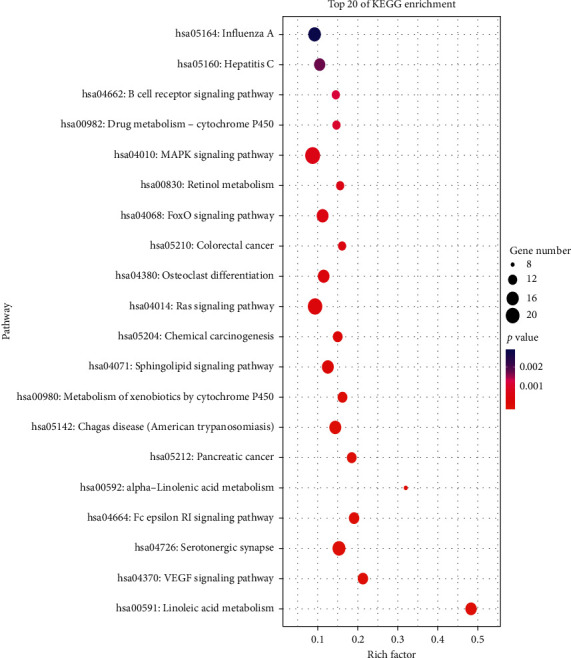
Bubble chart of top 20 signaling pathways linked to SXZKF against pharyngitis. Bubble size represented the number of genes enriched in this pathway, color depth represented the *P* value, and rich factor represented the ratio of the enriched targets in the pathway to the total number of targets in the pathway.

387 potential targets were mapped to a total of 109 signaling pathways using DAVID, 28 of which were identified as *P* < 0.01 (as shown in Table S6). Top 20 KEGG pathways' enrichment analysis is shown in Figure 5 and Table 2.

### 3.6. Analysis of C-T-P Network

**Figure 6:**
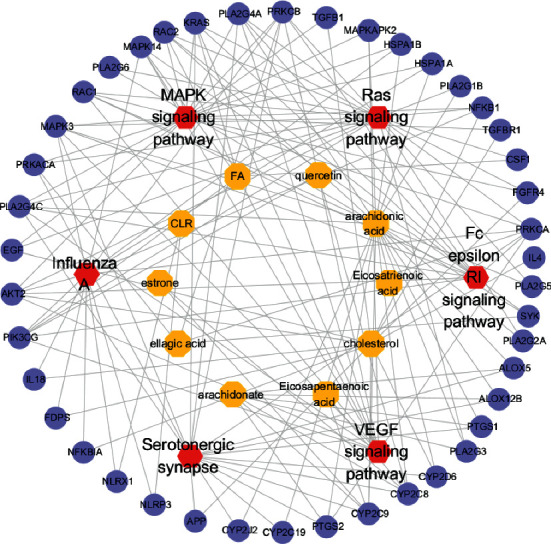
C-T-P network. Yellow nodes represent the bioactive compounds, red nodes represent the key pathways, and purple nodes represent the target genes.

After reviewing the published literature, we narrowed down to 6 of the top 20 signaling pathways, which are closely related to pharyngitis. These includes serotonergic synapse, VEGF signaling pathway, Fc epsilon RI signaling pathway, Ras signaling pathway, MAPK signaling pathway, and influenza A. Cytoscape 3.7.2 was used to construct the network of “compounds-key pathways-targets,” consisting of 59 nodes (including 10 compounds, 6 pathways, and 43 targets) and 158 edges, as demonstrated in Figure 6.

## 4. Discussion

As seen in Figures 3 and 6, SXZKF has effects on multiple components, multiple targets, and multiple pathways, which also indicates that TCM plays a synergistic role in the treatment of diseases. Many studies have shown that acute pharyngitis is caused by viral or bacterial infection, and the overlapping pathogenic bacteria include group A beta-hemolytic streptococcus (the most overlapping) as well as groups C and G streptococcus. In addition, influenza A virus, influenza B virus, rhinovirus, and respiratory syncytial virus are important factors causing pharyngitis [18]. Although the etiology of chronic pharyngitis is complex, bacterial infection is now affirmed as the most important cause, followed by noninfectious factors, such as obstructive sleep apnea hypopnea syndrome, occupational exposure, laryngeal reflux, and allergic diseases. Noninfectious factors combined with microbial infection can induce resistance in the disease progression [19]. Meanwhile, many research have shown that a variety of cytokines are related to the occurrence and development of both acute and chronic pharyngitis. These cytokines include inflammatory factor, tumor necrosis factor (TNF), and arachidonic acid and its cyclooxygenase metabolites, serotonin (5-HT, also known as serotonin), interleukins (IL-1, IL-2, IL-4, IL-6, IL-10, etc.), epidermal growth factor (EGF), and so on [20].

**Figure 7:**
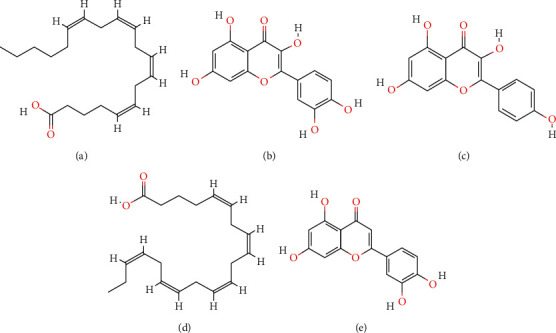
Chemical structures of key compounds. (a) Arachidonic acid. (b) Quercetin. (c) Kaempferol. (d) Eicosapentaenoic acid. (e) Luteolin.

In our current study, a total of 102 bioactive ingredients were identified in SXZKF. The main active ingredients are arachidonic acid, quercetin, kaempferol, eicosapentaenoic acid, and luteolin, the compounds with higher degree value of node in the H-C-T and C-T-P network, whose chemical structures were shown in Figure 7. Among, the main active ingredients identified, metabolites of arachidonic acid (AA) contribute to inflammation as well as to resolving inflammation. Zhang et al. study demonstrated that AA can directly bind to TLR4 coreceptor, myeloid differentiation factor 2 (MD2), and prevent saturated fatty acids from activating TLR4 proinflammatory signaling pathway. AA can also reduce lipopolysaccharide- (LPS-) induced inflammation in macrophages and septic death in mice through binding to MD2 [21]. Eicosapentaenoic acid (EPA) plays an anti-inflammatory role in the body, which can interfere with the PPAR*α*-I*κ*B-NF-*κ*B signaling pathway in inflammatory cells to inhibit the inflammatory response due to the strong anti-inflammatory effects of resolvin and protectin [22]. The proinflammatory and anti-inflammatory effects of AA and EPA intercoordinate to prevent excessive inflammatory response and chronic low-grade inflammation [23]. Besides, kaempferol, luteolin, and quercetin can reduce the production of the proinflammatory factor IL-6 or tumor necrosis factor (TNF) to promote the production of the anti-inflammatory factor IL-10 or reduce the expression of Cox-2 and inducible nitric oxide synthase (iNOS) to downregulate the level of NO and PGE2 [24]. Kim et al. showed that quercetin and kaempferol can significantly reduce mice's ear swelling, relieves writhing response, and can significantly prevent cough in mice, indicating anti-inflammatory, analgesic, and antitussive activities of these compounds [25]. In terms of antiviral effects, Yan et al. showed that luteolin has a certain inhibitory effect on influenza A virus (H1N1), as it may reduce the mRNA expression of hemagglutinin (HA) and neuraminidase (NA) in virus-infected cells [26]. In terms of antibacterial effects, extracts of luteolin and kaempferol have the highest activity against Gram-positive bacteria *in vitro*, especially against *Streptococcus aureus*, *Streptococcus pneumoniae, Streptococcus epidermidis, Bacillus cereus*, and *Bacillus subtilis* [27].

A total of 63 key targets for SXZKF in the treatment of pharyngitis were identified. DC, CC, and BC analysis indicated that ALB (serum albumin), PPAR*γ* (peroxisome proliferator-activated receptor gamma), MAPK3 (mitogen-activated protein kinase 3), EGF (epidermal growth factor), and PTGS2 (prostaglandin G/H synthase 2) are closely related to pharyngitis. ALB is an important substance to maintain plasma colloid osmotic pressure, but bacterial infection in the throat can cause local vascular permeability to increase, lead to vasodilatation and exudation of the serous fluid, and finally result in hyperemia, pain, and even thickening of the mucosa. When localized albumin synthesis increases when the throat is inflamed, ALB acts to maintain intravascular osmotic pressure, reduce serous exudation, and alleviate the symptoms as well as processes of pharyngeal inflammation [28]. EGF promotes the growth of various epidermal cells and play an important role as a mucosal protector in mucosal defense and ulcer healing [29]. Studies have shown that EGF is related to the healing of chronic pharyngeal inflammation and participates in the pharyngeal mucosa repair [30]. PPAR*γ* is an essential transcription factor, which can act as an inhibitor for inflammatory gene expression, blocking the reverses inflammation [31, 32]. MAPK can transmit signals from receptors on the cell surface to DNA in the nucleus and participate in biological processes, such as cell growth, death, and cell cycle, and also in the regulation of pathological processes, such as inflammation and stress response. As a member of the MAPK family, MAPK3 plays an important role in the process of proliferation, differentiation, and formation of inflammatory cells [33, 34]. PTGS2 is an inducible immediate response gene that is not expressed in most cells under normal physiological conditions, but during pathological reactions such as inflammation or tumors, the expression of PTGS2 is rapidly upregulated, producing a large amount of prostaglandins. Prostaglandins are mediators of inflammation that expand blood vessels and increase the sensitivity of nerve endings to bradykinin and histamine, leading to inflammatory pain [35].

This study analyzes the six pathways that are highly relevant to the pathogenesis of pharyngitis. Vasodilation and serous exudation are important links in the pathological process of pharyngitis. Vascular endothelial growth factor (VEGF) is the most effective angiogenesis factor in the body, which can bind to specific receptors of vascular endothelial cells, promote the division and proliferation of vascular endothelial cells, and promote the generation of new blood vessels and increase vascular permeability [36]. VEGF-mediated signaling pathways can regulate the proliferation, migration, and survival of vascular endothelium cell, which cause changes in vascular permeability, leading to vasodilation, exudation, and inflammation. Study had shown that serum VEGF in pharyngitis model rats is higher than normal rats and decreases after treatment with TCM [37]. Serotonin, also known as 5-HT, is a nerve-conducting substance. Studies have demonstrated that 5-HT can increase the permeability of submucosal blood vessels, promote plasma extravasation, and increase congestion and edema, causing sore throat [20]. Serotonergic synaptic pathways can regulate the binding of serotonin to mediated receptors and alleviate pain symptoms. The etiology of some chronic pharyngitis is related to allergic reactions, and subjective symptoms of patients include pharyngeal foreign body sensation, itchy throat, pharynx swelling, and dry cough symptoms [38]. The combination of immunoglobulin E(IgE) with Fc*ε*RI and Fc epsilon RI signal transduction pathway are the keys in causing allergic diseases [39]. The Fc epsilon RI signal transduction pathway can regulate the process of allergy-associated pharyngitis. The Ras signaling pathway is mainly composed of signal pathways such as MAPK and PI3K-AKt (phosphatidylinositol-3-kinase-protein kinase B), which can regulate and direct the differentiation of CD4 + T lymphocytes, thereby reducing the inflammatory response. The MAPK pathway plays an important role in regulating the inflammatory response, and its function mainly includes three cascades mediated by ERK1/2, JNK, and p38 MAPK. Inactivating this pathway can reduce inflammatory cytokine production and relieve the inflammatory response [40]. Last but not least, as previously explained, influenza virus is an important factor in causing pharyngitis, while SXZKF can play an antiviral role in the treatment of pharyngitis by regulating the influenza A signaling pathway.

## 5. Conclusion

In conclusion, this study identified the main bioactive ingredients, core target genes and molecular mechanisms of SXZKF in the treatment of pharyngitis through network pharmacology. A total of 102 bioactive compounds were found, with arachidonic acid, quercetin, kaempferol, eicosapentaenoic acid, and luteolin identified as main active compounds. Among them, 63 key target genes were identified, with ALB, PPAR*γ*, MAPK3, EGF, and PTGS2 recognized as core targets. The main molecular mechanisms of SXZKF for pharyngitis consisted of 28 signaling pathways, and the key pathways that were closely related to pharyngitis were found to be related to analgesia, inhibition of inflammation response, inhibition of viral replication, and inhibition of anaphylactic reaction through inactivating serotonergic synaptic, VEGF signaling pathway, Fc epsilon RI signaling pathway, Ras signaling pathway, MAPK signaling pathway, and influenza A. Taken together, this study provides an insight on the cellular and pathway mechanism of SXZKF in the treatment of pharyngitis. This new technique could also be used to understand many other traditional or alternative medicine to provide a new horizon for the development of personalized TCM in the near future.

## Figures and Tables

**Table 1:** Detailed information of 63 core targets.

NO.	Target name	Degree centrality	Betweenness centrality	Closeness centrality
1	ALB	132	0.10194051	0.57963875
2	SREBF1	101	0.04648343	0.54058193
3	APOB	88	0.02798912	0.52373887
4	APOE	87	0.03066411	0.52765321
5	PPAR*γ*	84	0.02496889	0.53082707
6	MAPK3	82	0.03803816	0.52923538
7	HMGCR	79	0.02839721	0.51911765
8	EGF	77	0.0326961	0.51683748
9	LDLR	74	0.01786479	0.5
10	FASN	74	0.01620772	0.502849
11	PPARA	74	0.01758367	0.5130814
12	PTGS2	73	0.03636725	0.50573066
13	SREBF2	72	0.02642683	0.4862259
14	APP	68	0.02026671	0.50500715
15	LEP	67	0.01366234	0.50864553
16	ABCA1	66	0.02050839	0.49509116
17	SCD	66	0.01940855	0.48891967
18	APOA1	65	0.0104694	0.48891967
19	CAT	59	0.0231604	0.50070922
20	ADIPOQ	59	0.01236963	0.49788434
21	CYP3A4	59	0.01670894	0.48356164
22	ESR1	58	0.0240653	0.49509116
23	DGAT1	54	0.00895186	0.47446237
24	CYP2B6	53	0.01261496	0.48422497
25	APOC3	51	0.00521994	0.45844156
26	ACACA	51	0.0040295	0.47638327
27	CREB1	51	0.01442992	0.49164345
28	SIRT1	50	0.01736748	0.49301676
29	MAPK14	48	0.00877424	0.47702703
30	FABP1	48	0.01145724	0.46693122
31	CAV1	48	0.01458166	0.48092643
32	SQLE	47	0.00778386	0.44853875
33	AGT	47	0.00760543	0.47510094
34	CLU	47	0.00773464	0.45844156
35	LIPE	47	0.00794991	0.47638327
36	FADS1	47	0.00692009	0.45607235
37	LIPC	46	0.00999712	0.44968153
38	CYP2E1	46	0.01342474	0.4738255
39	IL4	45	0.00870765	0.47192513
40	ACOX1	45	0.0035187	0.45784695
41	HMGCS1	44	0.00325402	0.44853875
42	ACLY	44	0.01071917	0.46083551
43	CYP19A1	44	0.01030837	0.47255689
44	CYP2C9	44	0.00700349	0.46754967
45	SCARB1	44	0.00915057	0.47003995
46	FDFT1	43	0.00425437	0.45140665
47	DGAT2	43	0.00166345	0.44291092
48	APOA4	43	0.00578781	0.45314506
49	CD44	43	0.01055526	0.46816976
50	CYP51A1	41	0.00425467	0.44180225
51	HSD3B1	41	0.00690686	0.44180225
52	DHCR7	41	0.00378824	0.42891859
53	PON1	41	0.00478681	0.46693122
54	LSS	40	0.00646986	0.44570707
55	ACE	40	0.00407366	0.47255689
56	ABCG2	40	0.02206365	0.46816976
57	F2	39	0.00736964	0.47319035
58	KRAS	39	0.00749875	0.46143791
59	LCAT	39	0.00395196	0.46204188
60	NR3C1	39	0.00692536	0.47003995
61	FABP4	38	0.0045635	0.44796954
62	ABCB1	38	0.01106088	0.46693122
63	HNF4A	38	0.01122196	0.47574124

**Table 2:** Functions of 387 target genes based on KEGG pathway analysis.

Term	Number of pathway gene	*P* value
Linoleic acid metabolism	CYP3A4, CYP2J2, CYP2C19, CYP2C9, CYP2C8, CYP2E1, CYP1A2, PLA2G4A, PLA2G1B, PLA2G2A, PLA2G6, PLA2G3, PLA2G4C, PLA2G5	1.20*E* − 11
VEGF signaling pathway	PIK3CG, PRKCA, PTGS2, MAPKAPK2, PRKCB, PLA2G4A, KRAS, RAC2, MAPK14, MAPK3, RAC1, PLA2G4C, AKT2	2.69*E* − 06
Serotonergic synapse	PRKCA, PLA2G4A, APP, CYP2J2, KRAS, CYP2C19, PTGS2, CYP2C9, CYP2C8, CYP2D6, MAPK3, PTGS1, ALOX12B, PRKACA, ALOX5, PLA2G4C, PRKCB	4.54*E* − 06
Fc epsilon RI signaling pathway	IL4, PIK3CG, PRKCA, PRKCB, PLA2G4A, KRAS, RAC2, MAPK14, MAPK3, RAC1, PLA2G4C, SYK, AKT2	8.83*E* − 06
Alpha-linolenic acid metabolism	ACOX1, PLA2G4A, PLA2G2A, PLA2G1B, PLA2G6, PLA2G4C, PLA2G3, PLA2G5	3.08*E* − 05
Pancreatic cancer	PIK3CG, KRAS, RAC2, TGFBR1, MAPK3, TGFBR2, RAC1, NFKB1, SMAD2, EGF, TGFB1, AKT2	3.17*E* − 05
Chagas disease (American trypanosomiasis)	PIK3CG, CCL3, TGFBR1, TGFBR2, NFKBIA, NFKB1, SMAD2, BDKRB2, CALR, TGFB1, ACE, MAPK14, MAPK3, FAS, AKT2	3.98*E* − 05
Metabolism of xenobiotics by cytochrome P450	CYP3A4, CYP2A13, CYP3A5, CYP1B1, CYP1A1, CYP2B6, CYP2C9, CYP2D6, CYP2A6, CYP2E1, CYP1A2, AKR1C1	1.09*E* − 04
Sphingolipid signaling pathway	PRKCA, PIK3CG, SGMS2, CERS5, NFKB1, SGMS1, BDKRB2, PRKCB, KRAS, RAC2, MAPK14, MAPK3, RAC1, ABCC1, AKT2	1.93*E* − 04
Chemical carcinogenesis	CYP3A4, CYP2A13, CYP3A5, CYP1B1, CYP1A1, CYP2C19, PTGS2, CYP2C9, CYP2C8, CYP2A6, CYP2E1, CYP1A2	2.22*E* − 04
Ras signaling pathway	PRKCA, PIK3CG, FGFR4, CSF1, NFKB1, PRKCB, PLA2G4A, KRAS, RAC2, RAC1, MAPK3, PLA2G1B, PLA2G2A, PLA2G6, PRKACA, FGF1, PLA2G3, PLA2G4C, EGF, PLA2G5, AKT2	4.17*E* − 04
Osteoclast differentiation	PIK3CG, NCF2, TGFBR1, CSF1, CREB1, PPARG (PPAR*γ*P), TGFBR2, NFKBIA, NFKB1, TGFB1, MAPK14, MAPK3, RAC1, AKT2, SYK	4.83*E* − 04
Colorectal cancer	PIK3CG, KRAS, RAC2, TGFBR1, MAPK3, TGFBR2, RAC1, SMAD2, TGFB1, AKT2	5.51*E* − 04
FoxO signaling pathway	PIK3CG, TGFBR1, TGFBR2, SMAD2, SIRT1, TGFB1, G6PC, KRAS, MAPK14, MAPK3, PRKAA1, CAT, PRKAA2, EGF, AKT2	6.08*E* − 04
Retinol metabolism	CYP3A4, CYP3A5, DGAT1, CYP1A1, CYP2B6, CYP2C9, CYP2C8, HSD17B6, CYP2A6, CYP1A2	7.00*E* − 04
MAPK signaling pathway	PRKCA, FGFR4, TGFBR1, TGFBR2, NFKB1, HSPA1A, HSPA1B, MAPKAPK2, TGFB1, PRKCB, PLA2G4A, KRAS, RAC2, MAPK14, RAC1, MAPK3, PRKACA, FAS, FGF1, PLA2G4C, EGF, AKT2	7.04*E* − 04
Drug metabolism-cytochrome P450	CYP3A4, CYP3A5, CYP2C19, CYP2B6, CYP2C9, CYP2C8, CYP2D6, CYP2A6, CYP2E1, CYP1A2	0.001095
B Cell receptor signaling pathway	PIK3CG, KRAS, RAC2, MAPK3, RAC1, CD81, NFKBIA, NFKB1, AKT2, SYK	0.001218
Hepatitis C	PIK3CG, PPARA, LDLR, RXRA, NFKBIA, NFKB1, KRAS, MAPK14, MAPK3, CD81, SCARB1, EGF, AKT2, NR1H3	0.001755
Influenza A	PRKCA, PIK3CG, IL18, FDPS, NFKBIA, NLRX1, HSPA1A, NFKB1, HSPA1B, NLRP3, PRKCB, MAPK14, MAPK3, PYCARD, FAS, AKT2	0.002805

## Data Availability

The data used in this study are available at the following links: TCMSP: http://lsp.nwu.edu.cn/tcmsp.php; BATMAN-TCM: http://bionet.ncpsb.org/batman-tcm/; CTD: http://ctdbase.org/; GeneCards: http://www.genecards.org/; Draw Venn diagrams: http://bioinformatics.psb.ugent.be/webtools/Venn/;STRING database: https://string-db.org/; DAVID ver 6.8: https://david.ncifcrf.gov/;OmicShare tools:https://www.omicshare.com/tools.

## Abbreviations

SXZKF: Sang-Xing-Zhi-Ke-Fang
TCMSP: Traditional Chinese Medicine Systems Pharmacology Database and Analysis Platform
BATMAN-TCM: Bioinformatics Analysis Tool for Molecular mechANism of Traditional Chinese Medicine
UniProt: Universal Protein Database
CTD: Comparative Toxicogenomics Database
GeneCards: Human Gene Database
STRING: Search Tool for the Retrieval of Interacting Genes
PPI: Protein-protein interactions
DC: Degree centrality
BC: Betweenness centrality
CC: Closeness centrality
DAVID: Database for Annotation, Visualization, and Integrated Discovery
KEGG: Kyoto Encyclopedia of Genes and Genomes
OB: Oral bioavailability
DL: Drug-likeness
ALB: Serum albumin
PPAR*γ*: Peroxisome proliferator-activated receptor gamma
MAPK3: Mitogen-activated protein kinase 3
EGF: Epidermal growth factor
PTGS2: Prostaglandin G/H synthase 2
VEGF: Vascular endothelial growth factor
PI3K-AKt: Phosphatidylinositol-3-kinase-protein kinase B.

## Supplementary Materials

Supplementary Materials Table S1: 102 bioactive compounds obtained and screened out from TCMSP, BATMAN-TCM, and literature. Table S2: 886 targets of bioactive compounds collected using TCMSP and UniProt. Table S3: targets related to pharyngitis, including 5150 targets from CTD and 1803 targets from GeneCards with 695 targets duplicated. Table S4: 387 overlapping targets related to 19 bioactive compounds. Table S5: the results of topological features of the PPI network, including the values of topological features of 354 targets, while the other 33 targets were unrelated to each other target in the network. Table S6: the results of KEGG pathway enrichment, including 43 KEGG pathways were recognized as *P* < 0.05 with 28 pathways being recognized as *P* < 0.01.
