# Supplementary material for: Network Pharmacology Identifies the Mechanisms of Sang-Xing-Zhi-Ke-Fang against Pharyngitis
Source: Evid Based Complement Alternat Med. 2020 Oct 12;2020:2421916. doi: 10.1155/2020/2421916 (PMC7576344; doi:10.1155/2020/2421916)
Supplement: Supplementary Materials — Table S1: 102 bioactive compounds obtained and screened out from TCMSP, BATMAN-TCM, and literature. Table S2: 886 targets of bioactive compounds collected using TCMSP and UniProt. Table S3: targets related to pharyngitis, including 5150 targets from CTD and 1803 targets from GeneCards with 695 targets duplicated. Table S4: 387 overlapping targets related to 19 bioactive compounds. Table S5: the results of topological features of the PPI network, including the values of topological features of 354 targets, while the other 33 targets were unrelated to each other target in the network. Table S6: the results of KEGG pathway enrichment, including 43 KEGG pathways were recognized as P < 0.05 with 28 pathways being recognized as P < 0.01. [file 2421916.f1.zip › Supplementary materials/Suppplementary Table S1.docx]

| Molecule Name | Herb | OB (%) | DL |
| --- | --- | --- | --- |
| Supraene | Mulberry Leaf | 33.55 | 0.42 |
| poriferast-5-en-3beta-ol | Mulberry Leaf | 36.91 | 0.75 |
| beta-sitosterol | Mulberry Leaf\Radix Glehniae\Loguat Leaf\Fritillary Bulb\Exocarpium Citrus Grandis | 36.91 | 0.75 |
| beta-carotene | Mulberry Leaf | 37.18 | 0.58 |
| Skimmin (8CI) | Mulberry Leaf | 38.35 | 0.32 |
| Inophyllum E | Mulberry Leaf | 38.81 | 0.85 |
| Isoramanone | Mulberry Leaf | 39.97 | 0.51 |
| 4-Prenylresveratrol | Mulberry Leaf | 40.54 | 0.21 |
| kaempferol | Mulberry Leaf\Loguat Leaf | 41.88 | 0.24 |
| Tetramethoxyluteolin | Mulberry Leaf | 43.68 | 0.37 |
| Stigmasterol | Mulberry Leaf\Semen Armeniacae Amarum\Radix Glehniae\Radix Rehmanniae Praeparata | 43.83 | 0.76 |
| 26-Hydroxy-dammara-20,24-dien-3-one | Mulberry Leaf | 44.41 | 0.79 |
| icosa-11,14,17-trienoic acid methyl ester | Mulberry Leaf | 44.81 | 0.23 |
| arachidonic acid | Mulberry Leaf\Earthworm | 45.57 | 0.2 |
| Linolenic acid ethyl ester | Mulberry Leaf | 46.1 | 0.2 |
| quercetin | Mulberry Leaf\Radix Glehniae\Loguat Leaf | 46.43 | 0.28 |
| Oxysanguinarine | Mulberry Leaf | 46.97 | 0.87 |
| Moracin F | Mulberry Leaf | 53.81 | 0.23 |
| Norartocarpetin | Mulberry Leaf | 54.93 | 0.24 |
| Moracin B | Mulberry Leaf | 55.85 | 0.23 |
| Moracin E | Mulberry Leaf | 56.08 | 0.38 |
| scopolin | Mulberry Leaf | 56.45 | 0.39 |
| Moracin D | Mulberry Leaf | 60.93 | 0.38 |
| Iristectorigenin A | Mulberry Leaf | 63.36 | 0.34 |
| FA | Mulberry Leaf\Rhizoma Chuanxiong | 68.96 | 0.71 |
| Moracin H | Mulberry Leaf | 74.35 | 0.51 |
| Moracin G | Mulberry Leaf | 75.78 | 0.42 |
| Moracin C | Mulberry Leaf | 82.13 | 0.29 |
| Albanol | Mulberry Leaf | 83.16 | 0.24 |
| gondoic acid | Semen Armeniacae Amarum | 30.7 | 0.2 |
| Diisooctyl succinate | Semen Armeniacae Amarum | 31.62 | 0.23 |
| (6Z,10E,14E,18E)-2,6,10,15,19,23-hexamethyltetracosa-2,6,10,14,18,22-hexaene | Semen Armeniacae Amarum | 33.55 | 0.42 |
| sitosterol | Semen Armeniacae Amarum\Radix Rehmanniae Praeparata\Exocarpium Citrus Grandis\Rhizoma Chuanxiong | 36.91 | 0.75 |
| CLR | Semen Armeniacae Amarum | 37.87 | 0.68 |
| 11,14-eicosadienoic acid | Semen Armeniacae Amarum | 39.99 | 0.2 |
| Spinasterol | Semen Armeniacae Amarum | 42.98 | 0.76 |
| Glabridin | Semen Armeniacae Amarum | 53.25 | 0.47 |
| estrone | Semen Armeniacae Amarum | 53.56 | 0.32 |
| (+)-catechin | Semen Armeniacae Amarum | 54.83 | 0.24 |
| Mairin | Semen Armeniacae Amarum\Loguat Leaf | 55.38 | 0.78 |
| liquiritin | Semen Armeniacae Amarum | 65.69 | 0.74 |
| Ziziphin_qt | Semen Armeniacae Amarum | 66.95 | 0.62 |
| Licochalcone B | Semen Armeniacae Amarum | 76.76 | 0.19 |
| Phaseol | Semen Armeniacae Amarum | 78.77 | 0.58 |
| Machiline | Semen Armeniacae Amarum | 79.64 | 0.24 |
| l-SPD | Semen Armeniacae Amarum | 87.35 | 0.54 |
| Glycyrol | Semen Armeniacae Amarum | 90.78 | 0.67 |
| Alloisoimperatorin | Radix Glehniae | 34.8 | 0.22 |
| Ammidin | Radix Glehniae | 34.55 | 0.22 |
| isoimperatorin | Radix Glehniae | 45.46 | 0.23 |
| Cnidilin | Radix Glehniae | 32.69 | 0.28 |
| Bergaptin | Radix Glehniae | 41.73 | 0.42 |
| Cinchonain 1a | Loguat Leaf | 30.12 | 0.93 |
| (4S,8R,9R)-4,8-bis(3,4-dihydroxyphenyl)-5,9-dihydroxy-4,8,9,10-tetrahydro-3H-pyrano[6,5-h]chromen-2-one | Loguat Leaf | 31.32 | 0.93 |
| (4aS,6aR,6aS,6bR,8aR,10R,12aR,14bS)-10-hydroxy-2,2,6a,6b,9,9,12a-heptamethyl-1,3,4,5,6,6a,7,8,8a,10,11,12,13,14b-tetradecahydropicene-4a-carboxylic acid | Loguat Leaf | 32.03 | 0.76 |
| Torulene | Loguat Leaf | 33.49 | 0.55 |
| 23-trans-p-coumaryhormentic acid | Loguat Leaf | 36.08 | 0.32 |
| isohumbertiol-3-o-{α-L-rhamnopyranosyl(1→4)-α-L-rhamnopyranosyl(1→2)-[α-L-rhamnopyranosyl(1→6)]}-β-D-glucopyranoside | Loguat Leaf | 36.75 | 0.59 |
| daucosterol_qt | Loguat Leaf | 36.91 | 0.75 |
| 7-[[(1S,4aS,6R,8aR)-6-hydroxy-5,5,8a-trimethyl-2-methylene-decalin-1-yl]methoxy]coumarin | Loguat Leaf | 42.36 | 0.64 |
| ellagic acid | Loguat Leaf | 43.06 | 0.43 |
| isorhamnetin | Loguat Leaf | 49.6 | 0.31 |
| (-)-epigallocatechin-3-gallate | Loguat Leaf | 55.09 | 0.77 |
| (4R,8R,9R)-4,8-bis(3,4-dihydroxyphenyl)-5,9-dihydroxy-4,8,9,10-tetrahydro-3H-pyrano[6,5-h]chromen-2-one | Loguat Leaf | 58.16 | 0.93 |
| (2R,3R,10S)-2,10-bis(3,4-dihydroxyphenyl)-3,5-dihydroxy-3,4,9,10-tetrahydro-2H-pyrano[6,5-h]chromen-8-one | Loguat Leaf | 65.26 | 0.93 |
| (2R,3R,4S)-2-(3,4-dihydroxyphenyl)-4-(2,4,6-trihydroxyphenyl)chroman-3,5,7-triol | Loguat Leaf | 72.41 | 0.64 |
| neohesperidin_qt | Exocarpium Citrus Grandis | 71.17 | 0.27 |
| nobiletin | Exocarpium Citrus Grandis | 61.67 | 0.52 |
| naringenin | Exocarpium Citrus Grandis\Exocarpium Citrus Grandis | 59.29 | 0.21 |
| Isosinensetin | Exocarpium Citrus Grandis | 51.15 | 0.44 |
| Sinensetin | Exocarpium Citrus Grandis | 50.56 | 0.45 |
| 5,7,4'-Trimethylapigenin | Exocarpium Citrus Grandis | 39.83 | 0.3 |
| didymin | Exocarpium Citrus Grandis | 38.55 | 0.24 |
| poncirin | Exocarpium Citrus Grandis | 36.55 | 0.74 |
| LYC | Exocarpium Citrus Grandis | 32.57 | 0.51 |
| pelargonidin | Fritillary Bulb | 37.99 | 0.21 |
| Peimisine | Fritillary Bulb | 57.4 | 0.81 |
| Zhebeiresinol | Fritillary Bulb | 58.72 | 0.19 |
| Ziebeimine | Fritillary Bulb | 64.25 | 0.7 |
| 6-Methoxyl-2-acetyl-3-methyl-1,4-naphthoquinone-8-O-beta-D-glucopyranoside | Fritillary Bulb | 33.31 | 0.57 |
| Chaksine | Fritillary Bulb | 65.63 | 0.66 |
| Myricanone | Rhizoma Chuanxiong | 40.6 | 0.51 |
| Mandenol | Rhizoma Chuanxiong | 42 | 0.19 |
| wallichilide | Rhizoma Chuanxiong | 42.31 | 0.71 |
| senkyunone | Rhizoma Chuanxiong | 47.66 | 0.24 |
| Perlolyrine | Rhizoma Chuanxiong | 65.95 | 0.27 |
| Fortunellin | Exocarpium Citrus Grandis | 35.65 | 0.74 |
| acacetin | Exocarpium Citrus Grandis | 34.97 | 0.24 |
| Linarin | Exocarpium Citrus Grandis | 39.84 | 0.71 |
| Diosmetin | Exocarpium Citrus Grandis | 31.14 | 0.27 |
| aloe-emodin | Exocarpium Citrus Grandis | 83.38 | 0.24 |
| eriodictyol | Exocarpium Citrus Grandis | 71.79 | 0.24 |
| Genkwanin | Exocarpium Citrus Grandis | 37.13 | 0.24 |
| luteolin | Exocarpium Citrus Grandis | 36.16 | 0.25 |
| Dihydrocapsaicin | Earthworm | 47.07 | 0.19 |
| Timnodonic acid | Earthworm | 45.66 | 0.21 |
| arachidonate | Earthworm | 45.57 | 0.2 |
| 8, 11, 14-eicosatrienoic acid | Earthworm | 44.11 | 0.2 |
| Eicosapentaenoic acid | Earthworm | 45.66 | 0.21 |
| Eicosatrienoic acid | Earthworm | 44.11 | 0.2 |
| alizarin | Earthworm | 32.67 | 0.19 |
| cholesterol | Earthworm | 37.87 | 0.68 |
