# Supplementary material for: Network Pharmacology Identifies the Mechanisms of Sang-Xing-Zhi-Ke-Fang against Pharyngitis
Source: Evid Based Complement Alternat Med. 2020 Oct 12;2020:2421916. doi: 10.1155/2020/2421916 (PMC7576344; doi:10.1155/2020/2421916)
Supplement: Supplementary Materials — Table S1: 102 bioactive compounds obtained and screened out from TCMSP, BATMAN-TCM, and literature. Table S2: 886 targets of bioactive compounds collected using TCMSP and UniProt. Table S3: targets related to pharyngitis, including 5150 targets from CTD and 1803 targets from GeneCards with 695 targets duplicated. Table S4: 387 overlapping targets related to 19 bioactive compounds. Table S5: the results of topological features of the PPI network, including the values of topological features of 354 targets, while the other 33 targets were unrelated to each other target in the network. Table S6: the results of KEGG pathway enrichment, including 43 KEGG pathways were recognized as P < 0.05 with 28 pathways being recognized as P < 0.01. [file 2421916.f1.zip › Supplementary materials/Suppplementary Table S2.docx]

| **NO.** | **Gene** | **No.** | **Gene** | **No.** | **Gene** | **No.** | **Gene** | **No.** | **Gene** |
| --- | --- | --- | --- | --- | --- | --- | --- | --- | --- |
| 1 | **NPC2** | 179 | **AGMO** | 357 | **CIAO2A** | 535 | **NPC1L1** | 713 | **FABP1** |
| 2 | **BCO2** | 180 | **FAM83A** | 358 | **DEGS1** | 536 | **GBA** | 714 | **OSBPL10** |
| 3 | **BCDO2** | 181 | **FAM3A** | 359 | **FAM20A** | 537 | **GBA2** | 715 | **EBP** |
| 4 | **BCO1** | 182 | **HLA-A** | 360 | **FRMD5** | 538 | **CEL** | 716 | **LDLR** |
| 5 | **Pix-1** | 183 | **CCNQ** | 361 | **FAAP24** | 539 | **NCEH1** | 717 | **HDAC9** |
| 6 | **MTRNR2L2** | 184 | **AEBP1** | 362 | **FAM53C** | 540 | **CES1** | 718 | **PROM1** |
| 7 | **MTRNR2L4** | 185 | **TMEM185A** | 363 | **EPB41L1** | 541 | **ABCG1** | 719 | **FGF19** |
| 8 | **MTRNR2L8** | 186 | **LCN2** | 364 | **MINDY2** | 542 | **APOA1** | 720 | **FGF1** |
| 9 | **MTRNR2L7** | 187 | **TPRG1L** | 365 | **ORM1** | 543 | **DHCR24** | 721 | **ERLIN1** |
| 10 | **MTRNR2L6** | 188 | **TENT5B** | 366 | **FAM53B** | 544 | **APOE** | 722 | **PLA2G15** |
| 11 | **MTRNR2L9** | 189 | **WASHC2A** | 367 | **FAM71B** | 545 | **STARD3** | 723 | **LDAH** |
| 12 | **MTRNR2L3** | 190 | **CNTNAP4** | 368 | **FAM89A** | 546 | **NFE2L1** | 724 | **KL** |
| 13 | **MTRNR2L1** | 191 | **ABCD3** | 369 | **F9** | 547 | **COMMD9** | 725 | **LBR** |
| 14 | **MTRNR2L10** | 192 | **PRMT5** | 370 | **MIGA2** | 548 | **ASAH2** | 726 | **KLB** |
| 15 | **MTRNR2L5** | 193 | **CFB** | 371 | **IL1F10** | 549 | **ANXA2** | 727 | **ABCA12** |
| 16 | **rbcL** | 194 | **EPB41** | 372 | **FAM49B** | 550 | **ADRB2** | 728 | **APOC2** |
| 17 | **matK** | 195 | **B2M** | 373 | **LCN1** | 551 | **ABCA2** | 729 | **DGAT2** |
| 18 | **IPMK** | 196 | **COX20** | 374 | **FANCD2** | 552 | **ACOX1** | 730 | **ABCG8** |
| 19 | **DHRS11** | 197 | **CERS2** | 375 | **FAM50B** | 553 | **ACLY** | 731 | **ERLIN2** |
| 20 | **DHRS4** | 198 | **CAMP** | 376 | **FAXDC2** | 554 | **ANPEP** | 732 | **PMVK** |
| 21 | **IP6K2** | 199 | **C8G** | 377 | **FAM49A** | 555 | **BAAT** | 733 | **NR0B2** |
| 22 | **CYP4F2** | 200 | **FADS3** | 378 | **FAM43B** | 556 | **FDX1** | 734 | **NR1I3** |
| 23 | **CYP2J2** | 201 | **CENPX** | 379 | **CPXM1** | 557 | **ACADVL** | 735 | **NR1H3** |
| 24 | **KCNK4** | 202 | **CSF1** | 380 | **FRMD3** | 558 | **APOA4** | 736 | **NR1I2** |
| 25 | **CYP4A11** | 203 | **FAM83C** | 381 | **BOD1L2** | 559 | **CETP** | 737 | **NSDHL** |
| 26 | **FABP2** | 204 | **DDR1** | 382 | **FAM47B** | 560 | **CCR5** | 738 | **NR1H2** |
| 27 | **FABP4** | 205 | **RBP1** | 383 | **FAM71A** | 561 | **ACACA** | 739 | **NFKB1** |
| 28 | **CYP4A22** | 206 | **RMDN1** | 384 | **SSPO** | 562 | **SMPDL3A** | 740 | **MBTPS2** |
| 29 | **FADS1** | 207 | **RDH5** | 385 | **TMEM236** | 563 | **SMPD1** | 741 | **INSIG2** |
| 30 | **CYP4F3** | 208 | **TENT5C** | 386 | **TLCD3B** | 564 | **C5AR2** | 742 | **LPL** |
| 31 | **FAAH** | 209 | **GTF2F1** | 387 | **CALHM5** | 565 | **ANXA9** | 743 | **MTTP** |
| 32 | **PLA2G4A** | 210 | **FAM20B** | 388 | **CNTNAP5** | 566 | **CYP7B1** | 744 | **MIA2** |
| 33 | **CYP2C9** | 211 | **SC5D** | 389 | **C6orf52** | 567 | **CD24** | 745 | **PLSCR3** |
| 34 | **CYP1B1** | 212 | **TMEM8B** | 390 | **FAM87A** | 568 | **ACACB** | 746 | **PON1** |
| 35 | **PLA2G10** | 213 | **RBP4** | 391 | **NKAIN4** | 569 | **CD36** | 747 | **PLTP** |
| 36 | **S100A9** | 214 | **PMP2** | 392 | **LCN8** | 570 | **AACS** | 748 | **LIPA** |
| 37 | **PPP5C** | 215 | **LRATD2** | 393 | **LCNL1** | 571 | **CAT** | 749 | **MALRD1** |
| 38 | **S100A8** | 216 | **RIPOR3** | 394 | **NUTM2A** | 572 | **APOL2** | 750 | **OSBPL5** |
| 39 | **CYP1A1** | 217 | **FAM89B** | 395 | **TMEM255B** | 573 | **GRAMD1C** | 751 | **LRP5L** |
| 40 | **CYP2C8** | 218 | **OBP2A** | 396 | **TMEM255A** | 574 | **ACADL** | 752 | **IDI2** |
| 41 | **ALOXE3** | 219 | **LEPR** | 397 | **NKAIN3** | 575 | **PRKAA2** | 753 | **LIPC** |
| 42 | **ALOX5** | 220 | **PYGL** | 398 | **TVP23A** | 576 | **CLEC4E** | 754 | **NR1D1** |
| 43 | **PLA2G2F** | 221 | **RBP2** | 399 | **TPRG1** | 577 | **CHGA** | 755 | **MBTPS1** |
| 44 | **PLA2R1** | 222 | **PALLD** | 400 | **NKAIN1** | 578 | **APOA2** | 756 | **MT3** |
| 45 | **ALOX5AP** | 223 | **DCBLD2** | 401 | **OBP2B** | 579 | **AGTR1** | 757 | **CYB5RL** |
| 46 | **ALOX15** | 224 | **RNF8** | 402 | **WASH2P** | 580 | **CEBPA** | 758 | **OSBP2** |
| 47 | **CYP1A2** | 225 | **AHCTF1** | 403 | **IFI27L2** | 581 | **LDLRAP1** | 759 | **APOBR** |
| 48 | **DRD2** | 226 | **LSM14A** | 404 | **FAM78B** | 582 | **APOF** | 760 | **MVD** |
| 49 | **AWAT1** | 227 | **IFI27L1** | 405 | **FAM98C** | 583 | **GRAMD1B** | 761 | **PCSK9** |
| 50 | **CREB1** | 228 | **CRABP1** | 406 | **FAM81A** | 584 | **CLN8** | 762 | **OSBPL2** |
| 51 | **ABHD2** | 229 | **SINHCAF** | 407 | **FAM45A** | 585 | **CYP39A1** | 763 | **PLPP6** |
| 52 | **ANXA1** | 230 | **SHLD2** | 408 | **FAM47A** | 586 | **ACSM1** | 764 | **LIMA1** |
| 53 | **CYP4F12** | 231 | **FAM86C1** | 409 | **FADS6** | 587 | **ANXA6** | 765 | **OSBPL8** |
| 54 | **CYP2A13** | 232 | **FAM98B** | 410 | **LCN12** | 588 | **ABCG4** | 766 | **G6PD** |
| 55 | **CYP2U1** | 233 | **MAPK14** | 411 | **LCN9** | 589 | **APOL1** | 767 | **MSR1** |
| 56 | **CPTP** | 234 | **RMDN3** | 412 | **FABP12** | 590 | **APOL4** | 768 | **OSBP** |
| 57 | **CYP2B6** | 235 | **PIEZO2** | 413 | **FAM76A** | 591 | **VDR** | 769 | **DAG1** |
| 58 | **CYP2E1** | 236 | **RBP5** | 414 | **DIPK1C** | 592 | **USF1** | 770 | **MED15** |
| 59 | **GPR132** | 237 | **PTPN4** | 415 | **EPB41L4B** | 593 | **VLDLR** | 771 | **APOA5** |
| 60 | **CYP2A6** | 238 | **GAREM1** | 416 | **FAM72D** | 594 | **VAT1L** | 772 | **MLC1** |
| 61 | **CYP2C19** | 239 | **PTGDS** | 417 | **CALHM6** | 595 | **SCARB2** | 773 | **CYP51A1** |
| 62 | **CYP2C18** | 240 | **CH25H** | 418 | **CNTNAP3** | 596 | **SGMS2** | 774 | **CAVIN3** |
| 63 | **ACE** | 241 | **FANCF** | 419 | **NUTM2F** | 597 | **XBP1** | 775 | **ANGPTL3** |
| 64 | **PIBF1** | 242 | **WASHC2C** | 420 | **CALHM2** | 598 | **TSPO** | 776 | **IFITM3** |
| 65 | **PTGS1** | 243 | **RS1** | 421 | **CALHM4** | 599 | **VAPA** | 777 | **NFKBIA** |
| 66 | **RAC2** | 244 | **RBP7** | 422 | **NXPE1** | 600 | **RORA** | 778 | **EDN1** |
| 67 | **MBOAT7** | 245 | **MTFR2** | 423 | **NXPE3** | 601 | **SYCN** | 779 | **G6PC** |
| 68 | **MGLL** | 246 | **PIMREG** | 424 | **NXPE4** | 602 | **SREBF2** | 780 | **MYO5B** |
| 69 | **KCNK18** | 247 | **SKA2** | 425 | **CPXM2** | 603 | **TSPO2** | 781 | **PROM2** |
| 70 | **NCF2** | 248 | **STRIP1** | 426 | **FAM78A** | 604 | **SLC27A5** | 782 | **GPLD1** |
| 71 | **ALB** | 249 | **RIPOR1** | 427 | **FAM47C** | 605 | **STX12** | 783 | **OSBPL6** |
| 72 | **PPARG** | 250 | **SUPT20H** | 428 | **FAM43A** | 606 | **TTC39B** | 784 | **OSBPL7** |
| 73 | **PLA2G4B** | 251 | **TMEM8A** | 429 | **CNTNAP3B** | 607 | **TGFBR2** | 785 | **PI4K2A** |
| 74 | **PLA2G12B** | 252 | **STRIP2** | 430 | **FAM72B** | 608 | **NFYC** | 786 | **OSBPL9** |
| 75 | **PLA2G5** | 253 | **RUNX1** | 431 | **FAM71D** | 609 | **NCOA2** | 787 | **EPHX2** |
| 76 | **KCNK10** | 254 | **RNF168** | 432 | **DIPK1A** | 610 | **NR5A2** | 788 | **ARHGAP45** |
| 77 | **NTSR1** | 255 | **TVP23B** | 433 | **FAM47E** | 611 | **THBS4** | 789 | **LIPG** |
| 78 | **PLA2G12A** | 256 | **TLCD3A** | 434 | **CELSR1** | 612 | **TMEM199** | 790 | **KPNB1** |
| 79 | **RAC1** | 257 | **TENT5D** | 435 | **CLIP3** | 613 | **UBIAD1** | 791 | **CYB5R1** |
| 80 | **SLCO2A1** | 258 | **SCD5** | 436 | **ANAPC2** | 614 | **IDI1** | 792 | **OSBPL1A** |
| 81 | **PHLPP2** | 259 | **RMDN2** | 437 | **C1R** | 615 | **SCUBE2** | 793 | **FDFT1** |
| 82 | **TRPM2** | 260 | **RIPOR2** | 438 | **NLRC3** | 616 | **TRERF1** | 794 | **SLC16A11** |
| 83 | **STX3** | 261 | **PLAAT3** | 439 | **NLRX1** | 617 | **STOML1** | 795 | **LRP8** |
| 84 | **PLA2G2D** | 262 | **TMEM185B** | 440 | **NLRC5** | 618 | **SLC27A2** | 796 | **IL18** |
| 85 | **PTGS2** | 263 | **TENT5A** | 441 | **PYDC2** | 619 | **SLC13A5** | 797 | **MVK** |
| 86 | **DAGLA** | 264 | **LPGAT1** | 442 | **CALR** | 620 | **TMEM97** | 798 | **INSIG1** |
| 87 | **DAGLB** | 265 | **DCBLD1** | 443 | **PYCARD** | 621 | **SREBF1** | 799 | **ANGPTL8** |
| 88 | **BDKRB2** | 266 | **MINDY1** | 444 | **CELSR2** | 622 | **SP1** | 800 | **DISP1** |
| 89 | **CYP2F1** | 267 | **LSM14B** | 445 | **CELSR3** | 623 | **SEC24C** | 801 | **FDPS** |
| 90 | **HSPA1B** | 268 | **PIEZO1** | 446 | **NLRP3** | 624 | **STARD4** | 802 | **FGFR4** |
| 91 | **NMB** | 269 | **GAREM2** | 447 | **C1S** | 625 | **TGFBR1** | 803 | **CCL3** |
| 92 | **PLA2G3** | 270 | **FANCL** | 448 | **CCSMST1** | 626 | **SEC24D** | 804 | **CD209** |
| 93 | **PNPLA8** | 271 | **FAM50A** | 449 | **GOLGA6L9** | 627 | **VPS4A** | 805 | **CAV1** |
| 94 | **ATP5PF** | 272 | **WDR77** | 450 | **STS** | 628 | **SCG3** | 806 | **AGT** |
| 95 | **CYP2S1** | 273 | **POR** | 451 | **SLC22A11** | 629 | **HDLBP** | 807 | **ABCA4** |
| 96 | **PLA2G1B** | 274 | **CIAO2B** | 452 | **CYP3A7** | 630 | **LANCL2** | 808 | **ABCA5** |
| 97 | **NMUR2** | 275 | **FAM3B** | 453 | **SULT1E1** | 631 | **HNF4A** | 809 | **ABCA6** |
| 98 | **PLA2G4C** | 276 | **FXN** | 454 | **CYP3A5** | 632 | **MYLIP** | 810 | **CD44** |
| 99 | **PLA2G2E** | 277 | **C5orf30** | 455 | **CYP19A1** | 633 | **SEC14L2** | 811 | **ABCA10** |
| 100 | **PLA2G6** | 278 | **ESYT1** | 456 | **HSD17B12** | 634 | **SGMS1** | 812 | **APOC1** |
| 101 | **ALOX12B** | 279 | **FABP3** | 457 | **HSD17B2** | 635 | **SMO** | 813 | **CYP11B2** |
| 102 | **ALOX12** | 280 | **EPB41L3** | 458 | **HSD17B6** | 636 | **SORT1** | 814 | **PRKAA1** |
| 103 | **MAPKAPK2** | 281 | **EPB41L2** | 459 | **SLCO1B1** | 637 | **SERAC1** | 815 | **HSD3B2** |
| 104 | **MAPK3** | 282 | **FADS2** | 460 | **SLCO3A1** | 638 | **SCP2D1** | 816 | **DGKQ** |
| 105 | **HSPA1A** | 283 | **ESYT2** | 461 | **HSD17B7** | 639 | **LRP6** | 817 | **DGAT1** |
| 106 | **CYP2D6** | 284 | **FAM81B** | 462 | **HSD17B1** | 640 | **RTKN2** | 818 | **DPP4** |
| 107 | **DRD3** | 285 | **FAM83E** | 463 | **HSD17B8** | 641 | **STAR** | 819 | **GPIHBP1** |
| 108 | **DRD4** | 286 | **BARD1** | 464 | **HSD17B11** | 642 | **INHBA** | 820 | **DHDDS** |
| 109 | **PKN3** | 287 | **CALHM3** | 465 | **ESR1** | 643 | **NUS1** | 821 | **LMNA** |
| 110 | **HSP90AA1** | 288 | **FAM71C** | 466 | **AKR1B15** | 644 | **SHH** | 822 | **LRP5** |
| 111 | **PLA2G2A** | 289 | **CPAMD8** | 467 | **SLC47A1** | 645 | **SCAP** | 823 | **DISP3** |
| 112 | **CYP3A4** | 290 | **FAM156A** | 468 | **SLC22A20P** | 646 | **STARD6** | 824 | **HMGCS2** |
| 113 | **SYK** | 291 | **HSPB11** | 469 | **SLC22A8** | 647 | **SCARF1** | 825 | **CYP27A1** |
| 114 | **MIF** | 292 | **FRMD7** | 470 | **HSD3B1** | 648 | **ABCC6** | 826 | **CLU** |
| 115 | **ALOX15B** | 293 | **BTBD9** | 471 | **SLCO1C1** | 649 | **STARD3NL** | 827 | **EHD1** |
| 116 | **FAAH2** | 294 | **FAM3C** | 472 | **SLC10A7** | 650 | **RAB11A** | 828 | **LSS** |
| 117 | **SSTR4** | 295 | **EDIL3** | 473 | **SLC22A1** | 651 | **SYT7** | 829 | **GNPTAB** |
| 118 | **CYP2A7** | 296 | **FABP7** | 474 | **SLCO4A1** | 652 | **SYP** | 830 | **ABCG5** |
| 119 | **KCNK13** | 297 | **FABP9** | 475 | **SULT4A1** | 653 | **SLC27A1** | 831 | **PNLIPRP1** |
| 120 | **TRPM5** | 298 | **EPB41L5** | 476 | **SLCO1A2** | 654 | **GPS2** | 832 | **ANGPTL4** |
| 121 | **OXER1** | 299 | **EEF2KMT** | 477 | **SLC22A7** | 655 | **TGFB1** | 833 | **ANXA2P2** |
| 122 | **OC90** | 300 | **AMBP** | 478 | **SLC47A2** | 656 | **PDZK1** | 834 | **APP** |
| 123 | **PROCA1** | 301 | **FAM9C** | 479 | **SHBG** | 657 | **EHD2** | 835 | **GRAMD1A** |
| 124 | **GPR31** | 302 | **DEGS2** | 480 | **ABCC11** | 658 | **SLC10A2** | 836 | **CUBN** |
| 125 | **PLA2G4F** | 303 | **FAM72A** | 481 | **ESR2** | 659 | **LMF1** | 837 | **DHCR7** |
| 126 | **CBR1** | 304 | **HES1** | 482 | **CYP11B1** | 660 | **APOO** | 838 | **LAMTOR1** |
| 127 | **ABCG2** | 305 | **NXPE2** | 483 | **SLC17A3** | 661 | **TFCP2L1** | 839 | **EGF** |
| 128 | **ABCC2** | 306 | **PAEP** | 484 | **SLC22A6** | 662 | **GULP1** | 840 | **CLN6** |
| 129 | **PIM1** | 307 | **NRP2** | 485 | **CA2** | 663 | **HMGCS1** | 841 | **IL4** |
| 130 | **PIK3CG** | 308 | **APOD** | 486 | **PRKACA** | 664 | **PNLIP** | 842 | **MED13** |
| 131 | **PIR** | 309 | **FABP5** | 487 | **CA4** | 665 | **UBE3B** | 843 | **CD81** |
| 132 | **SLC16A1** | 310 | **MTFR1L** | 488 | **CA1** | 666 | **SAR1B** | 844 | **ABCB4** |
| 133 | **HCK** | 311 | **CALHM1** | 489 | **CSNK2A1** | 667 | **CAV3** | 845 | **HMGCR** |
| 134 | **STK17B** | 312 | **HADHA** | 490 | **CA3** | 668 | **MALL** | 846 | **GNB3** |
| 135 | **SLCO2B1** | 313 | **FAM76B** | 491 | **CA12** | 669 | **LIPE** | 847 | **NR3C1** |
| 136 | **SIRT1** | 314 | **FAM13C** | 492 | **PRKCB** | 670 | **SEC23A** | 848 | **GPAM** |
| 137 | **ABCC1** | 315 | **FARP2** | 493 | **CA9** | 671 | **SNX17** | 849 | **GRHL1** |
| 138 | **ATP5F1A** | 316 | **FAM3D** | 494 | **PRKCA** | 672 | **UBP1** | 850 | **ACSM3** |
| 139 | **ABCB1** | 317 | **ABCD1** | 495 | **CA14** | 673 | **VPS4B** | 851 | **ABCA9** |
| 140 | **ATP5F1B** | 318 | **FARP1** | 496 | **CA6** | 674 | **VPS51** | 852 | **ACOX2** |
| 141 | **HIBCH** | 319 | **FAM92B** | 497 | **SQLE** | 675 | **SGPL1** | 853 | **ADIPOQ** |
| 142 | **ATP5F1C** | 320 | **FAAP100** | 498 | **CA5A** | 676 | **RALY** | 854 | **ABCA3** |
| 143 | **SLC16A7** | 321 | **FAM9B** | 499 | **CA7** | 677 | **RAN** | 855 | **CNR1** |
| 144 | **UGT3A1** | 322 | **FAM9A** | 500 | **CA5B** | 678 | **STARD5** | 856 | **DHH** |
| 145 | **GBA3** | 323 | **FAM83B** | 501 | **SPACA3** | 679 | **SPRED1** | 857 | **CFTR** |
| 146 | **FA2H** | 324 | **FAM13B** | 502 | **LYZ** | 680 | **SCARB1** | 858 | **AKR1C1** |
| 147 | **DLK1** | 325 | **FAM92A** | 503 | **LYZL6** | 681 | **SMAD2** | 859 | **GHR** |
| 148 | **HIST1H4A** | 326 | **MIGA1** | 504 | **LYZL4** | 682 | **SULT2B1** | 860 | **ATAD3A** |
| 149 | **ELOVL3** | 327 | **NRP1** | 505 | **LYZL1** | 683 | **SERPINA12** | 861 | **FASN** |
| 150 | **ELOVL1** | 328 | **FFAR4** | 506 | **ACSL1** | 684 | **SIGMAR1** | 862 | **CNBP** |
| 151 | **ELOVL2** | 329 | **AKR1C3** | 507 | **ACSL4** | 685 | **SEC24B** | 863 | **APOM** |
| 152 | **ELOVL4** | 330 | **MFGE8** | 508 | **ACSL3** | 686 | **RAB11FIP2** | 864 | **AKR1D1** |
| 153 | **ELOVL7** | 331 | **HAUS6** | 509 | **ACSBG2** | 687 | **6-Mar** | 865 | **ABCA7** |
| 154 | **ELOVL6** | 332 | **FAM98A** | 510 | **ACSL6** | 688 | **ACAT1** | 866 | **APOB** |
| 155 | **ELOVL5** | 333 | **DIPK1B** | 511 | **ACSL5** | 689 | **ACAA2** | 867 | **ARL4C** |
| 156 | **HIST1H3A** | 334 | **FAM83D** | 512 | **CYP4F11** | 690 | **SIDT1** | 868 | **FDXR** |
| 157 | **F7** | 335 | **FAM20C** | 513 | **DGKE** | 691 | **SIDT2** | 869 | **AKT2** |
| 158 | **F11** | 336 | **EPB41L4A** | 514 | **CYP4F8** | 692 | **OSBPL3** | 870 | **GPX4** |
| 159 | **F10** | 337 | **LCN15** | 515 | **ABHD6** | 693 | **SEC24A** | 871 | **SCD** |
| 160 | **F13A1** | 338 | **FFAR1** | 516 | **ABHD12** | 694 | **MREG** | 872 | **GGPS1** |
| 161 | **F12** | 339 | **HMGXB4** | 517 | **LPCAT3** | 695 | **RNF145** | 873 | **APOC3** |
| 162 | **F2** | 340 | **LRATD1** | 518 | **MBOAT2** | 696 | **ACAT2** | 874 | **ESYT3** |
| 163 | **F13B** | 341 | **ITGB1BP1** | 519 | **MBOAT1** | 697 | **TM6SF2** | 875 | **TM7SF2** |
| 164 | **F5** | 342 | **LCN6** | 520 | **PPARD** | 698 | **SOD1** | 876 | **DYRK1B** |
| 165 | **FAAP20** | 343 | **MSMO1** | 521 | **NR1H4** | 699 | **MMUT** | 877 | **ARV1** |
| 166 | **FANCM** | 344 | **CNTNAP2** | 522 | **RXRA** | 700 | **PTCH1** | 878 | **IMPK** |
| 167 | **FANCA** | 345 | **FANCB** | 523 | **CYP2D7** | 701 | **NFYA** | 879 | **SERPINA8** |
| 168 | **CRABP2** | 346 | **FAM53A** | 524 | **CYP2G1P** | 702 | **RELCH** | 880 | **CAGA** |
| 169 | **CENPS** | 347 | **FAM32A** | 525 | **PPARA** | 703 | **GPM6A** | 881 | **ANGPT5** |
| 170 | **KRAS** | 348 | **CNTNAP1** | 526 | **PLA2G2C** | 704 | **NFYB** | 882 | **LHR** |
| 171 | **FANCG** | 349 | **DDR2** | 527 | **CYP46A1** | 705 | **PIP4P1** | 883 | **FAS** |
| 172 | **BOD1** | 350 | **BOD1L1** | 528 | **LCAT** | 706 | **CD5L** | 884 | **COX1** |
| 173 | **FAM83F** | 351 | **ORM2** | 529 | **CYP7A1** | 707 | **LEP** | 885 | **LCN5** |
| 174 | **FAM83H** | 352 | **F8** | 530 | **CYP11A1** | 708 | **CYB5R3** | 886 | **GC** |
| 175 | **CERS5** | 353 | **FAM13A** | 531 | **SOAT1** | 709 | **SCP2** |  |  |
| 176 | **FANCC** | 354 | **FAM8A1** | 532 | **SOAT2** | 710 | **IHH** |  |  |
| 177 | **C19orf12** | 355 | **FAM107A** | 533 | **NPC1** | 711 | **LRP1** |  |  |
| 178 | **FANCE** | 356 | **FAM83G** | 534 | **ABCA1** | 712 | **CYB5R2** |  |  |
