# Supplementary material for: Network Pharmacology Identifies the Mechanisms of Sang-Xing-Zhi-Ke-Fang against Pharyngitis
Source: Evid Based Complement Alternat Med. 2020 Oct 12;2020:2421916. doi: 10.1155/2020/2421916 (PMC7576344; doi:10.1155/2020/2421916)
Supplement: Supplementary Materials — Table S1: 102 bioactive compounds obtained and screened out from TCMSP, BATMAN-TCM, and literature. Table S2: 886 targets of bioactive compounds collected using TCMSP and UniProt. Table S3: targets related to pharyngitis, including 5150 targets from CTD and 1803 targets from GeneCards with 695 targets duplicated. Table S4: 387 overlapping targets related to 19 bioactive compounds. Table S5: the results of topological features of the PPI network, including the values of topological features of 354 targets, while the other 33 targets were unrelated to each other target in the network. Table S6: the results of KEGG pathway enrichment, including 43 KEGG pathways were recognized as P < 0.05 with 28 pathways being recognized as P < 0.01. [file 2421916.f1.zip › Supplementary materials/Suppplementary Table S3.docx]

| **Names** | **total** | **elements** |
| --- | --- | --- |
| **CTD and GeneCard** | **695** | MMP2 |
|  |  | CD44 |
|  |  | ITGA5 |
|  |  | XDH |
|  |  | EDN1 |
|  |  | CXCR4 |
|  |  | GP1BA |
|  |  | HIC2 |
|  |  | SOD2 |
|  |  | FGR |
|  |  | COX15 |
|  |  | NR4A2 |
|  |  | EVC |
|  |  | LEP |
|  |  | WDR34 |
|  |  | PTPN22 |
|  |  | CYP2D6 |
|  |  | VKORC1 |
|  |  | UGT2B7 |
|  |  | HES1 |
|  |  | SELL |
|  |  | CCL19 |
|  |  | FOXE1 |
|  |  | GSTA2 |
|  |  | MAOA |
|  |  | APOE |
|  |  | RFXAP |
|  |  | HSPD1 |
|  |  | MZB1 |
|  |  | ADH5 |
|  |  | PREP |
|  |  | BCHE |
|  |  | BCL2 |
|  |  | DRD3 |
|  |  | ID1 |
|  |  | FUS |
|  |  | TF |
|  |  | DNAL4 |
|  |  | CEP120 |
|  |  | CYP1A1 |
|  |  | OFD1 |
|  |  | DBH |
|  |  | NEU1 |
|  |  | UNG |
|  |  | GSTP1 |
|  |  | CCR3 |
|  |  | ASAH1 |
|  |  | SPINK5 |
|  |  | HP |
|  |  | BTK |
|  |  | TBX21 |
|  |  | SOX9 |
|  |  | MIR452 |
|  |  | DLST |
|  |  | IRF7 |
|  |  | DNAJB4 |
|  |  | SHH |
|  |  | TNFRSF17 |
|  |  | TNFRSF1A |
|  |  | CASP1 |
|  |  | CD8A |
|  |  | XIAP |
|  |  | MAP4K3 |
|  |  | MICA |
|  |  | CD83 |
|  |  | NPY |
|  |  | IL18 |
|  |  | TAC1 |
|  |  | TYMS |
|  |  | SNCA |
|  |  | YPEL1 |
|  |  | HRAS |
|  |  | DEFA5 |
|  |  | ITGB2 |
|  |  | XRCC1 |
|  |  | VEGFA |
|  |  | GRIN2A |
|  |  | MYC |
|  |  | CCNA2 |
|  |  | ARID1B |
|  |  | NKX2-1 |
|  |  | GAL |
|  |  | SLC11A1 |
|  |  | MIR21 |
|  |  | KIF1B |
|  |  | TNFAIP3 |
|  |  | MMP1 |
|  |  | IL18RAP |
|  |  | HMBS |
|  |  | TRPV1 |
|  |  | MIR28 |
|  |  | FPR1 |
|  |  | ADGRG1 |
|  |  | GNLY |
|  |  | SMOC2 |
|  |  | PPARD |
|  |  | ANXA2 |
|  |  | FH |
|  |  | FOXP2 |
|  |  | GSC |
|  |  | MMP3 |
|  |  | TRAF6 |
|  |  | ERCC4 |
|  |  | MMP8 |
|  |  | CBX4 |
|  |  | NTRK2 |
|  |  | IL10 |
|  |  | CHD7 |
|  |  | TLR10 |
|  |  | MMP14 |
|  |  | PADI4 |
|  |  | MDM2 |
|  |  | TAP2 |
|  |  | EGFR |
|  |  | SOD1 |
|  |  | CYSLTR1 |
|  |  | PCNP |
|  |  | ERBB2 |
|  |  | CREB1 |
|  |  | UGT1A7 |
|  |  | LAT |
|  |  | NECTIN4 |
|  |  | MIR93 |
|  |  | G6PD |
|  |  | THBS1 |
|  |  | VIP |
|  |  | MC2R |
|  |  | MVK |
|  |  | HRH1 |
|  |  | CHEK2 |
|  |  | DRD4 |
|  |  | ADH4 |
|  |  | ID2 |
|  |  | METAP2 |
|  |  | HBB |
|  |  | HEY1 |
|  |  | KNG1 |
|  |  | SLAMF7 |
|  |  | PGR |
|  |  | MIR22 |
|  |  | CXCL10 |
|  |  | SLC1A3 |
|  |  | MIR140 |
|  |  | SAA4 |
|  |  | EHD1 |
|  |  | CDKN1A |
|  |  | MBP |
|  |  | DMD |
|  |  | SLURP1 |
|  |  | AIFM3 |
|  |  | G6PC |
|  |  | ADNP |
|  |  | POMC |
|  |  | LY9 |
|  |  | NLRP12 |
|  |  | NFKBIA |
|  |  | ALB |
|  |  | SUCLG2 |
|  |  | KISS1 |
|  |  | SERPINA3 |
|  |  | FABP2 |
|  |  | TWIST1 |
|  |  | EPO |
|  |  | IL1R1 |
|  |  | UBR4 |
|  |  | TYMP |
|  |  | MAP3K9 |
|  |  | YPEL2 |
|  |  | ACKR3 |
|  |  | PNP |
|  |  | MEF2C |
|  |  | LBR |
|  |  | IL2RG |
|  |  | PTGER3 |
|  |  | DNASE1 |
|  |  | IL12RB1 |
|  |  | MIR31 |
|  |  | MIR26B |
|  |  | COX5A |
|  |  | RAP1A |
|  |  | ID3 |
|  |  | MMP13 |
|  |  | HCP5 |
|  |  | IRF1 |
|  |  | IRF2BP2 |
|  |  | SOX4 |
|  |  | MSN |
|  |  | MECP2 |
|  |  | FCGR2B |
|  |  | LAMP1 |
|  |  | PRL |
|  |  | MAML2 |
|  |  | RAC2 |
|  |  | INTU |
|  |  | HTR2C |
|  |  | NEDD4 |
|  |  | ITGB4 |
|  |  | EZH2 |
|  |  | SLC25A1 |
|  |  | FAS |
|  |  | PON1 |
|  |  | MIR192 |
|  |  | CYSLTR2 |
|  |  | UGT2B15 |
|  |  | PTHLH |
|  |  | MIR27A |
|  |  | CD36 |
|  |  | GRK3 |
|  |  | CXCR2 |
|  |  | CD40 |
|  |  | CLDN2 |
|  |  | TAP1 |
|  |  | ETS2 |
|  |  | CS |
|  |  | FGB |
|  |  | FOLR1 |
|  |  | PRKCD |
|  |  | SERPINA1 |
|  |  | IL1RL1 |
|  |  | AHCY |
|  |  | EGR1 |
|  |  | IREB2 |
|  |  | SELE |
|  |  | ABCB1 |
|  |  | CNR1 |
|  |  | ITGB7 |
|  |  | PIK3C2A |
|  |  | FAAH |
|  |  | IL17A |
|  |  | TMPO |
|  |  | LEPR |
|  |  | GP6 |
|  |  | SATB2 |
|  |  | TH |
|  |  | SPOCK3 |
|  |  | SULT1A1 |
|  |  | ANK2 |
|  |  | VEGFC |
|  |  | MPO |
|  |  | ZBTB16 |
|  |  | PDGFB |
|  |  | ELN |
|  |  | VIM |
|  |  | MPI |
|  |  | HLA-A |
|  |  | LDHA |
|  |  | EDNRB |
|  |  | GRIN2B |
|  |  | STAT3 |
|  |  | ARSB |
|  |  | ITPR1 |
|  |  | ATXN3 |
|  |  | TGFBR1 |
|  |  | CDC42 |
|  |  | MIR24-1 |
|  |  | ATM |
|  |  | GSTT2 |
|  |  | GABRA2 |
|  |  | GRHL3 |
|  |  | UGT1A4 |
|  |  | CASP7 |
|  |  | UGT1A6 |
|  |  | F8 |
|  |  | ACHE |
|  |  | SIGMAR1 |
|  |  | MCL1 |
|  |  | PRNP |
|  |  | TLR3 |
|  |  | IL6 |
|  |  | IVD |
|  |  | MVP |
|  |  | HNRNPC |
|  |  | CASP3 |
|  |  | ACE |
|  |  | ITGAM |
|  |  | FASLG |
|  |  | MIR330 |
|  |  | PECAM1 |
|  |  | MS4A1 |
|  |  | CBLIF |
|  |  | TTR |
|  |  | CACNB4 |
|  |  | MAOB |
|  |  | BST2 |
|  |  | CYP1A2 |
|  |  | LYN |
|  |  | SERPINE2 |
|  |  | ADAM33 |
|  |  | ID4 |
|  |  | RNASE3 |
|  |  | PIK3CG |
|  |  | TPP1 |
|  |  | RREB1 |
|  |  | NQO1 |
|  |  | CD79A |
|  |  | SERPINB1 |
|  |  | PLAG1 |
|  |  | IFNG |
|  |  | FOS |
|  |  | SLC12A1 |
|  |  | MIR142 |
|  |  | FGF2 |
|  |  | PRODH |
|  |  | TOP2A |
|  |  | CAV1 |
|  |  | ALDH1A1 |
|  |  | SLC25A6 |
|  |  | CXCL1 |
|  |  | LDHC |
|  |  | MON2 |
|  |  | RASD1 |
|  |  | RELA |
|  |  | HIF1A |
|  |  | AQP1 |
|  |  | IL7R |
|  |  | TLR4 |
|  |  | MIR324 |
|  |  | CYP2C8 |
|  |  | MIR17 |
|  |  | HLA-DRB1 |
|  |  | TNFAIP2 |
|  |  | HLA-B |
|  |  | CANX |
|  |  | AKT1 |
|  |  | MUC5AC |
|  |  | LCN2 |
|  |  | SPARC |
|  |  | SERPINC1 |
|  |  | F3 |
|  |  | APOA1 |
|  |  | CDKN2A |
|  |  | PGD |
|  |  | GAD1 |
|  |  | NOTCH1 |
|  |  | PTPN13 |
|  |  | MIB1 |
|  |  | GSTT1 |
|  |  | ALDH9A1 |
|  |  | CD40LG |
|  |  | CYP3A4 |
|  |  | APP |
|  |  | PNKD |
|  |  | DLG1 |
|  |  | CYP26A1 |
|  |  | GLTP |
|  |  | RMRP |
|  |  | SLC52A1 |
|  |  | BTN2A1 |
|  |  | FBXO42 |
|  |  | MINDY1 |
|  |  | FGFR1 |
|  |  | BAK1 |
|  |  | GOT1 |
|  |  | HOXA1 |
|  |  | UGT1A1 |
|  |  | CSF3R |
|  |  | CYP2C19 |
|  |  | TLR1 |
|  |  | KCNA1 |
|  |  | DLX2 |
|  |  | MIR151A |
|  |  | TPI1 |
|  |  | ALDH2 |
|  |  | C3 |
|  |  | MIR99A |
|  |  | PTK2 |
|  |  | SERPING1 |
|  |  | TNFRSF10A |
|  |  | LDHB |
|  |  | BAMBI |
|  |  | SLAMF1 |
|  |  | LIF |
|  |  | ACTC1 |
|  |  | IL5 |
|  |  | BAGE |
|  |  | CRP |
|  |  | PNOC |
|  |  | KRT17 |
|  |  | DHFR |
|  |  | ALPP |
|  |  | PPP1R12A |
|  |  | ECE1 |
|  |  | HCK |
|  |  | PPL |
|  |  | SNAI1 |
|  |  | MIR485 |
|  |  | PLA2G6 |
|  |  | CTSB |
|  |  | TNF |
|  |  | NGFR |
|  |  | EGF |
|  |  | IL1A |
|  |  | ERCC1 |
|  |  | AGO2 |
|  |  | CREBBP |
|  |  | SELP |
|  |  | IFT43 |
|  |  | KIT |
|  |  | LRBA |
|  |  | PANK1 |
|  |  | SPP1 |
|  |  | F2 |
|  |  | GNS |
|  |  | PTGS2 |
|  |  | LRP5 |
|  |  | CD2 |
|  |  | CTNNB1 |
|  |  | ATXN7 |
|  |  | IL21R |
|  |  | CYP2C9 |
|  |  | GAD2 |
|  |  | CCND1 |
|  |  | BMP2 |
|  |  | ESR1 |
|  |  | SST |
|  |  | PRF1 |
|  |  | CDK4 |
|  |  | CD79B |
|  |  | KCNAB1 |
|  |  | BCL6 |
|  |  | FGFR4 |
|  |  | TGFB1 |
|  |  | GAST |
|  |  | UBE2L3 |
|  |  | DAG1 |
|  |  | TIMP1 |
|  |  | YES1 |
|  |  | IL13 |
|  |  | CD86 |
|  |  | GPT |
|  |  | STAT1 |
|  |  | TNFRSF25 |
|  |  | SLC2A1 |
|  |  | CCR7 |
|  |  | RGS1 |
|  |  | PRDM1 |
|  |  | SYP |
|  |  | PTPRC |
|  |  | HMGCR |
|  |  | MYBL1 |
|  |  | GGT1 |
|  |  | C1S |
|  |  | INS |
|  |  | FLT1 |
|  |  | CHRNA6 |
|  |  | FLCN |
|  |  | HDAC4 |
|  |  | GDNF |
|  |  | GSTM1 |
|  |  | CRH |
|  |  | MAPK1 |
|  |  | HNRNPA2B1 |
|  |  | PLAT |
|  |  | CXCR3 |
|  |  | APOH |
|  |  | IL4 |
|  |  | NAT2 |
|  |  | DYNLL1 |
|  |  | ACVR1 |
|  |  | DNAAF2 |
|  |  | NIF3L1 |
|  |  | MAP2K7 |
|  |  | GZMB |
|  |  | HLA-G |
|  |  | NTF3 |
|  |  | EIF4EBP1 |
|  |  | GUSB |
|  |  | TERT |
|  |  | RB1 |
|  |  | CD70 |
|  |  | IL1RN |
|  |  | KRT7 |
|  |  | MIR296 |
|  |  | SMTN |
|  |  | TNFRSF13B |
|  |  | MKI67 |
|  |  | CCL5 |
|  |  | TP53 |
|  |  | MX1 |
|  |  | LOX |
|  |  | CAT |
|  |  | ADM |
|  |  | IFIH1 |
|  |  | XRCC5 |
|  |  | RAB1A |
|  |  | NFKB1 |
|  |  | SIRT1 |
|  |  | ABCC1 |
|  |  | IL1B |
|  |  | HAX1 |
|  |  | FAM117B |
|  |  | MED19 |
|  |  | PAGR1 |
|  |  | HTR2A |
|  |  | KRT12 |
|  |  | APC |
|  |  | TLR2 |
|  |  | NAT1 |
|  |  | MMP10 |
|  |  | AUTS2 |
|  |  | PLAUR |
|  |  | BMP5 |
|  |  | DNAI2 |
|  |  | HNRNPA1 |
|  |  | ITIH4 |
|  |  | SLC17A5 |
|  |  | PDE4A |
|  |  | SOX2 |
|  |  | COMT |
|  |  | PDLIM5 |
|  |  | SLC6A3 |
|  |  | PVR |
|  |  | FANCG |
|  |  | DRD1 |
|  |  | RAC1 |
|  |  | EHF |
|  |  | PTEN |
|  |  | ANXA1 |
|  |  | SLC6A4 |
|  |  | NGF |
|  |  | ALOX5 |
|  |  | NLRP3 |
|  |  | RAB3GAP1 |
|  |  | TGM2 |
|  |  | DEFB1 |
|  |  | GRM8 |
|  |  | MTHFR |
|  |  | PTPN11 |
|  |  | ATF3 |
|  |  | LCT |
|  |  | DICER1 |
|  |  | DPP4 |
|  |  | NPPB |
|  |  | IKZF1 |
|  |  | APEH |
|  |  | LGALS1 |
|  |  | SRI |
|  |  | YWHAE |
|  |  | ATP1B1 |
|  |  | BDNF |
|  |  | FOXC1 |
|  |  | CSF1 |
|  |  | TNFSF10 |
|  |  | GPI |
|  |  | RPS27A |
|  |  | APOB |
|  |  | CFH |
|  |  | CDH2 |
|  |  | GTF3A |
|  |  | MIR137 |
|  |  | C4A |
|  |  | JAG1 |
|  |  | IRF6 |
|  |  | HSPA4 |
|  |  | RHOA |
|  |  | CALCA |
|  |  | GET4 |
|  |  | PPIG |
|  |  | MAPT |
|  |  | CXCL8 |
|  |  | TUBA1B |
|  |  | MTOR |
|  |  | WT1 |
|  |  | BNIP3 |
|  |  | AOC3 |
|  |  | CCN2 |
|  |  | VAPB |
|  |  | IGHM |
|  |  | SNAP25 |
|  |  | LCK |
|  |  | RAF1 |
|  |  | FURIN |
|  |  | SH3KBP1 |
|  |  | DRD2 |
|  |  | DES |
|  |  | FGFR2 |
|  |  | XRCC3 |
|  |  | CSF3 |
|  |  | PCNA |
|  |  | REV3L |
|  |  | CHUK |
|  |  | CCL20 |
|  |  | SLPI |
|  |  | PRPS1 |
|  |  | VTN |
|  |  | CYP2E1 |
|  |  | IL15 |
|  |  | LAT2 |
|  |  | CD46 |
|  |  | ICOSLG |
|  |  | ADRB2 |
|  |  | PRSS3 |
|  |  | CTSD |
|  |  | IL3 |
|  |  | PTCH1 |
|  |  | TFRC |
|  |  | KAT6A |
|  |  | GRIA3 |
|  |  | CHKA |
|  |  | CCL2 |
|  |  | MIR125A |
|  |  | EEF1A1 |
|  |  | HMGA2 |
|  |  | MIR15B |
|  |  | MIR185 |
|  |  | ITGA4 |
|  |  | STX3 |
|  |  | CD34 |
|  |  | KDR |
|  |  | SLC11A2 |
|  |  | RARA |
|  |  | TNC |
|  |  | AGBL3 |
|  |  | MIR145 |
|  |  | MIR30B |
|  |  | IL2RA |
|  |  | UGT1A9 |
|  |  | BTC |
|  |  | RAI1 |
|  |  | CD80 |
|  |  | HTR1B |
|  |  | RAP1B |
|  |  | GZMA |
|  |  | NFIB |
|  |  | MB |
|  |  | SNAP23 |
|  |  | FADD |
|  |  | IL2 |
|  |  | ALDH3A1 |
|  |  | SLC1A4 |
|  |  | CDH1 |
|  |  | EDA |
|  |  | CHRNA4 |
|  |  | TNFSF13B |
|  |  | SLC18A2 |
|  |  | H6PD |
|  |  | COL2A1 |
|  |  | MSX1 |
|  |  | WNT10A |
|  |  | MICB |
|  |  | EPHX1 |
|  |  | ICAM1 |
|  |  | CXCL9 |
|  |  | LGALS9 |
|  |  | MAPK3 |
|  |  | TNNI3K |
|  |  | MIR34C |
|  |  | VAMP3 |
|  |  | TCIRG1 |
|  |  | GRN |
|  |  | CYP51A1 |
|  |  | TGFA |
|  |  | MIR10B |
|  |  | DST |
|  |  | ZEB2 |
|  |  | JMJD1C |
|  |  | OPRM1 |
|  |  | SDHC |
|  |  | VCL |
|  |  | LTO1 |
|  |  | VCAM1 |
|  |  | STAT2 |
|  |  | REL |
|  |  | RAD51 |
|  |  | HMMR |
|  |  | ETV5 |
|  |  | GCG |
|  |  | CFB |
|  |  | CXCL6 |
|  |  | CCR5 |
|  |  | TXNRD3 |
|  |  | MIR425 |
|  |  | YAP1 |
|  |  | NNT |
|  |  | ITPKC |
|  |  | MIF |
|  |  | CPXCR1 |
|  |  | PTGS1 |
|  |  | CD69 |
|  |  | LTA |
|  |  | ETV6 |
|  |  | HGSNAT |
|  |  | LIG3 |
|  |  | DHODH |
|  |  | IL2RB |
|  |  | CD274 |
|  |  | BAX |
|  |  | CP |
|  |  | G6PC3 |
|  |  | VAV1 |
|  |  | TP63 |
|  |  | DPY19L1 |
|  |  | HCLS1 |
|  |  | CSF2 |
|  |  | MMP9 |
| **CTD** | **4455** | MSRB1 |
|  |  | PLPBP |
|  |  | RPS17 |
|  |  | SLMAP |
|  |  | HAUS2 |
|  |  | MIR194 |
|  |  | CMTR2 |
|  |  | TUBD1 |
|  |  | ERCC5 |
|  |  | GNL3 |
|  |  | FAM187A |
|  |  | BTG2 |
|  |  | NDUFS6 |
|  |  | GCSH |
|  |  | VPS4A |
|  |  | CYP11A1 |
|  |  | PFKP |
|  |  | AEN |
|  |  | CLK4 |
|  |  | KCNG1 |
|  |  | DECR1 |
|  |  | PTPRR |
|  |  | HYMAI |
|  |  | SLC25A3 |
|  |  | MTHFD2L |
|  |  | PXYLP1 |
|  |  | NHLRC3 |
|  |  | MIR20A |
|  |  | LMAN1 |
|  |  | LCORL |
|  |  | MIRLET7G |
|  |  | SUMO1 |
|  |  | STYK1 |
|  |  | SCAF8 |
|  |  | RPL19 |
|  |  | MMP7 |
|  |  | RTN1 |
|  |  | KLHL14 |
|  |  | NOS2 |
|  |  | CENPQ |
|  |  | TRIB3 |
|  |  | SLC2A5 |
|  |  | PPP1R3CB |
|  |  | DDX5 |
|  |  | REEP6 |
|  |  | CCNB1 |
|  |  | ZEB1 |
|  |  | CDK14 |
|  |  | MIR582 |
|  |  | TMEM42 |
|  |  | TMX4 |
|  |  | TUBB5 |
|  |  | CHD9 |
|  |  | AQP9 |
|  |  | CED-13 |
|  |  | LYAR |
|  |  | BANK1 |
|  |  | TRP53 |
|  |  | TNS3 |
|  |  | PIR |
|  |  | RWDD4A |
|  |  | SRPK1 |
|  |  | RRP7A |
|  |  | CDC42EP4A |
|  |  | MED13L |
|  |  | ZFYVE1 |
|  |  | MRPL1 |
|  |  | LY6K |
|  |  | CHD1 |
|  |  | NAT10 |
|  |  | DMC1 |
|  |  | SLC22A2 |
|  |  | RPS6KA5 |
|  |  | SUGCT |
|  |  | MAPKAPK2 |
|  |  | DHRS11 |
|  |  | CCL22 |
|  |  | CCDC34 |
|  |  | LHB |
|  |  | CMPK2 |
|  |  | BRCA1 |
|  |  | RABGGTA |
|  |  | GADL1 |
|  |  | STX8 |
|  |  | SLK |
|  |  | WDR89 |
|  |  | TIGAR |
|  |  | PRKCB |
|  |  | EIF4G3 |
|  |  | ACSM3 |
|  |  | ZNF623 |
|  |  | A2M |
|  |  | PHLDB3 |
|  |  | TRIQK |
|  |  | GRPEL1 |
|  |  | USP6NL |
|  |  | BAZ1B |
|  |  | SLCO2B1 |
|  |  | SIR-2.1 |
|  |  | ROCK2 |
|  |  | SNX16 |
|  |  | MIR331 |
|  |  | ANXA3 |
|  |  | BBS12 |
|  |  | PPP1R15B |
|  |  | TEK |
|  |  | TERF2IP |
|  |  | CD160 |
|  |  | EED |
|  |  | CPNE4 |
|  |  | RNF32 |
|  |  | COL1A1 |
|  |  | PDCD5 |
|  |  | AP1S2 |
|  |  | CLC |
|  |  | RAB33B |
|  |  | ZNF212 |
|  |  | MANEA |
|  |  | RT1-BB |
|  |  | ZNF83 |
|  |  | PDCD4 |
|  |  | CEP350 |
|  |  | SMYD3 |
|  |  | RAPGEF6 |
|  |  | NEIL1 |
|  |  | GPR55 |
|  |  | BORCS7 |
|  |  | STAU2 |
|  |  | SLFN12 |
|  |  | EXOC3 |
|  |  | VWA5A |
|  |  | STK17A |
|  |  | TNFRSF9 |
|  |  | KLK8 |
|  |  | CCNE1 |
|  |  | DUSP4 |
|  |  | PODN |
|  |  | SSU72 |
|  |  | SVA |
|  |  | UFM1 |
|  |  | USP14 |
|  |  | FAM174A |
|  |  | FBXO31 |
|  |  | FBXO11 |
|  |  | FRS2 |
|  |  | PSMG4 |
|  |  | GASK1B |
|  |  | RNF4 |
|  |  | NRARP |
|  |  | ING5 |
|  |  | ALG2 |
|  |  | PPP1R3D |
|  |  | GRB2 |
|  |  | TPM3 |
|  |  | ORM1 |
|  |  | EIF1 |
|  |  | CHST15 |
|  |  | LCOR |
|  |  | FPGT |
|  |  | N4BP2L2 |
|  |  | NTHL1 |
|  |  | B3GNT7 |
|  |  | ZNF232 |
|  |  | OPHN1 |
|  |  | SHBG |
|  |  | DESI1 |
|  |  | LYZL4 |
|  |  | CDIPT |
|  |  | GCC2 |
|  |  | HS2ST1 |
|  |  | RFX3 |
|  |  | CD82 |
|  |  | CRELD2 |
|  |  | ACY3.2 |
|  |  | KLHL30 |
|  |  | EIF1AX |
|  |  | GCLM |
|  |  | CPEB3 |
|  |  | PPM1A |
|  |  | SRSF11 |
|  |  | CAMTA1 |
|  |  | PGGHG |
|  |  | SAMSN1 |
|  |  | PTP4A2 |
|  |  | IL24 |
|  |  | PLCL2 |
|  |  | EMILIN1 |
|  |  | DUSP5 |
|  |  | THADA |
|  |  | NDUFS4 |
|  |  | GKN1 |
|  |  | PXDN |
|  |  | COX8A |
|  |  | A4GALT |
|  |  | SF3B6 |
|  |  | FAM110C |
|  |  | ZNF35 |
|  |  | XRN2 |
|  |  | CD6 |
|  |  | RSRC1 |
|  |  | ZSCAN4 |
|  |  | THRB |
|  |  | PWP2 |
|  |  | PSMD11 |
|  |  | NPR1 |
|  |  | ASS1 |
|  |  | APOBB.1 |
|  |  | USP42 |
|  |  | TMEM59 |
|  |  | SEC23IP |
|  |  | RGS4 |
|  |  | DDX18 |
|  |  | PTENP1 |
|  |  | RICTOR |
|  |  | SPTBN1 |
|  |  | IRS1 |
|  |  | CDC42EP3 |
|  |  | SRP72 |
|  |  | ATR |
|  |  | GNA11 |
|  |  | FSIP1 |
|  |  | CAPN1 |
|  |  | TRNN |
|  |  | GRIK2 |
|  |  | SNX25 |
|  |  | DPYD |
|  |  | TTLL4 |
|  |  | PPBP |
|  |  | SARS |
|  |  | KDM6A |
|  |  | QKI |
|  |  | ABHD12 |
|  |  | AGT |
|  |  | DAPP1 |
|  |  | PILRA |
|  |  | ABCF2 |
|  |  | CHRM1 |
|  |  | CHRNB2 |
|  |  | COL7A1 |
|  |  | NFIL3 |
|  |  | FUT4 |
|  |  | AL3A2 |
|  |  | UBAC2-AS1 |
|  |  | ECEL1 |
|  |  | C18ORF32 |
|  |  | FANCC |
|  |  | CENPU |
|  |  | HGH1 |
|  |  | GPATCH2 |
|  |  | ZNF227 |
|  |  | ARAP1 |
|  |  | KCNQ5 |
|  |  | AGPS |
|  |  | PLCXD2 |
|  |  | SELENOP |
|  |  | SELENOT1A |
|  |  | MED14 |
|  |  | SPRY2 |
|  |  | XPC |
|  |  | ARHGAP15 |
|  |  | ALDH1L2 |
|  |  | HBG2 |
|  |  | FAM218A |
|  |  | STAT5A |
|  |  | EIF4EBP3L |
|  |  | WASHC3 |
|  |  | FKBP4 |
|  |  | MIR122 |
|  |  | ZNF350 |
|  |  | TUBA3E |
|  |  | SDHB-1 |
|  |  | NFYA |
|  |  | PBLD |
|  |  | NADSYN1 |
|  |  | PIGF |
|  |  | PTGER1 |
|  |  | KIF14 |
|  |  | SMC6 |
|  |  | SMAD5-AS1 |
|  |  | PPP1R37 |
|  |  | AHR |
|  |  | MAD2L1 |
|  |  | ATP6V1C1 |
|  |  | IRX3 |
|  |  | RBM6 |
|  |  | WAPL |
|  |  | NFE2L2 |
|  |  | MAPK12A |
|  |  | NRF1 |
|  |  | SH2D2A |
|  |  | KLF4 |
|  |  | ACAD11 |
|  |  | RBBP6 |
|  |  | IFI27 |
|  |  | JADE1 |
|  |  | HDAC3 |
|  |  | KRCC1 |
|  |  | TJP3 |
|  |  | MT1G |
|  |  | SNORD26 |
|  |  | IDS |
|  |  | CLCA1 |
|  |  | OLFML2A |
|  |  | MICALL1 |
|  |  | GALR2 |
|  |  | ATP7B |
|  |  | NFYC |
|  |  | KIN |
|  |  | PPARGC1A |
|  |  | TNFAIP6 |
|  |  | CCT4 |
|  |  | PDAP1 |
|  |  | BRD4 |
|  |  | UPP2 |
|  |  | VMP1 |
|  |  | ZNF146 |
|  |  | CD151 |
|  |  | CYP19A1 |
|  |  | DOK4 |
|  |  | ARFRP1 |
|  |  | MANF |
|  |  | NPFFR2 |
|  |  | HRH2 |
|  |  | ENTPD7 |
|  |  | CYP3A5 |
|  |  | UBTD1 |
|  |  | CRB3A |
|  |  | CUL4B |
|  |  | ZNRD1 |
|  |  | CYP2C70 |
|  |  | TNRC18 |
|  |  | PITX1 |
|  |  | HADHAA |
|  |  | LCN15 |
|  |  | FBXO46 |
|  |  | JUND |
|  |  | ITSN1 |
|  |  | SVIL |
|  |  | OAS1 |
|  |  | PHB2 |
|  |  | ADCY3 |
|  |  | LIN28B |
|  |  | MRTO4 |
|  |  | TTC3 |
|  |  | MUC2 |
|  |  | CADPS2 |
|  |  | CYP2C93 |
|  |  | WNT6 |
|  |  | C5 |
|  |  | OSER1 |
|  |  | ATP2B2 |
|  |  | RGS20 |
|  |  | CMA1 |
|  |  | CENPL |
|  |  | FST |
|  |  | VWCE |
|  |  | LXN |
|  |  | ARHGEF26 |
|  |  | ZNF419 |
|  |  | TLK1 |
|  |  | TIGD2 |
|  |  | RELL1 |
|  |  | LDLR |
|  |  | ASTN2 |
|  |  | PROSER2 |
|  |  | KLF2 |
|  |  | GSDME |
|  |  | RPN1 |
|  |  | FAM160B1 |
|  |  | SIX2 |
|  |  | ND6 |
|  |  | TFE3 |
|  |  | PGM1 |
|  |  | POLR3G |
|  |  | KCTD13 |
|  |  | KLHL28 |
|  |  | DLGAP1 |
|  |  | TANK |
|  |  | LINC01527 |
|  |  | FAM193A |
|  |  | UGT1A8 |
|  |  | NTN4 |
|  |  | PECR |
|  |  | HSPA2 |
|  |  | FAM120A |
|  |  | SERP1 |
|  |  | ACAP3 |
|  |  | TYRP1 |
|  |  | LAMTOR3 |
|  |  | HIST1H3E |
|  |  | RAB20 |
|  |  | RBM7 |
|  |  | TARSL2 |
|  |  | JAK2 |
|  |  | POLR1A |
|  |  | TSHZ1 |
|  |  | ZNF264 |
|  |  | SIRPG |
|  |  | BICD1 |
|  |  | AGTR1A |
|  |  | SCARNA2 |
|  |  | TERF2 |
|  |  | USP9Y |
|  |  | ACADVL |
|  |  | ISCU |
|  |  | PLEKHM3 |
|  |  | XAF1 |
|  |  | MYCL |
|  |  | HBP1 |
|  |  | MIR17HG |
|  |  | PLA2G4C |
|  |  | XPO4 |
|  |  | HAUS4 |
|  |  | HCAR2 |
|  |  | SPAG6 |
|  |  | MKLN1 |
|  |  | HSD3B5 |
|  |  | TALDO1 |
|  |  | SUMF1 |
|  |  | B2M |
|  |  | OSM |
|  |  | IMPAD1 |
|  |  | MIRLET7E |
|  |  | SQLE |
|  |  | OPN3 |
|  |  | OLR1 |
|  |  | NRAP |
|  |  | SEPHS1 |
|  |  | PPFIBP1 |
|  |  | FAM49A |
|  |  | NCF1 |
|  |  | TRNL2 |
|  |  | ZNF614 |
|  |  | TCF19 |
|  |  | SAT2 |
|  |  | CBS |
|  |  | XXYLT1 |
|  |  | CLK3 |
|  |  | ATP5PO |
|  |  | PPID |
|  |  | UBE2J1 |
|  |  | ELF4 |
|  |  | AHRRB |
|  |  | NT5E |
|  |  | NCSTN |
|  |  | GK5 |
|  |  | SC5D |
|  |  | SULF2 |
|  |  | KLRG1 |
|  |  | NEURL3 |
|  |  | PSMD12 |
|  |  | TTC28-AS1 |
|  |  | HSCB |
|  |  | ANP32A |
|  |  | NPIPB7 |
|  |  | ACLY |
|  |  | CYP4F12 |
|  |  | NFAT5 |
|  |  | RRAGC |
|  |  | NUB1 |
|  |  | SRGAP2C |
|  |  | CCDC85B |
|  |  | WIF1 |
|  |  | GCH1 |
|  |  | SPDYE1 |
|  |  | RBM4.1 |
|  |  | FAM199X |
|  |  | PEF1 |
|  |  | SLC44A5 |
|  |  | C21ORF91 |
|  |  | MCM5 |
|  |  | ZFAS1 |
|  |  | ALG9 |
|  |  | CPA4 |
|  |  | BTBD10 |
|  |  | PTP4A3 |
|  |  | SPATS2L |
|  |  | NR1D2 |
|  |  | ARHGAP26 |
|  |  | TDRD9 |
|  |  | FBXL5 |
|  |  | LINC-PINT |
|  |  | BSDC1 |
|  |  | RABL6 |
|  |  | METTL16 |
|  |  | LCN5 |
|  |  | PHF12 |
|  |  | MAP2K4 |
|  |  | UGT5F1 |
|  |  | PHPT1 |
|  |  | EPB41L3 |
|  |  | CDKN2B |
|  |  | WSB1 |
|  |  | MIR361 |
|  |  | PROS1 |
|  |  | SOCS4 |
|  |  | PARVB |
|  |  | CSRNP1 |
|  |  | TBCCD1 |
|  |  | SETBP1 |
|  |  | NDC80 |
|  |  | PGBD3 |
|  |  | AIF1L |
|  |  | AREG |
|  |  | CDS2 |
|  |  | AHCTF1 |
|  |  | ITGA2 |
|  |  | LIPT1 |
|  |  | PCNX2 |
|  |  | EEF2K |
|  |  | MAST4 |
|  |  | ALP |
|  |  | SNHG32 |
|  |  | SNRNP48 |
|  |  | NR4A3 |
|  |  | ASIC3 |
|  |  | SPTLC3 |
|  |  | SOCS3 |
|  |  | C1ORF162 |
|  |  | SULT6B1 |
|  |  | LCE1E |
|  |  | CGRRF1 |
|  |  | ALPK1 |
|  |  | SPIC |
|  |  | MRPS18C |
|  |  | SPNS1 |
|  |  | TRAPPC6A |
|  |  | GATD3A |
|  |  | ZFP1 |
|  |  | E2F7 |
|  |  | LAMA3 |
|  |  | SCGB1D2 |
|  |  | RDH11 |
|  |  | SRSF3 |
|  |  | AUH |
|  |  | PDXP |
|  |  | RALB |
|  |  | GSK3B |
|  |  | MBD2 |
|  |  | ABHD14B |
|  |  | MOSMO |
|  |  | PSRC1 |
|  |  | ALAS1 |
|  |  | FOXO1 |
|  |  | KLHDC2 |
|  |  | CYP2AD2 |
|  |  | NDUFV2 |
|  |  | DUSP10 |
|  |  | PANK3 |
|  |  | RESF1 |
|  |  | FARS2 |
|  |  | PKP2 |
|  |  | CCDC28A |
|  |  | TMEM205 |
|  |  | PIAS1 |
|  |  | NPHP3 |
|  |  | ADCYAP1R1 |
|  |  | ZNF611 |
|  |  | NCOA6 |
|  |  | LRRFIP1 |
|  |  | GCLC |
|  |  | CNOT2 |
|  |  | HMGXB4 |
|  |  | MAN1A2 |
|  |  | SEC61A1 |
|  |  | FKTN |
|  |  | SLC35D1B |
|  |  | DDX50 |
|  |  | RPP30 |
|  |  | TRIM16 |
|  |  | BLVRB |
|  |  | GTPBP2 |
|  |  | GALNT10 |
|  |  | MIRLET7F |
|  |  | ELK4 |
|  |  | CD109 |
|  |  | ADCY1 |
|  |  | RHO |
|  |  | TP53I3 |
|  |  | SMURF2 |
|  |  | SRL |
|  |  | SLC22A3 |
|  |  | EBP |
|  |  | SH3PXD2A-AS1 |
|  |  | GPBP1 |
|  |  | MIR152 |
|  |  | GADD45B |
|  |  | TDP1 |
|  |  | RFK |
|  |  | ARHGAP8 |
|  |  | TRIM24 |
|  |  | TBC1D16 |
|  |  | DNAJB11 |
|  |  | SLCO1C1 |
|  |  | SLC22A5 |
|  |  | GRIN3A |
|  |  | DEPDC1 |
|  |  | KIF23 |
|  |  | USP38 |
|  |  | ARHGDIB |
|  |  | VNN3 |
|  |  | FSHB |
|  |  | URB2 |
|  |  | CYP2D9 |
|  |  | WTAPP1 |
|  |  | PDCD4A |
|  |  | HLCS |
|  |  | AK1 |
|  |  | GCSAM |
|  |  | STK10 |
|  |  | ABCD3 |
|  |  | TRNT |
|  |  | C4B |
|  |  | MTF2 |
|  |  | GSR |
|  |  | FAM20B |
|  |  | EGR4 |
|  |  | INTS7 |
|  |  | EIF2AK2 |
|  |  | CST4 |
|  |  | UCKL1 |
|  |  | LMO7 |
|  |  | PAM |
|  |  | IDO1 |
|  |  | ALDH1L1 |
|  |  | CFAP47 |
|  |  | MRM2 |
|  |  | THAP4 |
|  |  | ZNF84 |
|  |  | OGFRL1 |
|  |  | PPP1R15A |
|  |  | SLC39A4 |
|  |  | CITED2 |
|  |  | BMF |
|  |  | GCSHB |
|  |  | METTL18 |
|  |  | PYCR3 |
|  |  | FNDC4 |
|  |  | PCED1B |
|  |  | SIAH2 |
|  |  | NLGN4X |
|  |  | FBXO34 |
|  |  | CCDC103 |
|  |  | CPDA |
|  |  | EIF4E |
|  |  | ABHD5 |
|  |  | EXOC4 |
|  |  | TMBIM1 |
|  |  | MIR501 |
|  |  | ZDHHC1 |
|  |  | SLC35F5 |
|  |  | MIR590 |
|  |  | NRN1 |
|  |  | PCDHB14 |
|  |  | SLC37A4A |
|  |  | BRCA2 |
|  |  | ACADM |
|  |  | BBS9 |
|  |  | USP15 |
|  |  | TRNF |
|  |  | PTGER2 |
|  |  | SDSL |
|  |  | SLC31A1 |
|  |  | MIR34A |
|  |  | NPIPB15 |
|  |  | IRAK2 |
|  |  | GHITM |
|  |  | BLMH |
|  |  | MFGE8 |
|  |  | PFKFB2 |
|  |  | CLDND |
|  |  | WDR33 |
|  |  | TFB1M |
|  |  | GOLGB1 |
|  |  | CBX3A |
|  |  | OAZ1B |
|  |  | LRP1 |
|  |  | API5 |
|  |  | BIRC6 |
|  |  | THUMPD3 |
|  |  | POLR1B |
|  |  | RAD9B |
|  |  | SAMHD1 |
|  |  | PTBP3 |
|  |  | MIR532 |
|  |  | RPS4XP3 |
|  |  | EPC2 |
|  |  | C15 |
|  |  | SPRY1 |
|  |  | MYSM1 |
|  |  | TRIM4 |
|  |  | SERPINI1 |
|  |  | TMEM38A |
|  |  | PKD1P1 |
|  |  | NLK |
|  |  | CAPNS1 |
|  |  | FASTKD5 |
|  |  | SNHG15 |
|  |  | TDRD3 |
|  |  | EID2B |
|  |  | IPPK |
|  |  | SULT1ST3 |
|  |  | KLHL15 |
|  |  | AKT2 |
|  |  | VPS4B |
|  |  | SLC25A4 |
|  |  | CYB5B |
|  |  | SESN1 |
|  |  | FBXO9 |
|  |  | MRPL46 |
|  |  | SNX5 |
|  |  | EEF1D |
|  |  | BBOF1 |
|  |  | MAPKAPK3 |
|  |  | CHML |
|  |  | PAPOLG |
|  |  | MIR130A |
|  |  | CDK13 |
|  |  | CAPN2 |
|  |  | ZFP36L1 |
|  |  | TMX3 |
|  |  | SEC22C |
|  |  | SLC49A4 |
|  |  | RNF146 |
|  |  | TIAM2 |
|  |  | CHD2 |
|  |  | FZD5 |
|  |  | NR4A1 |
|  |  | TMEM175 |
|  |  | C16ORF74 |
|  |  | PURA |
|  |  | ZSCAN21 |
|  |  | RPL15 |
|  |  | PRC1 |
|  |  | ABCB9 |
|  |  | NAA38 |
|  |  | NEO1 |
|  |  | DDX3Y |
|  |  | MELTF |
|  |  | LGALS3 |
|  |  | CFL2 |
|  |  | RASA2 |
|  |  | FUT3 |
|  |  | NAP1L5 |
|  |  | DBI |
|  |  | PIF1 |
|  |  | ABCF1 |
|  |  | ZHX2 |
|  |  | PDP2 |
|  |  | FCF1 |
|  |  | APOOL |
|  |  | DIABLO |
|  |  | ZNF624 |
|  |  | LRP8 |
|  |  | ZNRF3 |
|  |  | ZNF211 |
|  |  | BBS4 |
|  |  | AKR1D1 |
|  |  | ZBED9 |
|  |  | STAT5B |
|  |  | HIST1H2BI |
|  |  | SSB |
|  |  | FKBP3 |
|  |  | ADGRG2 |
|  |  | TIMM23 |
|  |  | OAS2 |
|  |  | MIR224 |
|  |  | TMEM50B |
|  |  | MPP6 |
|  |  | TBL1XR1 |
|  |  | RPRD1A |
|  |  | APOLD1 |
|  |  | ANXA5B |
|  |  | GABRP |
|  |  | ATP1A1B |
|  |  | ZFP146 |
|  |  | PPP2R2A |
|  |  | HBE1 |
|  |  | ROBO4 |
|  |  | HBBP1 |
|  |  | CPEB4 |
|  |  | BCKDHA |
|  |  | RPL3 |
|  |  | SCLT1 |
|  |  | ABCC5 |
|  |  | EIF4ENIF1 |
|  |  | CMAS |
|  |  | TXNRD1 |
|  |  | SLC30A1 |
|  |  | ABCA11P |
|  |  | CES2E |
|  |  | SULT1D1 |
|  |  | SPATA18 |
|  |  | HPDL |
|  |  | CRIP2 |
|  |  | MBTD1 |
|  |  | CWC22 |
|  |  | FUT11 |
|  |  | RGS13 |
|  |  | PPP1CB |
|  |  | NRDC |
|  |  | DIO2 |
|  |  | ERBIN |
|  |  | MAP3K12 |
|  |  | UNC13A |
|  |  | TAF11 |
|  |  | CCDC136 |
|  |  | EKI1 |
|  |  | SLC3A2 |
|  |  | IER5 |
|  |  | MTHFD1L |
|  |  | HIST1H2AC |
|  |  | NCL |
|  |  | PER1B |
|  |  | TGS1 |
|  |  | MCM4 |
|  |  | SYF2 |
|  |  | ZBTB10 |
|  |  | FBXL17 |
|  |  | HTRA1 |
|  |  | LRMP |
|  |  | BHLHE40 |
|  |  | MCC |
|  |  | ATP2B4 |
|  |  | CHST12 |
|  |  | HHLA3 |
|  |  | RAB22A |
|  |  | TARDBP |
|  |  | P2RY10 |
|  |  | ARHGAP5 |
|  |  | ATXN1L |
|  |  | CHMP1A |
|  |  | ZNF160 |
|  |  | CETP |
|  |  | APOC2 |
|  |  | MTO1 |
|  |  | PLXNB2 |
|  |  | ILDR1 |
|  |  | GAS2 |
|  |  | PSA |
|  |  | NUMB |
|  |  | TNIP1 |
|  |  | INHBE |
|  |  | MAP3K4 |
|  |  | KTI12 |
|  |  | RHOG |
|  |  | UGT1B5 |
|  |  | TBC1D32 |
|  |  | MTA1 |
|  |  | CDK2 |
|  |  | NABP1 |
|  |  | GPAM |
|  |  | INTS13 |
|  |  | GNG7 |
|  |  | CDC37 |
|  |  | SRPX |
|  |  | SEC14L2 |
|  |  | POLR2A |
|  |  | CROCCP2 |
|  |  | ODC1 |
|  |  | ADORA2B |
|  |  | NR2F6 |
|  |  | TMEM218 |
|  |  | MIR19B |
|  |  | MSMO1 |
|  |  | ZFYVE16 |
|  |  | RPUSD2 |
|  |  | POLH |
|  |  | SYBU |
|  |  | THRA |
|  |  | MAPK11 |
|  |  | TBC1D22A |
|  |  | DDX17 |
|  |  | PLEKHA2 |
|  |  | ENO3 |
|  |  | GAS1 |
|  |  | PEG3 |
|  |  | ZNF559 |
|  |  | NADK |
|  |  | MED31 |
|  |  | GALT |
|  |  | ZMYM4 |
|  |  | ZNF532 |
|  |  | CAPSL |
|  |  | CEP-1 |
|  |  | ATP6AP2 |
|  |  | CRIP1 |
|  |  | AZIN1 |
|  |  | TP53TG1 |
|  |  | SLC7A7 |
|  |  | IL10RA |
|  |  | IL36RN |
|  |  | FBLN7 |
|  |  | CDC42EP4 |
|  |  | UBE2A |
|  |  | ELA3L |
|  |  | ARG2 |
|  |  | UBE2CBP |
|  |  | NOL7 |
|  |  | BRMS1L |
|  |  | TSLP |
|  |  | NHS |
|  |  | ICAM3 |
|  |  | IGLC2 |
|  |  | TM9SF3 |
|  |  | DDHD1 |
|  |  | RBM41 |
|  |  | HSPA7 |
|  |  | PTTG3P |
|  |  | MT1F |
|  |  | ALKBH5 |
|  |  | RPL35A |
|  |  | RAB40C |
|  |  | CBX3 |
|  |  | ZCCHC2 |
|  |  | CLIP3 |
|  |  | CHIC1 |
|  |  | ACAP2 |
|  |  | CARNMT1 |
|  |  | SULF1 |
|  |  | PPP4R2 |
|  |  | KIF3A |
|  |  | EFL-2 |
|  |  | CDCA2 |
|  |  | DTD2 |
|  |  | DCXR |
|  |  | PDIA6 |
|  |  | PBXIP1 |
|  |  | SIGLEC5 |
|  |  | ZMYM2 |
|  |  | TAB3 |
|  |  | LRRN4CL |
|  |  | MARCKSL1 |
|  |  | NBEAP1 |
|  |  | ZNF550 |
|  |  | MTERF3 |
|  |  | FBXL3 |
|  |  | FTCDNL1 |
|  |  | KIZ |
|  |  | CLEC2D |
|  |  | LSR |
|  |  | DIO1 |
|  |  | LIPC |
|  |  | HIST1H3D |
|  |  | SLC6A12 |
|  |  | RASA1 |
|  |  | ZNF567 |
|  |  | PATJ |
|  |  | LGALS3BPA |
|  |  | NREP |
|  |  | GJC1 |
|  |  | TANGO6 |
|  |  | SLC2A2 |
|  |  | GABRA5 |
|  |  | CELSR2 |
|  |  | C15ORF39 |
|  |  | PTPMT1 |
|  |  | PIK3R1 |
|  |  | MAF |
|  |  | PDGFRA |
|  |  | CACNG1 |
|  |  | ZMIZ2 |
|  |  | CSTB |
|  |  | HILPDA |
|  |  | STAMBP |
|  |  | TBC1D27P |
|  |  | NDUFA12 |
|  |  | KRTAP2-4 |
|  |  | DPL-1 |
|  |  | CENPI |
|  |  | STAMBPL1 |
|  |  | HOOK1 |
|  |  | GTF2E2 |
|  |  | FPR2 |
|  |  | CCNF |
|  |  | NFE2L2A |
|  |  | OTC |
|  |  | RPP25L |
|  |  | PPM1G |
|  |  | ABCB11 |
|  |  | SPACA9 |
|  |  | RPR |
|  |  | NMT1 |
|  |  | SOCS5 |
|  |  | MRPL58 |
|  |  | WFDC12 |
|  |  | TAAR1 |
|  |  | DLG5 |
|  |  | PLEKHF1 |
|  |  | PER1 |
|  |  | MUSTN1 |
|  |  | COL26A1 |
|  |  | RND2 |
|  |  | HDAC1 |
|  |  | NRSN2-AS1 |
|  |  | ATG12 |
|  |  | FRG1BP |
|  |  | SH3RF1 |
|  |  | GPR63 |
|  |  | BLZF1 |
|  |  | ZHX1 |
|  |  | SARAF |
|  |  | FOSB |
|  |  | SLC27A2 |
|  |  | MED26 |
|  |  | NDUFB2 |
|  |  | NEURL1B |
|  |  | WARS2 |
|  |  | DDO |
|  |  | SLC25A45 |
|  |  | GNB1 |
|  |  | UPF3B |
|  |  | PIDD1 |
|  |  | UST |
|  |  | C9ORF16 |
|  |  | LCAT |
|  |  | CALD1 |
|  |  | ZFP36L1A |
|  |  | AIG1 |
|  |  | ST6GAL1 |
|  |  | APOA4 |
|  |  | RHOAB |
|  |  | PMS1 |
|  |  | CMET |
|  |  | MUC3 |
|  |  | TMED6 |
|  |  | RPL31 |
|  |  | HMGCS1 |
|  |  | KDELR2 |
|  |  | TLE4 |
|  |  | C2ORF69 |
|  |  | TLK2 |
|  |  | MARS2 |
|  |  | SDF2L1 |
|  |  | ANO3 |
|  |  | HIBADH |
|  |  | IGFBP3 |
|  |  | DEK |
|  |  | PSPC1 |
|  |  | GRAMD2B |
|  |  | ARHGAP24 |
|  |  | ZNF823 |
|  |  | CPT1A |
|  |  | ALMS1 |
|  |  | PRDX-3 |
|  |  | EIF3I |
|  |  | ARRDC3A |
|  |  | NINJ1 |
|  |  | SFXN4 |
|  |  | CDKN2AIPNL |
|  |  | SPRED2 |
|  |  | BBIP1 |
|  |  | CYB5A |
|  |  | N4BP2L1 |
|  |  | GBP5 |
|  |  | PITPNC1 |
|  |  | KCTD3 |
|  |  | LONRF1 |
|  |  | CCP110 |
|  |  | CACNG6 |
|  |  | CEP128 |
|  |  | PLP1 |
|  |  | PIK3R6 |
|  |  | ZNF804A |
|  |  | IFRD2 |
|  |  | KCNMB4 |
|  |  | NARS2 |
|  |  | STX6 |
|  |  | TSFM |
|  |  | MTX2 |
|  |  | BRD3 |
|  |  | BNC2 |
|  |  | WDR3 |
|  |  | PCOLCE2 |
|  |  | IRF9 |
|  |  | MCPH1 |
|  |  | FTL1 |
|  |  | ZNF57 |
|  |  | FBXL4 |
|  |  | ROR1 |
|  |  | MYO1D |
|  |  | BMP2K |
|  |  | FAM13B |
|  |  | HINT1 |
|  |  | WNT7B |
|  |  | HNRNPU |
|  |  | FKBP11 |
|  |  | RHOBTB1 |
|  |  | FLII |
|  |  | ELA2 |
|  |  | KAZN |
|  |  | RPS16 |
|  |  | ZNF675 |
|  |  | SMAP2 |
|  |  | CSNK2A1 |
|  |  | RFC5 |
|  |  | BRAF |
|  |  | BABAM2 |
|  |  | NEMP1 |
|  |  | MIR20B |
|  |  | AAK1 |
|  |  | COL1A2 |
|  |  | MAFF |
|  |  | AKT3 |
|  |  | LY96 |
|  |  | GEMIN5 |
|  |  | DDX20 |
|  |  | FABP9 |
|  |  | UTP15 |
|  |  | PROCR |
|  |  | ARL5A |
|  |  | PHF11 |
|  |  | PHKB |
|  |  | TBC1D8B |
|  |  | ZNF776 |
|  |  | RBAK |
|  |  | EHHADH |
|  |  | KMT2C |
|  |  | YIPF3 |
|  |  | SI |
|  |  | NR1D1 |
|  |  | APOC1 |
|  |  | RIC-3 |
|  |  | ARID1A |
|  |  | PARD6B |
|  |  | RBM48 |
|  |  | HIST2H2BE |
|  |  | FLVCR2 |
|  |  | ALPL |
|  |  | CHST3 |
|  |  | SAT1 |
|  |  | RPP38 |
|  |  | ZNF280D |
|  |  | CYP4A10 |
|  |  | UQCRC2 |
|  |  | RYBP |
|  |  | NCR3LG1 |
|  |  | KLRB1 |
|  |  | PSD3 |
|  |  | ALPK2 |
|  |  | CPSF2 |
|  |  | NLRC3 |
|  |  | CYP4A8 |
|  |  | ZADH2 |
|  |  | FANCD2 |
|  |  | HMGB1 |
|  |  | RTL10 |
|  |  | ZNF304 |
|  |  | PSPH |
|  |  | MFSD3 |
|  |  | CDIP1 |
|  |  | RAP1GDS1 |
|  |  | CCDC51 |
|  |  | KLF3 |
|  |  | CDC25A |
|  |  | ANKRD37 |
|  |  | BAIAP2L1 |
|  |  | ESM1 |
|  |  | B3GNT5 |
|  |  | ERGIC1 |
|  |  | EML2-AS1 |
|  |  | KANK1 |
|  |  | MAP4 |
|  |  | ACTR3 |
|  |  | ADH1 |
|  |  | ZNF485 |
|  |  | PTPRCAP |
|  |  | HIVEP1 |
|  |  | HECA |
|  |  | ATRX |
|  |  | EIF4EBP2 |
|  |  | TNIK |
|  |  | CCNA1 |
|  |  | THEM6 |
|  |  | BID |
|  |  | SOX7 |
|  |  | ARV1 |
|  |  | IER2 |
|  |  | HMGB1B |
|  |  | AIFM1 |
|  |  | SEC24D |
|  |  | POLR2L |
|  |  | MDFIC |
|  |  | NUFIP2 |
|  |  | ZDHHC2 |
|  |  | PXT1 |
|  |  | ATP6V0D1 |
|  |  | ACR-8 |
|  |  | MEX3B |
|  |  | POLR1C |
|  |  | KANSL2 |
|  |  | NID1 |
|  |  | NUBP2 |
|  |  | DEPDC1B |
|  |  | ZNF85 |
|  |  | ZNF473 |
|  |  | MIR33A |
|  |  | CLGN |
|  |  | TFDP2 |
|  |  | PADI3 |
|  |  | CCDC12 |
|  |  | AMBRA1 |
|  |  | ZNF764 |
|  |  | ND1 |
|  |  | SPATA5L1 |
|  |  | RAD51B |
|  |  | EBI2 |
|  |  | TPD52L2 |
|  |  | PTPRE |
|  |  | UNC5B |
|  |  | ZSCAN5A |
|  |  | PLAAT4 |
|  |  | ZNF230 |
|  |  | TSPAN3 |
|  |  | SERINC3 |
|  |  | EPDR1 |
|  |  | CYP1A3 |
|  |  | MT1 |
|  |  | TPP2 |
|  |  | GTF2E1 |
|  |  | ST6GALNAC5 |
|  |  | ATAD2B |
|  |  | CHSY1 |
|  |  | GPNMB |
|  |  | KCNIP3 |
|  |  | SFPQ |
|  |  | AKR1B10 |
|  |  | RGS16 |
|  |  | TCIM |
|  |  | CLN8 |
|  |  | GPR137B |
|  |  | MAP3K5 |
|  |  | RXRA |
|  |  | TOP3B |
|  |  | PDLIM7 |
|  |  | NOS1 |
|  |  | ABITRAM |
|  |  | ARL14 |
|  |  | TIMP3 |
|  |  | FUBP3 |
|  |  | ACACA |
|  |  | ABCG1 |
|  |  | C16ORF70 |
|  |  | MIR98 |
|  |  | NFATC1 |
|  |  | ABHD4 |
|  |  | CD55 |
|  |  | UAP1L1 |
|  |  | LNPEP |
|  |  | SMAD3 |
|  |  | TBC1D12 |
|  |  | FBXO22 |
|  |  | PTGDR |
|  |  | THAP10 |
|  |  | ELOVL3 |
|  |  | PLCG1 |
|  |  | SLC16A7 |
|  |  | CHMP5 |
|  |  | ZNF43 |
|  |  | MDH1 |
|  |  | MRPL19 |
|  |  | ZRANB3 |
|  |  | RBM33 |
|  |  | ATP6V0B |
|  |  | YTHDF3 |
|  |  | SEPTIN6 |
|  |  | MIR19A |
|  |  | LCE1B |
|  |  | ANGPTL3 |
|  |  | MYO6 |
|  |  | DNAJA4 |
|  |  | ATP11A |
|  |  | AKIRIN2 |
|  |  | SLC25A5 |
|  |  | LINC00941 |
|  |  | CFAP73 |
|  |  | PELO |
|  |  | RNASEK |
|  |  | SNORA21 |
|  |  | BTN3A2 |
|  |  | CNKSR3 |
|  |  | HSPBAP1 |
|  |  | BORA |
|  |  | HBEGF |
|  |  | OSMR |
|  |  | SOD3 |
|  |  | DRAXIN |
|  |  | RASAL1 |
|  |  | DDAH1 |
|  |  | TIPARP |
|  |  | SAMD13 |
|  |  | SPRYD4 |
|  |  | DNASE1L3 |
|  |  | RIOX2 |
|  |  | CRYAB |
|  |  | MIER3 |
|  |  | CHEK1 |
|  |  | SCRN2 |
|  |  | S22A2 |
|  |  | COPA |
|  |  | SKP2 |
|  |  | TNFRSF11B |
|  |  | COA6 |
|  |  | CCT2 |
|  |  | ARF4 |
|  |  | MIR10A |
|  |  | ANAPC13 |
|  |  | CLK2 |
|  |  | LIN-35 |
|  |  | DUSP2 |
|  |  | MIR215 |
|  |  | NCF2 |
|  |  | KBTBD6 |
|  |  | MRPL48 |
|  |  | URI1 |
|  |  | PAG1 |
|  |  | PIK3C3 |
|  |  | SOS2 |
|  |  | TNNC2 |
|  |  | GPCPD1 |
|  |  | ANKRD28 |
|  |  | ZBTB2 |
|  |  | CDK2AP2 |
|  |  | C5ORF30 |
|  |  | ALG14 |
|  |  | B4GALT1 |
|  |  | LIPE |
|  |  | KNSTRN |
|  |  | C12ORF45 |
|  |  | SLC25A44 |
|  |  | MIRLET7C |
|  |  | ZSCAN26 |
|  |  | MIR181B1 |
|  |  | FGD4 |
|  |  | RNF145 |
|  |  | LY86 |
|  |  | EFCAB7 |
|  |  | GDI1 |
|  |  | ZMYND8 |
|  |  | CCDC186 |
|  |  | PDXK |
|  |  | GSAP |
|  |  | HSPB11 |
|  |  | MIR23A |
|  |  | CYP11B2 |
|  |  | ZNF670 |
|  |  | N4BP2 |
|  |  | MIR181D |
|  |  | IFFO2 |
|  |  | MED10 |
|  |  | SIRT4 |
|  |  | PRDX1 |
|  |  | NACC2 |
|  |  | EHMT2 |
|  |  | COX17 |
|  |  | RSKR |
|  |  | ECE2 |
|  |  | TSGA10 |
|  |  | TCF12 |
|  |  | ZNF721 |
|  |  | PHF23A |
|  |  | UNC-68 |
|  |  | SCD5 |
|  |  | ZNF641 |
|  |  | MAL |
|  |  | PRRG4 |
|  |  | NCEH1 |
|  |  | WDR5B |
|  |  | AQP4 |
|  |  | BAG1 |
|  |  | ARMC8 |
|  |  | MYNN |
|  |  | SLC47A1 |
|  |  | DCUN1D3 |
|  |  | TBXA2R |
|  |  | PAN2 |
|  |  | C12ORF54 |
|  |  | NUPR1 |
|  |  | SMC1A |
|  |  | DCLRE1B |
|  |  | ITGA8 |
|  |  | LPXN |
|  |  | TMPRSS6 |
|  |  | FAM27D1 |
|  |  | MIR125B-2 |
|  |  | TP53INP2 |
|  |  | OVAL |
|  |  | PUS1 |
|  |  | PRKAA1 |
|  |  | C11ORF54 |
|  |  | CYP4F3 |
|  |  | RASEF |
|  |  | DBF4 |
|  |  | POGK |
|  |  | CROT |
|  |  | MAPK9 |
|  |  | THSD1 |
|  |  | PPTC7 |
|  |  | ESRRG |
|  |  | RIPK2 |
|  |  | CLDN7 |
|  |  | STON2 |
|  |  | TRIM37 |
|  |  | STOX2 |
|  |  | GRSF1 |
|  |  | PSMC6 |
|  |  | ABCC4 |
|  |  | FAR2 |
|  |  | SSR3 |
|  |  | NAA16 |
|  |  | PPP4R3B |
|  |  | DYRK2 |
|  |  | KLF6 |
|  |  | MTF1 |
|  |  | THBD |
|  |  | TRNS1 |
|  |  | SRR |
|  |  | TM2D3 |
|  |  | SLC32A1 |
|  |  | ARNTL2 |
|  |  | DCD |
|  |  | ASPH |
|  |  | PVRIG |
|  |  | FLOT2 |
|  |  | OXCT1 |
|  |  | PKD1L2 |
|  |  | PRH2 |
|  |  | CDKN2D |
|  |  | ABCA8 |
|  |  | PGBD1 |
|  |  | PRPF4B |
|  |  | LSMEM1 |
|  |  | ILK |
|  |  | ZNF20 |
|  |  | SLC22A8 |
|  |  | KCNQ1OT1 |
|  |  | MAPK14 |
|  |  | SERPINB2 |
|  |  | CRABP1A |
|  |  | PRR14L |
|  |  | FABP3 |
|  |  | PLA1A |
|  |  | FBXW12 |
|  |  | RNF113A |
|  |  | E75 |
|  |  | ABTB2 |
|  |  | TSPYL2 |
|  |  | EIF2S3 |
|  |  | MORC3 |
|  |  | FZD7 |
|  |  | CHL1 |
|  |  | VANGL2 |
|  |  | MAP3K8 |
|  |  | AKAP10 |
|  |  | STC1 |
|  |  | STOM |
|  |  | SLC13A2 |
|  |  | GSN |
|  |  | EGR2 |
|  |  | SMARCA5 |
|  |  | WWC1 |
|  |  | FRMD6 |
|  |  | RAB23 |
|  |  | SH3GL3 |
|  |  | PLA2G4A |
|  |  | ARPP19 |
|  |  | CYP1A |
|  |  | SENP6 |
|  |  | RIF1 |
|  |  | MIR100HG |
|  |  | ST20-AS1 |
|  |  | VLDLR |
|  |  | MAP2K2 |
|  |  | DBT |
|  |  | DLC |
|  |  | CGGBP1 |
|  |  | HIST1H2BB |
|  |  | RNF19B |
|  |  | KLK2 |
|  |  | UQCRB |
|  |  | SUMO3 |
|  |  | FAM102BB |
|  |  | NAA50 |
|  |  | ABCC3 |
|  |  | SMIM7 |
|  |  | HLA-DMB |
|  |  | SAMD9L |
|  |  | CARS2 |
|  |  | KIF21A |
|  |  | ARHGEF3 |
|  |  | EXOC8 |
|  |  | CPN1 |
|  |  | SUPT3H |
|  |  | ADAMTS4 |
|  |  | MAML3 |
|  |  | MIR99B |
|  |  | SLIT3 |
|  |  | CAR3 |
|  |  | RHEBL1 |
|  |  | EGLN1 |
|  |  | SCD |
|  |  | MIR106B |
|  |  | HSPA6 |
|  |  | DYDC2 |
|  |  | NT5DC4 |
|  |  | ARHGEF39 |
|  |  | LIN7A |
|  |  | PHC2 |
|  |  | SLC38A2 |
|  |  | CAPG |
|  |  | WDR36 |
|  |  | TPCN2 |
|  |  | TRIM26 |
|  |  | CA5BP1 |
|  |  | MPZL1 |
|  |  | ZNF578 |
|  |  | PARPBP |
|  |  | RUVBL1 |
|  |  | MAD1L1 |
|  |  | SPIN4 |
|  |  | APAF1 |
|  |  | ZNF296 |
|  |  | INPP1 |
|  |  | NOP16 |
|  |  | DPEP3 |
|  |  | NDUFA4 |
|  |  | TBC1D20 |
|  |  | PKP4 |
|  |  | DUSP1 |
|  |  | SEZ6L2 |
|  |  | DDAH2 |
|  |  | VPS11 |
|  |  | CSTAD |
|  |  | NDUFB5 |
|  |  | TRIM27 |
|  |  | ADO |
|  |  | CIDEA |
|  |  | CDC42EP2 |
|  |  | HSP70L |
|  |  | RABGAP1 |
|  |  | HLFA |
|  |  | IER5L |
|  |  | N6AMT1 |
|  |  | H19 |
|  |  | MIR450A1 |
|  |  | MIR16 |
|  |  | ATG4D |
|  |  | GBP2 |
|  |  | WDR4 |
|  |  | VPS33B |
|  |  | LRPPRC |
|  |  | NDUFAF7 |
|  |  | SETD5 |
|  |  | MRPL35 |
|  |  | RRS1 |
|  |  | SLC35G1 |
|  |  | SNX2 |
|  |  | ABI3BP |
|  |  | TSNAX |
|  |  | SEMA7A |
|  |  | MADD |
|  |  | TIE1 |
|  |  | PCID2 |
|  |  | CATSPERD |
|  |  | EDF1 |
|  |  | CRYBG2 |
|  |  | KLF12 |
|  |  | EZRB |
|  |  | HSPA1A |
|  |  | TINAG |
|  |  | RUNDC3B |
|  |  | IL17RB |
|  |  | CENPA |
|  |  | MT2 |
|  |  | TMED5 |
|  |  | MCAM |
|  |  | CDH6 |
|  |  | POLR3E |
|  |  | TFF2 |
|  |  | CDCA7 |
|  |  | MINDY3 |
|  |  | MAP7D2 |
|  |  | USP45 |
|  |  | RIMKLB |
|  |  | ZNF134 |
|  |  | PPIB |
|  |  | PCGF2 |
|  |  | SFN |
|  |  | MTERF1 |
|  |  | MTREX |
|  |  | SSTR3 |
|  |  | PLCD4 |
|  |  | MDM1 |
|  |  | CD9 |
|  |  | RBL2 |
|  |  | ALDH7A1 |
|  |  | H2-T18 |
|  |  | SNHG7 |
|  |  | USP53 |
|  |  | CDC20 |
|  |  | TMED2 |
|  |  | E2F5 |
|  |  | ACSL5 |
|  |  | IGFBP1A |
|  |  | AFM |
|  |  | G0S2 |
|  |  | CENPC |
|  |  | FCHSD2 |
|  |  | SLC4A7 |
|  |  | PFKM |
|  |  | STAT6 |
|  |  | SGMS1 |
|  |  | ZNF195 |
|  |  | EFL-1 |
|  |  | GART |
|  |  | CREBZF |
|  |  | C17ORF99 |
|  |  | RIOK3 |
|  |  | PER3 |
|  |  | TRIAP1 |
|  |  | SCARB1 |
|  |  | ARMC10 |
|  |  | GCHFR |
|  |  | UBD |
|  |  | ITPR2 |
|  |  | SUN2 |
|  |  | UTP23 |
|  |  | MIGA1 |
|  |  | GPI1 |
|  |  | RPL23AP7 |
|  |  | MT1X |
|  |  | UBE2B |
|  |  | NUMBL |
|  |  | MUC4 |
|  |  | OMA1 |
|  |  | H2AFX |
|  |  | JAZF1 |
|  |  | BTG4 |
|  |  | GNB2 |
|  |  | PLOD3 |
|  |  | HAVCR1 |
|  |  | MFSD1 |
|  |  | LRP10 |
|  |  | TRAPPC10 |
|  |  | KRI1 |
|  |  | PUS7L |
|  |  | PLEKHF2 |
|  |  | PRXL2A |
|  |  | B3GLCT |
|  |  | BUB1 |
|  |  | HID |
|  |  | FAM173A |
|  |  | DIMT1 |
|  |  | MR1 |
|  |  | SURF4L |
|  |  | LASP1 |
|  |  | TCAIM |
|  |  | TNFRSF12A |
|  |  | SLC28A1 |
|  |  | EGR3 |
|  |  | OASL |
|  |  | SEH1L |
|  |  | ZNF184 |
|  |  | IFRD1 |
|  |  | WDFY1 |
|  |  | PRR14 |
|  |  | ZNF267 |
|  |  | ZBTB6 |
|  |  | EHBP1 |
|  |  | PRR11 |
|  |  | TXNIP |
|  |  | PDIK1L |
|  |  | NUDT3 |
|  |  | POPDC2 |
|  |  | C1ORF158 |
|  |  | TRIP13 |
|  |  | ERG28 |
|  |  | RAP2B |
|  |  | CPED1 |
|  |  | MIR520F |
|  |  | SRSF8 |
|  |  | KIF3B |
|  |  | BRD1 |
|  |  | PRICKLE2 |
|  |  | CDC7 |
|  |  | MCCC2 |
|  |  | SNX7 |
|  |  | CPT2 |
|  |  | HEATR1 |
|  |  | SPRNP1 |
|  |  | NSDHL |
|  |  | GLMP |
|  |  | UGT2A1 |
|  |  | KANSL1L |
|  |  | LSS |
|  |  | EPOR |
|  |  | ARIH2OS |
|  |  | CXCL15 |
|  |  | ISG15 |
|  |  | DHRS9 |
|  |  | SUGT1P3 |
|  |  | ZCCHC14 |
|  |  | CREBL2 |
|  |  | ZBTB14 |
|  |  | BCAS4 |
|  |  | FAM200B |
|  |  | PPAN |
|  |  | SCLY |
|  |  | LINC01588 |
|  |  | CA7 |
|  |  | RNF213 |
|  |  | CNEP1R1 |
|  |  | TMEM67 |
|  |  | CLK1 |
|  |  | WDR47 |
|  |  | KCTD20 |
|  |  | HSD3B7 |
|  |  | VDR |
|  |  | PRPF40A |
|  |  | C4ORF47 |
|  |  | CLDN12 |
|  |  | THAP2 |
|  |  | PRKCE |
|  |  | DTD1 |
|  |  | INTS11 |
|  |  | DUSP11 |
|  |  | RGS2 |
|  |  | TIGD7 |
|  |  | GFRA3 |
|  |  | COL3A1 |
|  |  | RTN4 |
|  |  | MIR126 |
|  |  | GFM2 |
|  |  | GNL3L |
|  |  | NDUFB10 |
|  |  | PRKAA2 |
|  |  | IVNS1ABP |
|  |  | TRNV |
|  |  | SGPP2 |
|  |  | DUSP19 |
|  |  | DNAJC14 |
|  |  | PPP1R2 |
|  |  | FOSL2 |
|  |  | ABCG2 |
|  |  | PGAM5 |
|  |  | TUBA4A |
|  |  | GRB10 |
|  |  | EPPK1 |
|  |  | EEF2 |
|  |  | TBL1X |
|  |  | EPHA3 |
|  |  | ZDHHC11 |
|  |  | HNF1A |
|  |  | PRDX3 |
|  |  | MUC19 |
|  |  | RBM12 |
|  |  | H2AFJ |
|  |  | LRIF1 |
|  |  | EDIL3 |
|  |  | INSIG2 |
|  |  | MIR148B |
|  |  | SERGEF |
|  |  | OSCAR |
|  |  | ATP5F1E |
|  |  | LATS2 |
|  |  | PEX2 |
|  |  | LEAP2 |
|  |  | GADD45G |
|  |  | TMEM51 |
|  |  | CC2D2A |
|  |  | SLU7 |
|  |  | ZFP445 |
|  |  | GPATCH1 |
|  |  | DDX28 |
|  |  | MSL-1 |
|  |  | EZH1 |
|  |  | TMEM65 |
|  |  | CNR2 |
|  |  | LSP1 |
|  |  | CHP2 |
|  |  | FABP10A |
|  |  | CDK9 |
|  |  | CD59 |
|  |  | CAPZA1 |
|  |  | ARHGAP18 |
|  |  | DDIT4 |
|  |  | CRYGD |
|  |  | DNAJA2 |
|  |  | PTPRN2 |
|  |  | ITGA2B |
|  |  | PSAP |
|  |  | PTGDS |
|  |  | CHM |
|  |  | CCNL1 |
|  |  | SERINC5 |
|  |  | DUSP6 |
|  |  | TGFB3 |
|  |  | INPP4B |
|  |  | GMPPB |
|  |  | KLF7 |
|  |  | EAPP |
|  |  | LGI1B |
|  |  | IFI6 |
|  |  | COX6B1 |
|  |  | FRMD8 |
|  |  | USP31 |
|  |  | HIVEP2 |
|  |  | UBE2R2 |
|  |  | SUMO2 |
|  |  | SDCBP |
|  |  | PYGL |
|  |  | CTSG |
|  |  | TASOR2 |
|  |  | TXK |
|  |  | MTA3 |
|  |  | ICAM4 |
|  |  | CERS5 |
|  |  | SNRPA1 |
|  |  | ORC2 |
|  |  | LYG2 |
|  |  | HIST1H2BG |
|  |  | MCOLN1 |
|  |  | SLC16A6 |
|  |  | EIF5 |
|  |  | AIFM2 |
|  |  | PLA2G5 |
|  |  | PALB2 |
|  |  | MED17 |
|  |  | NLN |
|  |  | LPA |
|  |  | C8B |
|  |  | SASS6 |
|  |  | PABPC1L |
|  |  | STIL |
|  |  | NME7 |
|  |  | RPL5 |
|  |  | ZSCAN2 |
|  |  | HSPA9 |
|  |  | DENND1B |
|  |  | LSM5 |
|  |  | ZBTB1 |
|  |  | MIR23B |
|  |  | SLITRK5 |
|  |  | GINS4 |
|  |  | MYO15A |
|  |  | NDST2 |
|  |  | METTL22 |
|  |  | IL1R2 |
|  |  | NBPF10 |
|  |  | CYB5R1 |
|  |  | RAB3D |
|  |  | ZFC3H1 |
|  |  | SDC1 |
|  |  | ATP6V1H |
|  |  | FGA |
|  |  | HNRNPDL |
|  |  | SYK |
|  |  | IFI44L |
|  |  | IGFBP1 |
|  |  | ST20 |
|  |  | PSAT1 |
|  |  | ZNF766 |
|  |  | GDPD3 |
|  |  | TDG |
|  |  | TBC1D1 |
|  |  | MYCBP2 |
|  |  | ZNF226 |
|  |  | RETN |
|  |  | WIPI1 |
|  |  | COX18 |
|  |  | OGFOD3 |
|  |  | PUS7 |
|  |  | CCDC82 |
|  |  | PAFAH2 |
|  |  | TP53INP1 |
|  |  | ZBTB16B |
|  |  | CCDC80 |
|  |  | FDXACB1 |
|  |  | HTR6 |
|  |  | CAVIN4 |
|  |  | CAMK2A |
|  |  | FADS3 |
|  |  | RNF144B |
|  |  | SECISBP2L |
|  |  | PDZD8 |
|  |  | HTATIP2 |
|  |  | PAMR1 |
|  |  | CLU |
|  |  | ATG4C |
|  |  | PABPC4 |
|  |  | GUCY1B1 |
|  |  | TTTY8 |
|  |  | SPRR2A |
|  |  | IDI1 |
|  |  | KCTD6 |
|  |  | CORO1A |
|  |  | TRAF4 |
|  |  | CEP76 |
|  |  | COPS8 |
|  |  | SACM1L |
|  |  | AKAP9 |
|  |  | SLC20A1 |
|  |  | CX3CL1 |
|  |  | ARAP2 |
|  |  | NR5A2 |
|  |  | MYRFL |
|  |  | IGF1R |
|  |  | FAR1 |
|  |  | WDR72 |
|  |  | SF3B1 |
|  |  | A1BG |
|  |  | EFNB2 |
|  |  | IPO7 |
|  |  | DGAT2 |
|  |  | FMO5 |
|  |  | NOB1 |
|  |  | ABHD3 |
|  |  | HELLS |
|  |  | PIGN |
|  |  | RAMAC |
|  |  | THNSL1 |
|  |  | ANKRD2 |
|  |  | RETREG3 |
|  |  | SNRPN |
|  |  | HMGCL |
|  |  | ALX4 |
|  |  | MSI2 |
|  |  | MRTFA |
|  |  | COQ8A |
|  |  | SPARCL1 |
|  |  | TMCC1 |
|  |  | LRRC40 |
|  |  | IL6ST |
|  |  | PRSS23 |
|  |  | FAM172A |
|  |  | ASNS |
|  |  | L1CAMA |
|  |  | MMUT |
|  |  | IL22RA1 |
|  |  | CASZ1 |
|  |  | PPP4R3A |
|  |  | GIN1 |
|  |  | TMEM177 |
|  |  | CDKN2AIP |
|  |  | ANKRD29 |
|  |  | ABCB4 |
|  |  | SETDB2 |
|  |  | SGMS2 |
|  |  | HNF4A |
|  |  | TNFRSF10B |
|  |  | CDK5R1 |
|  |  | CTDSP1 |
|  |  | CHD4 |
|  |  | LGALS9C |
|  |  | FAM98C |
|  |  | SLC2A4 |
|  |  | RPS19 |
|  |  | DUT |
|  |  | LRRC59 |
|  |  | LARP1B |
|  |  | MAP2 |
|  |  | NOS3 |
|  |  | TRAC |
|  |  | CYP11B1 |
|  |  | MIR550 |
|  |  | FAM43A |
|  |  | TUSC1 |
|  |  | ZNF280B |
|  |  | RTP4 |
|  |  | FLRT2 |
|  |  | PPP4C |
|  |  | ANAPC5 |
|  |  | SLC47A2 |
|  |  | TNRC6C |
|  |  | KMT2A |
|  |  | RIT1 |
|  |  | FAM210A |
|  |  | XBP1 |
|  |  | FDX2 |
|  |  | CEBPA |
|  |  | MLF2 |
|  |  | C6ORF125 |
|  |  | S10AB |
|  |  | TMEM201 |
|  |  | ALOX12B |
|  |  | SIK2B |
|  |  | MLLT10 |
|  |  | ERCC6 |
|  |  | S100A10 |
|  |  | EIF2AK4 |
|  |  | PRDX2 |
|  |  | RPL13 |
|  |  | WDR76 |
|  |  | PRMT3 |
|  |  | PTPRO |
|  |  | CDKN1C |
|  |  | ZFAND3 |
|  |  | DOP1A |
|  |  | MBLAC2 |
|  |  | SPSB3 |
|  |  | GFAP |
|  |  | DPP8 |
|  |  | ACTB |
|  |  | TRMT12 |
|  |  | UBA2 |
|  |  | SLC39A3 |
|  |  | ANKRD12 |
|  |  | PCDH9 |
|  |  | RBM18 |
|  |  | HEXIM2 |
|  |  | ITFG1 |
|  |  | SLC30A5 |
|  |  | ACVR2B |
|  |  | MIRLET7D |
|  |  | LIN37 |
|  |  | SOCS2 |
|  |  | HIST4H4 |
|  |  | XKR6 |
|  |  | GBP3 |
|  |  | DOCK5 |
|  |  | DENND1C |
|  |  | CCDC15 |
|  |  | FAAP100 |
|  |  | PSMD1 |
|  |  | MIR107 |
|  |  | FBXO4 |
|  |  | TMEM128 |
|  |  | IFNGR1 |
|  |  | C2ORF42 |
|  |  | MBIP |
|  |  | SLC29A2 |
|  |  | RABL3 |
|  |  | PRELID1 |
|  |  | ARHGEF6 |
|  |  | INSR |
|  |  | ACMSD |
|  |  | IL33 |
|  |  | GLIPR1 |
|  |  | PHF13 |
|  |  | WWTR1 |
|  |  | CAMK2D1 |
|  |  | ZNF700 |
|  |  | CRACR2A |
|  |  | ZNF680 |
|  |  | NFX1 |
|  |  | MDN1 |
|  |  | CTNNAL1 |
|  |  | AMOTL1 |
|  |  | HERC4 |
|  |  | PEMT |
|  |  | PAICS |
|  |  | SYNJ1 |
|  |  | MMD |
|  |  | NDRG1A |
|  |  | SERPINB10 |
|  |  | ATP5F1B |
|  |  | KLF15 |
|  |  | SLCO1B2 |
|  |  | TCF7L2 |
|  |  | OGA |
|  |  | CDV3 |
|  |  | GPD1B |
|  |  | MYCBP |
|  |  | VANGL1 |
|  |  | QSER1 |
|  |  | NDUFA3 |
|  |  | HSP70 |
|  |  | NRCAM |
|  |  | FABP4 |
|  |  | SLC35E3 |
|  |  | LYRM4 |
|  |  | HIPK2 |
|  |  | ZMIZ1 |
|  |  | DAP |
|  |  | ENPP6 |
|  |  | MCM6 |
|  |  | C8ORF58 |
|  |  | EIF4A2 |
|  |  | MFSD13A |
|  |  | SMA4 |
|  |  | CES5A |
|  |  | RRM2B |
|  |  | PABPC1 |
|  |  | APOBEC3G |
|  |  | TMEFF1 |
|  |  | MIR130B |
|  |  | CLDN8 |
|  |  | STARD9 |
|  |  | NOCTA |
|  |  | TSR1 |
|  |  | HSD17B12 |
|  |  | E2F6 |
|  |  | AKR1B7 |
|  |  | CCDC85A |
|  |  | CTPS2 |
|  |  | SUDS3 |
|  |  | AHR2 |
|  |  | SCRIB |
|  |  | GPX3 |
|  |  | ZNF77 |
|  |  | TBCK |
|  |  | HPX |
|  |  | MALAT1 |
|  |  | EIF2S2 |
|  |  | UCHL5 |
|  |  | MLXIPL |
|  |  | C17ORF80 |
|  |  | ENC1 |
|  |  | MAP2K1 |
|  |  | GH1 |
|  |  | KYAT3 |
|  |  | AIM2 |
|  |  | TIMP2 |
|  |  | PTP4A1 |
|  |  | QRSL1 |
|  |  | JUB |
|  |  | SHC4 |
|  |  | FCRLA |
|  |  | MAK |
|  |  | MORC2 |
|  |  | STC2 |
|  |  | ZFP69 |
|  |  | COX4I1 |
|  |  | WNT4 |
|  |  | YIPF5 |
|  |  | IL11 |
|  |  | DIS3L |
|  |  | MIOS |
|  |  | RLIM |
|  |  | DTWD1 |
|  |  | TDO2 |
|  |  | STRN4 |
|  |  | HDGF |
|  |  | KRT6B |
|  |  | ARID4B |
|  |  | AKR1C3 |
|  |  | RNF19A |
|  |  | GMDS |
|  |  | CACNA2D4 |
|  |  | MIR22HG |
|  |  | CBX5 |
|  |  | KCTD12 |
|  |  | PPP2R5E |
|  |  | ASB7 |
|  |  | TNFSF9 |
|  |  | CFP |
|  |  | KCTD21 |
|  |  | MAP4K4 |
|  |  | MFNG |
|  |  | ZNF37A |
|  |  | CTSZ |
|  |  | DLAT |
|  |  | ZNF266 |
|  |  | KCNJ5 |
|  |  | LMNA |
|  |  | MIR3074 |
|  |  | MAP2K4A |
|  |  | KPNB1 |
|  |  | PRICKLE1 |
|  |  | PEG10 |
|  |  | WNT5B |
|  |  | CYP19A1B |
|  |  | ABCB1B |
|  |  | MIR431 |
|  |  | ZBTB5 |
|  |  | ZNF12 |
|  |  | GNGT2 |
|  |  | IFT20 |
|  |  | RAET1E |
|  |  | SGO2 |
|  |  | RFX7 |
|  |  | TSHB |
|  |  | SCN2B |
|  |  | RELN |
|  |  | CSRNP2 |
|  |  | C18ORF25 |
|  |  | MAGED2 |
|  |  | TRPS1 |
|  |  | CASP2 |
|  |  | SUPT7L |
|  |  | ADGRF4 |
|  |  | DOK7 |
|  |  | TESC |
|  |  | TFPI2 |
|  |  | YTHDC1 |
|  |  | ERN1 |
|  |  | JUN |
|  |  | NAGK |
|  |  | ADAMTS5 |
|  |  | PTH1R |
|  |  | B3GALT1 |
|  |  | RBP1 |
|  |  | ZKSCAN7 |
|  |  | CALHM2 |
|  |  | CRISPLD2 |
|  |  | RBM43 |
|  |  | AR |
|  |  | DDX3X |
|  |  | CYP3A |
|  |  | SESN2 |
|  |  | GATM |
|  |  | RASA3 |
|  |  | PLXDC1 |
|  |  | TENT5A |
|  |  | EPB41L5 |
|  |  | RND1 |
|  |  | MPG |
|  |  | IGKV4-1 |
|  |  | FJX1 |
|  |  | MDH |
|  |  | SCG5 |
|  |  | SH2D5 |
|  |  | E2F3 |
|  |  | MIR26A |
|  |  | NUDCD1 |
|  |  | TFF3 |
|  |  | NDC1 |
|  |  | WAC |
|  |  | ADCY9 |
|  |  | GOLGA4 |
|  |  | WHRN |
|  |  | VTG1 |
|  |  | SLC22A9 |
|  |  | SFRP1 |
|  |  | ST8SIA6 |
|  |  | DAPL1 |
|  |  | RMI2 |
|  |  | IDH3B |
|  |  | STXBP3 |
|  |  | GOLT1B |
|  |  | NEK3 |
|  |  | TNFRSF10D |
|  |  | FOSL1 |
|  |  | CRACR2B |
|  |  | UGT1A3 |
|  |  | CES1 |
|  |  | SMARCE1 |
|  |  | STK17B |
|  |  | ARHGAP27 |
|  |  | LRIG2 |
|  |  | SLC27A5 |
|  |  | GPAT3 |
|  |  | USP1 |
|  |  | EMSY |
|  |  | TTC7B |
|  |  | ZNF420 |
|  |  | C12ORF57 |
|  |  | TOB1 |
|  |  | SLC25A43 |
|  |  | C6 |
|  |  | SMAD5 |
|  |  | WASF2 |
|  |  | SLC16A10 |
|  |  | PRSS1 |
|  |  | KBTBD7 |
|  |  | PTGIS |
|  |  | KRT23 |
|  |  | UBC |
|  |  | ZNF200 |
|  |  | PUM1 |
|  |  | KLHL24 |
|  |  | CCDC88A |
|  |  | NADK2 |
|  |  | CNOT6L |
|  |  | S100A9 |
|  |  | HELQ |
|  |  | ARNT2 |
|  |  | ATP5MC3 |
|  |  | ZNHIT6 |
|  |  | WDR63 |
|  |  | FOLT-1 |
|  |  | 11-BETA-HSD3 |
|  |  | DCAF8 |
|  |  | FAM161B |
|  |  | HIST1H2BF |
|  |  | H2AFY |
|  |  | WDSUB1 |
|  |  | APBA1 |
|  |  | RAB2A |
|  |  | KCNJ2 |
|  |  | FAM106A |
|  |  | FIBIN |
|  |  | GAPDHS |
|  |  | GRAP2 |
|  |  | KRTAP2-2 |
|  |  | ZNF684 |
|  |  | TACC2 |
|  |  | PDHA1 |
|  |  | HSPB1 |
|  |  | NP1L1 |
|  |  | PMPCA |
|  |  | HSD17B1 |
|  |  | RNF38 |
|  |  | U2SURP |
|  |  | SLC7A6 |
|  |  | HERPUD2 |
|  |  | G3BP1 |
|  |  | HSD17B10 |
|  |  | NAALAD2 |
|  |  | UBE2G1 |
|  |  | RBM15 |
|  |  | GDF15 |
|  |  | CYCS |
|  |  | ATP9B |
|  |  | DAZAP1 |
|  |  | TLN1 |
|  |  | MITF |
|  |  | TPRG1L |
|  |  | CSTF1 |
|  |  | NSMCE4A |
|  |  | IGF2BP3 |
|  |  | FNBP1L |
|  |  | IMP4 |
|  |  | HRG |
|  |  | PON3 |
|  |  | EIF4A1B |
|  |  | ZNF131 |
|  |  | SP140L |
|  |  | G2E3 |
|  |  | NDUFS1 |
|  |  | TMCC3 |
|  |  | SLC38A5 |
|  |  | MIR15A |
|  |  | PAPOLA |
|  |  | NDUFAF4 |
|  |  | SLC16A14 |
|  |  | UGT2B17 |
|  |  | TET3 |
|  |  | CASP6 |
|  |  | BBC3 |
|  |  | PCSK1 |
|  |  | PI4KB |
|  |  | ANP32E |
|  |  | YEATS4 |
|  |  | ENDOU |
|  |  | FRY |
|  |  | FADS1 |
|  |  | CXADR |
|  |  | PCYT2 |
|  |  | TSC22D1 |
|  |  | ZNF182 |
|  |  | RHOT1 |
|  |  | EPS8 |
|  |  | ELOVL5 |
|  |  | ARHGAP21 |
|  |  | BLOC1S2 |
|  |  | HSD11B2 |
|  |  | RIOK1 |
|  |  | EGL-38 |
|  |  | HOOK3 |
|  |  | HNMT |
|  |  | PAPSS2 |
|  |  | CAND1 |
|  |  | ACSL3 |
|  |  | MIR103 |
|  |  | CDK11A |
|  |  | TBL3 |
|  |  | INTS6 |
|  |  | MIR374A |
|  |  | PDIA3 |
|  |  | ASB13 |
|  |  | GSS |
|  |  | DCP1A |
|  |  | KPNA3 |
|  |  | TAF5 |
|  |  | ANLN |
|  |  | HSPA1L |
|  |  | ZP3.2 |
|  |  | RHOC |
|  |  | SSTR2 |
|  |  | CLN5 |
|  |  | BIRC5 |
|  |  | PPP3CA |
|  |  | ZNF529 |
|  |  | CES2 |
|  |  | DDB2 |
|  |  | ATP5MC1 |
|  |  | FITM2 |
|  |  | MIR301 |
|  |  | SPTSSA |
|  |  | BCLAF1 |
|  |  | CYP2K21 |
|  |  | BMT2 |
|  |  | TRA2B |
|  |  | IL6RA |
|  |  | CDKL3 |
|  |  | PPIL4 |
|  |  | BBS7 |
|  |  | RIPK4 |
|  |  | GTF2H2 |
|  |  | MIB2 |
|  |  | EXOSC2 |
|  |  | MS4A2 |
|  |  | NPC2 |
|  |  | WDR59 |
|  |  | C12ORF65 |
|  |  | GUCY1A1 |
|  |  | HS3ST1 |
|  |  | GTF3C3 |
|  |  | MSMB |
|  |  | DNAJC5 |
|  |  | GATA6 |
|  |  | PF4 |
|  |  | MSH6 |
|  |  | IPO4 |
|  |  | SLC6A15 |
|  |  | MAPK12 |
|  |  | RIN2 |
|  |  | NR1D4A |
|  |  | POLR1E |
|  |  | PDE6C |
|  |  | EP400 |
|  |  | TRIM2 |
|  |  | KRTAP19-1 |
|  |  | MIR34B |
|  |  | LAMP3 |
|  |  | DNM1 |
|  |  | HYOU1 |
|  |  | UGT1A10 |
|  |  | ATOH8 |
|  |  | PLIN2 |
|  |  | USP7 |
|  |  | YWHAQ |
|  |  | ZSCAN16 |
|  |  | PPM1K |
|  |  | YME1L1 |
|  |  | HNRNPR |
|  |  | ENOSF1 |
|  |  | CARMIL1 |
|  |  | TPCN1 |
|  |  | STARD13 |
|  |  | ZNF75A |
|  |  | KLRG2 |
|  |  | GNAT1 |
|  |  | P4HA3 |
|  |  | ABHD2 |
|  |  | ORAI3 |
|  |  | DENND1A |
|  |  | QPRT |
|  |  | TRAM1 |
|  |  | TRIM29 |
|  |  | MPP4 |
|  |  | CDS1 |
|  |  | PPP1R9A |
|  |  | SLC22A6 |
|  |  | GPD1 |
|  |  | AVEN |
|  |  | NPM1 |
|  |  | PCK2 |
|  |  | DNMT3B |
|  |  | SDHAP2 |
|  |  | EDRF1 |
|  |  | TMEM200A |
|  |  | CCT8 |
|  |  | PDE4C |
|  |  | SYPL |
|  |  | EFHC1 |
|  |  | SER-7 |
|  |  | EBPL |
|  |  | GPR19 |
|  |  | HVCN1 |
|  |  | MRPS12 |
|  |  | GMFG |
|  |  | CRIPT |
|  |  | PENK |
|  |  | RGL1 |
|  |  | DLGAP4 |
|  |  | ATG10 |
|  |  | PHLDA1 |
|  |  | BACE2 |
|  |  | FAAP24 |
|  |  | INO80D |
|  |  | ZNF395 |
|  |  | PSMC3IP |
|  |  | H3F3B |
|  |  | TMEM87B |
|  |  | EZR |
|  |  | SLC38A4 |
|  |  | ATF1 |
|  |  | ZBTB18 |
|  |  | CLSTN2 |
|  |  | HINT3 |
|  |  | ARIH1 |
|  |  | PTGES |
|  |  | CALHM5 |
|  |  | BBS1 |
|  |  | RGPD5 |
|  |  | WDR20 |
|  |  | CST3 |
|  |  | PLD1 |
|  |  | CDC6 |
|  |  | NUP88 |
|  |  | CD300A |
|  |  | ATP5PD |
|  |  | HBB1 |
|  |  | NBR1 |
|  |  | LINC00572 |
|  |  | KIF2A |
|  |  | ZNF79 |
|  |  | ARL6IP6 |
|  |  | TENT5C |
|  |  | GLA |
|  |  | HSPA8 |
|  |  | DIDO1 |
|  |  | COG3 |
|  |  | CEL |
|  |  | IMMT |
|  |  | NGDN |
|  |  | CBFA2T2 |
|  |  | PICALM |
|  |  | SAC3D1 |
|  |  | HLF |
|  |  | ITPRIPL2 |
|  |  | BRD8 |
|  |  | TMED9 |
|  |  | UGT2B4 |
|  |  | HOXD13 |
|  |  | APOBEC3B |
|  |  | SLAIN1 |
|  |  | TCF4 |
|  |  | NR2C2 |
|  |  | RLN3A |
|  |  | GALNT18 |
|  |  | PHF1 |
|  |  | AURKA |
|  |  | AGTRAP |
|  |  | COLCA1 |
|  |  | RABEP2 |
|  |  | VWA1 |
|  |  | C6ORF52 |
|  |  | AAMDC |
|  |  | MIR182 |
|  |  | CED-9 |
|  |  | MTR |
|  |  | FN1 |
|  |  | FAM126B |
|  |  | ASH1L |
|  |  | VAMP4 |
|  |  | ZNF7 |
|  |  | TPT1P8 |
|  |  | GPR132 |
|  |  | SERTAD2 |
|  |  | ATP1A1 |
|  |  | SORCS2 |
|  |  | HEXIM1 |
|  |  | YWHAZ |
|  |  | OPA1 |
|  |  | CXORF21 |
|  |  | AURKB |
|  |  | IFI16 |
|  |  | CSTA |
|  |  | DZANK1 |
|  |  | LSM3 |
|  |  | LAMB3 |
|  |  | MOGS |
|  |  | RRAD |
|  |  | CFAP58 |
|  |  | MFSD4A |
|  |  | TMEM212 |
|  |  | RASSF4 |
|  |  | TP53I13 |
|  |  | SLC17A9 |
|  |  | GUSBP1 |
|  |  | ZFPM2 |
|  |  | C9ORF85 |
|  |  | SEC63 |
|  |  | GC |
|  |  | PIEZO2 |
|  |  | IER3 |
|  |  | MIR424 |
|  |  | ADRB1 |
|  |  | CEBPD |
|  |  | ZNF107 |
|  |  | ALDH6A1 |
|  |  | BCKDK |
|  |  | MOSPD1 |
|  |  | ZFP36 |
|  |  | PXN |
|  |  | SIRT2 |
|  |  | NTM |
|  |  | ASAP1 |
|  |  | CHAMP1 |
|  |  | EEPD1 |
|  |  | PGAM1 |
|  |  | GRM6 |
|  |  | MEIS1 |
|  |  | PCBP1 |
|  |  | LRRC2 |
|  |  | AMMECR1 |
|  |  | NSD1 |
|  |  | ANO9 |
|  |  | SEC13 |
|  |  | TTC30B |
|  |  | BRCC3 |
|  |  | CAV3 |
|  |  | YPEL5 |
|  |  | AQP8 |
|  |  | TMEM79 |
|  |  | DUSP14 |
|  |  | MIR101 |
|  |  | ABCC2 |
|  |  | TUBA1A |
|  |  | SGK1 |
|  |  | ZNF649 |
|  |  | COL5A2 |
|  |  | INS1 |
|  |  | CSPP1 |
|  |  | XRCC4 |
|  |  | MAP1LC3B |
|  |  | NAPB |
|  |  | ZNF827 |
|  |  | FNTB |
|  |  | ZNF518B |
|  |  | DPP7 |
|  |  | DSE |
|  |  | NFKBIZ |
|  |  | CAR2 |
|  |  | OSBPL3 |
|  |  | C3ORF38 |
|  |  | SERTAD1 |
|  |  | ANTXR1 |
|  |  | VTG |
|  |  | DGAT1 |
|  |  | ETV7 |
|  |  | CST8 |
|  |  | SENP8 |
|  |  | NKIRAS1 |
|  |  | TEX9 |
|  |  | C1ORF174 |
|  |  | RASSF10 |
|  |  | BARD1 |
|  |  | ZNF177 |
|  |  | MIP |
|  |  | SFTPD |
|  |  | FDX1 |
|  |  | EPS15 |
|  |  | GRIA1 |
|  |  | IL13RA1 |
|  |  | ZNF627 |
|  |  | TRPA1 |
|  |  | FDFT1 |
|  |  | CCDC90B |
|  |  | UBE2D3 |
|  |  | ZNF561 |
|  |  | GALNT2 |
|  |  | ENTPD2 |
|  |  | TRAPPC5 |
|  |  | ANKRD17 |
|  |  | JARID2A |
|  |  | STK3 |
|  |  | CHN2 |
|  |  | REPS2 |
|  |  | HAS2 |
|  |  | ANO10 |
|  |  | VEZT |
|  |  | CCNK |
|  |  | DNMT3A |
|  |  | CNOT7 |
|  |  | CST11 |
|  |  | CD200 |
|  |  | NKAP |
|  |  | EAF1 |
|  |  | SGK2 |
|  |  | TRIM35 |
|  |  | USP11 |
|  |  | HNF4G |
|  |  | IMPA2 |
|  |  | MSR1 |
|  |  | CCL4 |
|  |  | PDCD6IP |
|  |  | SPRR4 |
|  |  | AP2B1 |
|  |  | ZFP90 |
|  |  | RASSF9 |
|  |  | NET1 |
|  |  | IPO5 |
|  |  | MVD |
|  |  | KAT |
|  |  | CHDH |
|  |  | GCNT2 |
|  |  | ARHGAP12 |
|  |  | SURF4 |
|  |  | SLCO1B1 |
|  |  | ZNF565 |
|  |  | PLEKHO2 |
|  |  | NACA |
|  |  | DPY19L3 |
|  |  | NUDCD2 |
|  |  | ACE2 |
|  |  | SLCO1A4 |
|  |  | SPSB2 |
|  |  | TPPP3 |
|  |  | GIGYF2 |
|  |  | ITIH3 |
|  |  | GNAI2A |
|  |  | IRS2 |
|  |  | ACR-16 |
|  |  | RPL17 |
|  |  | HTR5A |
|  |  | USP |
|  |  | PDE4D |
|  |  | RITA1 |
|  |  | GEMIN4 |
|  |  | ZNF514 |
|  |  | EPSTI1 |
|  |  | ITGB8 |
|  |  | ABCD3A |
|  |  | ZNF354A |
|  |  | NTPCR |
|  |  | SELENOS |
|  |  | UBE2K |
|  |  | HIPK3 |
|  |  | BCAR3 |
|  |  | IL36G |
|  |  | DHCR7 |
|  |  | SCD1 |
|  |  | SLC19A1 |
|  |  | POR |
|  |  | SREBF2 |
|  |  | TRUB1 |
|  |  | TRIM14 |
|  |  | ENPP2 |
|  |  | SLC7A11 |
|  |  | ACAN |
|  |  | ZXDC |
|  |  | SLC31A2 |
|  |  | EOMES |
|  |  | CEP68 |
|  |  | PAX2 |
|  |  | LCE1C |
|  |  | NAA15 |
|  |  | FRMD4A |
|  |  | MCTP2 |
|  |  | LCMT2 |
|  |  | BIRC2 |
|  |  | PTGIR |
|  |  | MIR509 |
|  |  | ZC3H12C |
|  |  | HIST1H2AG |
|  |  | PIGK |
|  |  | WWOX |
|  |  | TRXR-2 |
|  |  | ABCA12 |
|  |  | KLF9 |
|  |  | FIGN |
|  |  | MIRLET7A-1 |
|  |  | PCSK2 |
|  |  | PI3 |
|  |  | RUNDC1 |
|  |  | MRC1 |
|  |  | MAVS |
|  |  | ZFAND6 |
|  |  | KNOP1 |
|  |  | PWWP2A |
|  |  | SDHAF1 |
|  |  | HIST1H2BE |
|  |  | ARID2 |
|  |  | RCC1 |
|  |  | BROX |
|  |  | PARS2 |
|  |  | GNA13 |
|  |  | POU2F2 |
|  |  | CHD1L |
|  |  | PDK4 |
|  |  | C12ORF76 |
|  |  | CNKSR2 |
|  |  | NUFIP1 |
|  |  | FILIP1 |
|  |  | CPZ |
|  |  | PTTG1 |
|  |  | IGFBP5 |
|  |  | TRNP |
|  |  | EPHB1 |
|  |  | SSBP2 |
|  |  | CASP8AP2 |
|  |  | CREB3L4 |
|  |  | TARBP1 |
|  |  | FBXO30 |
|  |  | COLEC12 |
|  |  | NME4 |
|  |  | CARD16 |
|  |  | MIR203 |
|  |  | IRF2BPL |
|  |  | MYOCD |
|  |  | OBI1 |
|  |  | PTGER4 |
|  |  | CHMP4C |
|  |  | CD68 |
|  |  | SMARCA1 |
|  |  | GCDH |
|  |  | APOF |
|  |  | ZBTB24 |
|  |  | NLRX1 |
|  |  | TEPSIN |
|  |  | DTX4 |
|  |  | BIN1 |
|  |  | TIMM8A |
|  |  | BNC1 |
|  |  | ZNF608 |
|  |  | NAP1L1 |
|  |  | C9ORF64 |
|  |  | GLCCI1 |
|  |  | CXL10 |
|  |  | MIPEP |
|  |  | ZFX |
|  |  | TRIP6 |
|  |  | IFT74 |
|  |  | PNISR |
|  |  | EI24 |
|  |  | NPAS2 |
|  |  | FAM111A |
|  |  | SDS |
|  |  | CASQ1 |
|  |  | CCDC174 |
|  |  | BLNK |
|  |  | DIAPH3 |
|  |  | CDC27 |
|  |  | ZNF767P |
|  |  | TAX1BP1B |
|  |  | DBP |
|  |  | VAC14 |
|  |  | COL4A2 |
|  |  | KDM5B |
|  |  | BPGM |
|  |  | TCF7L1 |
|  |  | GFPT1 |
|  |  | HMOX1 |
|  |  | ME2 |
|  |  | H3F3A |
|  |  | CD302 |
|  |  | F9A |
|  |  | SERPINB8 |
|  |  | ERAP2 |
|  |  | FEM1C |
|  |  | PKIG |
|  |  | PFAS |
|  |  | NUCKS1 |
|  |  | ND2 |
|  |  | IP6K2 |
|  |  | HNRNPD |
|  |  | GAREM1 |
|  |  | EXT1 |
|  |  | SMAD2 |
|  |  | TMEM68 |
|  |  | C15ORF61 |
|  |  | ZNF555 |
|  |  | PRKCZ |
|  |  | ZMYND19 |
|  |  | CENPE |
|  |  | DMAC2L |
|  |  | ASL |
|  |  | C8ORF48 |
|  |  | TFAM |
|  |  | PUM2 |
|  |  | RALGAPB |
|  |  | CMSS1 |
|  |  | SYNE3 |
|  |  | LDLRAP1 |
|  |  | PPP3CB |
|  |  | ANGPTL4 |
|  |  | PLCH1 |
|  |  | NAMPT |
|  |  | CYP2C75 |
|  |  | VKORC1L1 |
|  |  | PRAG1 |
|  |  | INSM1 |
|  |  | ZNF493 |
|  |  | TBCEL |
|  |  | RPS6 |
|  |  | HS3ST3B1 |
|  |  | SFXN1 |
|  |  | SPPL2A |
|  |  | FAM102A |
|  |  | OR51B4 |
|  |  | MIR434 |
|  |  | CES3 |
|  |  | PGF |
|  |  | FNIP2 |
|  |  | HSD17B3 |
|  |  | TKTB |
|  |  | PYGB |
|  |  | GADD45A |
|  |  | DKK3 |
|  |  | OARD1 |
|  |  | UTP18 |
|  |  | PPP1R14B |
|  |  | NHLRC2 |
|  |  | TRYP |
|  |  | ZNF430 |
|  |  | HIST1H2AM |
|  |  | TNRC6A |
|  |  | PLA2G1B |
|  |  | KAT14 |
|  |  | GTF2A2 |
|  |  | RCHY1 |
|  |  | RHOU |
|  |  | CYP27B1 |
|  |  | FTO |
|  |  | TACC3 |
|  |  | CCNJ |
|  |  | PSD |
|  |  | OXSM |
|  |  | SF3A1 |
|  |  | COPE |
|  |  | TNFSF11 |
|  |  | PTPRF |
|  |  | ATF4B |
|  |  | BLVRA |
|  |  | SLC22A7 |
|  |  | DANCR |
|  |  | SEC24B |
|  |  | LINS1 |
|  |  | IKBKB |
|  |  | SUSD3 |
|  |  | FXR2 |
|  |  | BTN2A2 |
|  |  | FRG1 |
|  |  | MYEOV |
|  |  | TYK2 |
|  |  | NIPSNAP2 |
|  |  | PIP4K2A |
|  |  | ZNF268 |
|  |  | MYLK |
|  |  | CYP4F2 |
|  |  | PGAP1 |
|  |  | KRTAP2-1 |
|  |  | ARL4D |
|  |  | MIRLET7B |
|  |  | MTMR4 |
|  |  | AQP3 |
|  |  | ST6GALNAC4 |
|  |  | GLMN |
|  |  | ZNF786 |
|  |  | C14ORF28 |
|  |  | IGFBP6 |
|  |  | HIST1H4D |
|  |  | ARHGEF28 |
|  |  | TSC22D2 |
|  |  | RNASE7 |
|  |  | RAB3GAP2 |
|  |  | RIOK2 |
|  |  | PALM3 |
|  |  | TASOR |
|  |  | CLEC7A |
|  |  | HSPA14 |
|  |  | CCPG1 |
|  |  | CFL1 |
|  |  | BAZ2B |
|  |  | EML6 |
|  |  | CPSF6 |
|  |  | GBP1 |
|  |  | PSMG1 |
|  |  | TAX1BP3 |
|  |  | KRT20 |
|  |  | RPL34 |
|  |  | BLCAP |
|  |  | SFI1 |
|  |  | HSD3B2 |
|  |  | GNE |
|  |  | WDR77 |
|  |  | CEP85L |
|  |  | ARL6IP5 |
|  |  | CYP3A65 |
|  |  | IARS |
|  |  | CD74 |
|  |  | LIPG |
|  |  | FEZ1 |
|  |  | EVI2B |
|  |  | SLC35E1 |
|  |  | APOD |
|  |  | DIRC3 |
|  |  | SYNCRIP |
|  |  | KCNJ12 |
|  |  | ELOVL2 |
|  |  | HDAC8 |
|  |  | ZNF589 |
|  |  | SCN1B |
|  |  | STAR |
|  |  | RNASEH2B |
|  |  | TRA2A |
|  |  | F2R |
|  |  | ESCO1 |
|  |  | MTAP |
|  |  | ADORA3 |
|  |  | TUBA1C |
|  |  | TRNT1 |
|  |  | ASMTL |
|  |  | KRTAP4-9 |
|  |  | AFDN |
|  |  | TMBIM6 |
|  |  | DNAJC27 |
|  |  | CAPS2 |
|  |  | DNAJC21 |
|  |  | STIP1 |
|  |  | NT5DC2 |
|  |  | MIR703 |
|  |  | CALN1 |
|  |  | CYBB |
|  |  | CYTIP |
|  |  | MTDH |
|  |  | HSP90AB1 |
|  |  | GEMIN2 |
|  |  | CHST7 |
|  |  | SEMA6D |
|  |  | PMAIP1 |
|  |  | HSPE1 |
|  |  | RSBN1 |
|  |  | S100A2 |
|  |  | ERO1LB |
|  |  | GSPT1 |
|  |  | FER |
|  |  | GTF2B |
|  |  | SNRK |
|  |  | IGHG1 |
|  |  | HYLS1 |
|  |  | MIR652 |
|  |  | GLDC |
|  |  | TMEM97 |
|  |  | CYP1C1 |
|  |  | PLA2G7 |
|  |  | FAM78AB |
|  |  | FUCA1 |
|  |  | MIR106A |
|  |  | KRT18 |
|  |  | MACC1 |
|  |  | CEP19 |
|  |  | KCMF1 |
|  |  | MCMDC2 |
|  |  | SOCS1 |
|  |  | STRA6 |
|  |  | PLPPR1 |
|  |  | SELENOI |
|  |  | FGF5 |
|  |  | IGHG3 |
|  |  | PHLDA2 |
|  |  | CNIH4 |
|  |  | FKBP15 |
|  |  | SLC35D1 |
|  |  | COA7 |
|  |  | RNF24 |
|  |  | CHCHD10 |
|  |  | MIR25 |
|  |  | CELF2 |
|  |  | HIST1H2BK |
|  |  | TENT5B |
|  |  | SAA1 |
|  |  | PF4V1 |
|  |  | REG1A |
|  |  | FBXW7 |
|  |  | RIOX1 |
|  |  | SBK1 |
|  |  | SETD1B |
|  |  | SSR1 |
|  |  | ADTRP |
|  |  | PDE4B |
|  |  | SLC25A36 |
|  |  | TMED10 |
|  |  | CSNK1G1 |
|  |  | KIF13A |
|  |  | MAFB |
|  |  | DLGAP5 |
|  |  | PPRC1 |
|  |  | PCDH17 |
|  |  | CYSRT1 |
|  |  | ADIPOR1 |
|  |  | RDH14 |
|  |  | DNAJB2 |
|  |  | TFPI |
|  |  | FABP6 |
|  |  | RC3H2 |
|  |  | TMEM87A |
|  |  | IFT27 |
|  |  | CYP2D2 |
|  |  | CYP17A1 |
|  |  | SEC62 |
|  |  | LINC00888 |
|  |  | DDX59 |
|  |  | ENO1 |
|  |  | PCBD1 |
|  |  | TAF3 |
|  |  | P4HA1 |
|  |  | AKAP8L |
|  |  | C9ORF152 |
|  |  | FLRT3 |
|  |  | NR2C1 |
|  |  | RNASE1 |
|  |  | SLC25A10 |
|  |  | ABHD17B |
|  |  | VPS54 |
|  |  | GLS |
|  |  | STS |
|  |  | FFAR2 |
|  |  | IL4R |
|  |  | GLI2 |
|  |  | ALDH1A3 |
|  |  | TGIF1 |
|  |  | CCDC69 |
|  |  | RAPGEF2 |
|  |  | NAV3 |
|  |  | EPS8L2 |
|  |  | ATP6V1G2 |
|  |  | ENTPD1 |
|  |  | GINM1 |
|  |  | NAT8F5 |
|  |  | PLPP3 |
|  |  | ZNF564 |
|  |  | PAXBP1 |
|  |  | EXOSC4 |
|  |  | MIR660 |
|  |  | BAG5 |
|  |  | HOXA-AS2 |
|  |  | BUD13 |
|  |  | RPS27L |
|  |  | CCN1 |
|  |  | CASP9 |
|  |  | PPA2 |
|  |  | MAP3K7 |
|  |  | EPOP |
|  |  | AKR1C1 |
|  |  | CALR3B |
|  |  | IMMP2L |
|  |  | BAG4 |
|  |  | GTF2I |
|  |  | ZNF469 |
|  |  | TOB1B |
|  |  | BRD2 |
|  |  | EGL-1 |
|  |  | RPS6KA1 |
|  |  | ICE2 |
|  |  | ZNF746 |
|  |  | SLC7A1 |
|  |  | C7ORF25 |
|  |  | MRPS15 |
|  |  | LAMC2 |
|  |  | SPSB1 |
|  |  | TIRAP |
|  |  | ADAM17 |
|  |  | CTNND1 |
|  |  | LINC00968 |
|  |  | CPNE1 |
|  |  | ZC3H4 |
|  |  | RPL37A |
|  |  | CPS1 |
|  |  | ELAVL1 |
|  |  | N4BP1 |
|  |  | BNIP3L |
|  |  | CFLAR |
|  |  | WDR48 |
|  |  | TOR4A |
|  |  | GUK1 |
|  |  | TRAK1 |
|  |  | STXBP1 |
|  |  | USP12 |
|  |  | CUTC |
|  |  | AGPAT5 |
|  |  | FGD3 |
|  |  | PREPL |
|  |  | HINFP |
|  |  | MIR150 |
|  |  | SERF2 |
|  |  | RABGAP1L |
|  |  | TRNH |
|  |  | PTGR2 |
|  |  | CD22 |
|  |  | CD47 |
|  |  | DLC1 |
|  |  | PRKACA |
|  |  | C1ORF131 |
|  |  | SPRY4 |
|  |  | ELMSAN1 |
|  |  | MXD1 |
|  |  | PNPLA7 |
|  |  | ASXL1 |
|  |  | TUBB4B |
|  |  | RBP2A |
|  |  | WDR37 |
|  |  | AZI2 |
|  |  | NUAK2 |
|  |  | CERT1 |
|  |  | AHSG |
|  |  | RBM39 |
|  |  | IMP3 |
|  |  | INHA |
|  |  | FAM104B |
|  |  | LMOD2 |
|  |  | C8ORF82 |
|  |  | NFXL1 |
|  |  | NUDT4 |
|  |  | WHAMM |
|  |  | USO1 |
|  |  | RUFY3 |
|  |  | MIR484 |
|  |  | CLDN1 |
|  |  | FASTK |
|  |  | UBALD2 |
|  |  | LDB1 |
|  |  | CD164 |
|  |  | GSTA5 |
|  |  | SHLD2 |
|  |  | MIR27B |
|  |  | RBP3 |
|  |  | REN |
|  |  | ACTA1 |
|  |  | NSG1 |
|  |  | PLEKHG1 |
|  |  | GABBR2 |
|  |  | SP8 |
|  |  | UBFD1 |
|  |  | HIST1H2BD |
|  |  | VGF |
|  |  | TIMM17B |
|  |  | IGF2R |
|  |  | SBSN |
|  |  | IL12B |
|  |  | FCER1G |
|  |  | SIAE |
|  |  | TMEM107 |
|  |  | DEDD2 |
|  |  | TXNL4B |
|  |  | MT1A |
|  |  | RPL10L |
|  |  | ZBTB3 |
|  |  | NCAN |
|  |  | TOP2B |
|  |  | NPVF |
|  |  | ORC4 |
|  |  | RAD54L2 |
|  |  | ZNF207 |
|  |  | CLEC11A |
|  |  | ZFAND2A |
|  |  | PFKFB4 |
|  |  | CEACAM1 |
|  |  | SREBF1 |
|  |  | LRRC57 |
|  |  | SCMH1 |
|  |  | COL8A2 |
|  |  | TXLNB |
|  |  | PHB |
|  |  | CYP8B1 |
|  |  | ZNF91 |
|  |  | HSDL2 |
|  |  | POU2F1 |
|  |  | GAPDH |
|  |  | TMEM184C |
|  |  | ERRFI1 |
|  |  | COX7C |
|  |  | BSPRY |
|  |  | PGAM2 |
|  |  | PEX12 |
|  |  | CNNM2 |
|  |  | CCDC152 |
|  |  | ELK3 |
|  |  | GPX1 |
|  |  | RSL1D1 |
|  |  | ADNP2 |
|  |  | PLEKHA5 |
|  |  | MECR |
|  |  | PSCA |
|  |  | BCL10 |
|  |  | GAS6 |
|  |  | TMTC1 |
|  |  | USP28 |
|  |  | ERGIC2 |
|  |  | ASB9 |
|  |  | SULT2B1 |
|  |  | KDM6BB |
|  |  | TRAFD1 |
|  |  | CTTNBP2NL |
|  |  | RGS7BP |
|  |  | ME1 |
|  |  | CDA |
|  |  | TCN2 |
|  |  | ZFY |
|  |  | FSD1L |
|  |  | PPARGC1B |
|  |  | EPHB2 |
|  |  | MRPL30 |
|  |  | ACTG1 |
|  |  | IVL |
|  |  | CALM1 |
|  |  | CTRB1 |
|  |  | ZP2 |
|  |  | CYFIP2 |
|  |  | CKM |
|  |  | SCAF4 |
|  |  | HACD2 |
|  |  | MAO |
|  |  | DEPP1 |
|  |  | CUEDC1B |
|  |  | APRT |
|  |  | PCSK7 |
|  |  | SIK1 |
|  |  | TP53RK |
|  |  | KAT7 |
|  |  | HSD17B6 |
|  |  | RUFY2 |
|  |  | SLC16A1 |
|  |  | PIM2 |
|  |  | HGF |
|  |  | INSIG1 |
|  |  | IFRG15 |
|  |  | CFD |
|  |  | MIR29A |
|  |  | CDK3 |
|  |  | ZNF235 |
|  |  | ACSL4 |
|  |  | C2ORF49 |
|  |  | ARL8B |
|  |  | PPM1D |
|  |  | TRNA |
|  |  | KAT2B |
|  |  | CYP1B1 |
|  |  | AOC2 |
|  |  | KCP |
|  |  | C6ORF62 |
|  |  | BRWD1 |
|  |  | CYP19A1A |
|  |  | PHF23 |
|  |  | C9 |
|  |  | CD3EAP |
|  |  | ZBED5 |
|  |  | LTA4H |
|  |  | DUSP13 |
|  |  | IFIT3 |
|  |  | CKAP4 |
|  |  | ZNF224 |
|  |  | TMTC2 |
|  |  | MCTP1 |
|  |  | PDE3B |
|  |  | TRIM68 |
|  |  | ZNF652 |
|  |  | L3MBTL1 |
|  |  | PGK1 |
|  |  | ACER1 |
|  |  | FAM50A |
|  |  | OAT |
|  |  | IQGAP2 |
|  |  | CDYL |
|  |  | RLF |
|  |  | MTFR1 |
|  |  | C6ORF58 |
|  |  | RBP5 |
|  |  | LTV1 |
|  |  | E2F8 |
|  |  | OTUD1 |
|  |  | DHCR24 |
|  |  | MYO1B |
|  |  | VAMP8 |
|  |  | HPN |
|  |  | ANKRA2 |
|  |  | PANX1 |
|  |  | WASL |
|  |  | CYBA |
|  |  | TAF5L |
|  |  | RGMB |
|  |  | SOS1 |
|  |  | ARHGAP11A |
|  |  | KMT2E |
|  |  | MOB2 |
|  |  | FRMD3 |
|  |  | SETD2 |
|  |  | PACSIN2 |
|  |  | SERPINB5 |
|  |  | TIA1 |
|  |  | ZBTB34 |
|  |  | TMOD4 |
|  |  | SYNE2 |
|  |  | GPR180 |
|  |  | SLC22A4 |
|  |  | ZNF826P |
|  |  | VCPIP1 |
|  |  | PHLDA3 |
|  |  | KLHDC4 |
|  |  | DOCK2 |
|  |  | GABARAP |
|  |  | SNHG4 |
|  |  | FABP7 |
|  |  | GOLM1 |
|  |  | IFT46 |
|  |  | KEL |
|  |  | SSH2 |
|  |  | KCTD15 |
|  |  | EIF3B |
|  |  | SLCO1B3 |
|  |  | BIK |
|  |  | ACAD8 |
|  |  | TRIM22 |
|  |  | RNASE6 |
|  |  | GPR3 |
|  |  | RAB8B |
|  |  | GJA1 |
|  |  | FASN |
|  |  | METTL8 |
|  |  | PHLPP2 |
|  |  | SLC39A7 |
|  |  | CAMK1 |
|  |  | FBXO8 |
|  |  | RFC3 |
|  |  | NAPSB |
|  |  | ZNF506 |
|  |  | FARSB |
|  |  | NOM1 |
|  |  | GPX7 |
|  |  | SMIM3 |
|  |  | ASCC3 |
|  |  | BASP1 |
|  |  | ZNF202 |
|  |  | CLDN4 |
|  |  | SLC22A18 |
|  |  | DNAJC22 |
|  |  | TBK1 |
|  |  | NR1H3 |
|  |  | PHACTR3 |
|  |  | TRNR |
|  |  | CRLS1 |
|  |  | MIR18A |
|  |  | WFDC5 |
|  |  | SQSTM1 |
|  |  | LRRC8C |
|  |  | MAN2A2 |
|  |  | PLK3 |
|  |  | ADAMDEC1 |
|  |  | APOL6 |
|  |  | MRPS34 |
|  |  | PLK1 |
|  |  | TMEM267 |
|  |  | DAB1 |
|  |  | ZC3H7A |
|  |  | LAIR2 |
|  |  | RTN4R |
|  |  | RFTN1 |
|  |  | ATP8B2 |
|  |  | BTG1 |
|  |  | KLHL23 |
|  |  | APOC3 |
|  |  | ZNF148 |
|  |  | TEKT4P2 |
|  |  | AMN1 |
|  |  | CNOT4 |
|  |  | HIF3A |
|  |  | CAPZA2 |
|  |  | PLA2G2A |
|  |  | LPGAT1 |
|  |  | TEX19 |
|  |  | MAPKAP1 |
|  |  | SRPRA |
|  |  | UBQLN4 |
|  |  | VAMP1 |
|  |  | HIST1H2AE |
|  |  | SOCS6 |
|  |  | WDR1 |
|  |  | FAM27E3 |
|  |  | PIWI |
|  |  | PM20D2 |
|  |  | ADAT2 |
|  |  | PPARB |
|  |  | MGST3 |
|  |  | ZNF596 |
|  |  | CDKN1B |
|  |  | LDLRAD4 |
|  |  | USP49 |
|  |  | NDUFS8 |
|  |  | SLC25A16 |
|  |  | STEAP3 |
|  |  | EIF2B3 |
|  |  | MIR454 |
|  |  | ABCA1 |
|  |  | FGG |
|  |  | CLEC2B |
|  |  | MIR433 |
|  |  | CEP170B |
|  |  | DTX3L |
|  |  | FNIP1 |
|  |  | EVI1 |
|  |  | TMEM176L.1 |
|  |  | SLC2A3 |
|  |  | MIR9 |
|  |  | FEM1B |
|  |  | ACOX1 |
|  |  | STOML1 |
|  |  | MTHFSD |
|  |  | EFNA4 |
|  |  | MAEL |
|  |  | NUP153 |
|  |  | MACO1 |
|  |  | LBP |
|  |  | BDKRB2 |
|  |  | HSD17B4 |
|  |  | IDH2 |
|  |  | ELF3 |
|  |  | AMZ2P1 |
|  |  | SLMAPA |
|  |  | DCLK1 |
|  |  | MYEF2 |
|  |  | AGPAT3 |
|  |  | ADORA2A |
|  |  | HISTH4 |
|  |  | KCNH2 |
|  |  | CAMK2D |
|  |  | SYPL2 |
|  |  | ADCY5 |
|  |  | TNNI3 |
|  |  | HIST1H2BC |
|  |  | E2F1 |
|  |  | CTSV |
|  |  | NPAT |
|  |  | CLP1 |
|  |  | KLHDC7B |
|  |  | GPATCH2L |
|  |  | COIL |
|  |  | TSC22D3 |
|  |  | PRKD3 |
|  |  | CASP8 |
|  |  | RAB11FIP2 |
|  |  | NRIP1 |
|  |  | ACP2 |
|  |  | LAG3 |
|  |  | METRN |
|  |  | ARID5B |
|  |  | MOG |
|  |  | KRAS |
|  |  | SIPA1L2 |
|  |  | CADM3-AS1 |
|  |  | SPTAN1 |
|  |  | CCNG2 |
|  |  | RCAN2 |
|  |  | DHB14 |
|  |  | NECAP2 |
|  |  | TENM2 |
|  |  | PPARG |
|  |  | BBS10 |
|  |  | PPP2R1B |
|  |  | TANC1 |
|  |  | PRDM4 |
|  |  | PAPPA |
|  |  | MMAB |
|  |  | SOD-1 |
|  |  | SERPINB7 |
|  |  | RAB26 |
|  |  | DCTN3 |
|  |  | CXCL11 |
|  |  | LSM10 |
|  |  | ZNF639 |
|  |  | TNNC1 |
|  |  | AKR1C2 |
|  |  | CACNG4 |
|  |  | HCAR3 |
|  |  | SUN1 |
|  |  | MYL6 |
|  |  | NAGA |
|  |  | NIBAN1 |
|  |  | SMC4 |
|  |  | SMIM10L1 |
|  |  | POMZP3 |
|  |  | ZNF441 |
|  |  | MTTP |
|  |  | RPL22L1 |
|  |  | BAXA |
|  |  | ZNF138 |
|  |  | GZMK |
|  |  | MIR374B |
|  |  | LITAF |
|  |  | DYM |
|  |  | ABHD15 |
|  |  | AFP |
|  |  | RAB21 |
|  |  | CRY1 |
|  |  | ZNF343 |
|  |  | ZCCHC24 |
|  |  | IFIT2 |
|  |  | PIK3R3 |
|  |  | NR3C1 |
|  |  | USPL1 |
|  |  | NKG7 |
|  |  | PPP2R3D |
|  |  | ATP2A1 |
|  |  | CHCHD7 |
|  |  | PAXIP1 |
|  |  | ZC3HAV1 |
|  |  | VTG2 |
|  |  | ZNF92 |
|  |  | CCDC138 |
|  |  | FAM222B |
|  |  | CKS1B |
|  |  | CLPXB |
|  |  | USP13 |
|  |  | RPS2 |
|  |  | NBPF1 |
|  |  | SNRPE |
|  |  | LEPROT |
|  |  | STK38L |
|  |  | STEAP4 |
|  |  | TXNRD2 |
|  |  | KSR1 |
|  |  | CBLB |
|  |  | NME6 |
|  |  | MIIP |
|  |  | MIR1187 |
|  |  | C6ORF223 |
|  |  | SNAPC4 |
|  |  | CDK6 |
|  |  | SLC22A11 |
|  |  | LRIG3 |
|  |  | P2RY1 |
|  |  | CSK |
|  |  | RAB27B |
|  |  | ZNF236 |
|  |  | MORF4L2 |
|  |  | RPL7 |
|  |  | LILRA2 |
|  |  | FBLN1 |
|  |  | IFT22 |
|  |  | MYOF |
|  |  | MRRF |
|  |  | TMTC3 |
|  |  | PRKAB2 |
|  |  | LUC7L |
|  |  | AFF4 |
|  |  | CES2C |
|  |  | DNSL3 |
|  |  | INF2 |
|  |  | SUB1 |
|  |  | PRL2C2 |
|  |  | CRB2 |
|  |  | SLC16A9 |
|  |  | RAMP1 |
|  |  | KIF20B |
|  |  | P2RX5 |
|  |  | ANKRD46 |
|  |  | GAS2L3 |
|  |  | RSRP1 |
|  |  | SNX4 |
|  |  | CCR2 |
|  |  | LMBRD2 |
|  |  | CHST4 |
|  |  | SYNRG |
|  |  | SMAD7 |
|  |  | SIRPB1 |
|  |  | RHBDD2 |
|  |  | GATAD1 |
|  |  | SDC4 |
|  |  | FHL2 |
|  |  | FCER1A |
|  |  | WARS |
|  |  | SNRPD1 |
|  |  | ITGAV |
|  |  | BZW1 |
|  |  | ASPA |
|  |  | GDAP1 |
|  |  | RBBP4 |
|  |  | AMY2A |
|  |  | PPP2CB |
|  |  | HSD3B1 |
|  |  | ZRANB1 |
|  |  | AP1M1 |
|  |  | PRELID3B |
|  |  | CYC1 |
|  |  | RGS7 |
|  |  | ASIC1 |
|  |  | DISP1 |
|  |  | HR |
|  |  | GM2A |
|  |  | MIR29B |
|  |  | RAB33A |
|  |  | ETV4 |
|  |  | CPT1B |
|  |  | CENPS |
|  |  | TRNY |
|  |  | CYP2K6 |
|  |  | UNC-115 |
|  |  | ZFP30 |
|  |  | ABCG2A |
|  |  | MYH10 |
|  |  | TTC39C |
|  |  | CTBP1 |
|  |  | CAT-4 |
|  |  | TBXAS1 |
|  |  | STXBP5 |
|  |  | ALDH1B1 |
|  |  | COX1 |
|  |  | USP25 |
|  |  | PRKX |
|  |  | ZNF124 |
|  |  | ARG1 |
|  |  | GFM1 |
|  |  | FCRL2 |
|  |  | HBD |
|  |  | PCK1 |
|  |  | RAD51C |
|  |  | MYO5A |
|  |  | IRAK3 |
|  |  | ZC3HAV1L |
|  |  | REV1 |
|  |  | SPEN |
|  |  | CCSAP |
|  |  | GNA15 |
|  |  | GNG4 |
|  |  | DRAM1 |
|  |  | TSPAN33 |
|  |  | P2RY12 |
|  |  | SNORA73A |
|  |  | TTC32 |
|  |  | ZNF217 |
|  |  | CDH26 |
|  |  | NOX4 |
|  |  | VWF |
|  |  | ZBTB26 |
|  |  | BAD |
|  |  | TPM1 |
|  |  | PYHIN1 |
|  |  | SALL2 |
|  |  | ZNF644 |
|  |  | ZNF101 |
|  |  | WDFY4 |
|  |  | CTH |
|  |  | ITPRIPL1 |
|  |  | SLC4A2 |
|  |  | ATP5F1A |
|  |  | CRYGN1 |
|  |  | ANGPTL8 |
|  |  | SLCO1A2 |
|  |  | HNRNPUL1 |
|  |  | PFKFB3 |
|  |  | SIN3A |
|  |  | SFRP4 |
|  |  | ELA2L |
|  |  | OSTM1 |
|  |  | AGTR1 |
|  |  | SELENOM |
|  |  | OTP |
|  |  | NAPEPLD |
|  |  | PDCD2L |
|  |  | CYP2F2 |
|  |  | AHSA2P |
|  |  | USP2 |
|  |  | ISG20L2 |
|  |  | MAN2A1 |
|  |  | TMM27 |
|  |  | CLCA2 |
|  |  | RND3 |
|  |  | ESD |
|  |  | PNRC1 |
|  |  | IL1RAP |
|  |  | UBXN2B |
|  |  | CSNK1D |
|  |  | VDAC1 |
|  |  | TRIB1 |
|  |  | RALGDS |
|  |  | PER2 |
|  |  | PRMT6 |
|  |  | ZKSCAN4 |
|  |  | RBM12B |
|  |  | BNIP4 |
|  |  | TAF4 |
|  |  | GABPB1 |
|  |  | ERICH3 |
|  |  | DQX1 |
|  |  | ERI2 |
|  |  | RETNLB |
|  |  | HPGD |
|  |  | ARHGAP19 |
|  |  | S100A6 |
|  |  | AMACR |
|  |  | SMIM19 |
|  |  | NEDD1 |
|  |  | TINF2 |
|  |  | EPC1 |
|  |  | OCIAD1 |
|  |  | RBPMS2 |
|  |  | DDX46 |
|  |  | AK2 |
|  |  | THRSP |
|  |  | VPS13A |
|  |  | BCOR |
|  |  | SH3TC1 |
|  |  | CMTM6 |
|  |  | BHLHB9 |
|  |  | PNPLA8 |
|  |  | REG3G |
|  |  | LARP6 |
|  |  | LIN-39 |
|  |  | SLC35F3 |
|  |  | NANP |
|  |  | ZNF621 |
|  |  | FANCF |
|  |  | INHBB |
|  |  | TBL1Y |
|  |  | LONP2 |
|  |  | TADA1 |
|  |  | ADRM1 |
|  |  | ATP5ME |
|  |  | CED-4 |
|  |  | MAP3K1 |
|  |  | ZFP82 |
|  |  | ZBED8 |
|  |  | CPPED1 |
|  |  | ALDOA |
|  |  | AIMP2 |
|  |  | SPATS2 |
|  |  | COA1 |
|  |  | ABHD17C |
|  |  | ATF4 |
|  |  | ZNF292 |
|  |  | IL12A |
|  |  | ARNTL |
|  |  | RAPGEF4 |
|  |  | CTCF |
|  |  | AADAT |
|  |  | SNTB2 |
|  |  | SURF2 |
|  |  | KPNA1 |
|  |  | KRT81 |
|  |  | TRNE |
|  |  | CCND3 |
|  |  | CEP95 |
|  |  | S100A11 |
|  |  | SPATA7 |
|  |  | CLASP2 |
|  |  | EIF2AK3 |
|  |  | PDCD10 |
|  |  | PDE1A |
|  |  | RSBN1L |
|  |  | ZBTB8A |
|  |  | NXT1 |
|  |  | ENKD1 |
|  |  | PSME4 |
|  |  | TFG |
|  |  | ECI2 |
|  |  | EPHX2 |
|  |  | MGARP |
|  |  | XLR3C |
|  |  | CCL24 |
|  |  | ZMAT3 |
|  |  | ANGPT1 |
|  |  | MED23 |
|  |  | PCNX1 |
|  |  | PTGDR2 |
|  |  | CCDC8 |
|  |  | VIPR2 |
|  |  | CHAC1 |
|  |  | LIMK2 |
|  |  | MTPAP |
|  |  | HHAT |
|  |  | ING2 |
|  |  | UBE4A |
|  |  | TRPM4 |
|  |  | LTB4R |
|  |  | MX2 |
|  |  | C11ORF49 |
|  |  | IDH3A |
|  |  | RAB39B |
|  |  | CREB5 |
|  |  | ICK |
|  |  | ANGPT2 |
|  |  | NDUFC2 |
|  |  | CCDC68 |
|  |  | EEF2L2 |
|  |  | TRAPPC13 |
|  |  | PARP1 |
|  |  | SIAH1 |
|  |  | SLC25A32A |
|  |  | DAPK2B |
|  |  | GZF1 |
|  |  | TRNK |
|  |  | RPS15 |
|  |  | GOLGA7 |
|  |  | FERMT2 |
|  |  | DNAJC6 |
|  |  | CLOCK |
|  |  | MLX |
|  |  | SLC16A6B |
|  |  | GCNA |
|  |  | TPD52 |
|  |  | RPS16P5 |
|  |  | ROCK1 |
|  |  | SLCO4A1 |
|  |  | NCAPH |
|  |  | M1AP |
|  |  | AFG3L1 |
|  |  | PTPRK |
|  |  | TAGLN |
|  |  | STK11IP |
|  |  | SLC25A25 |
|  |  | ZC3H13 |
|  |  | ZNF45 |
|  |  | PHACTR4 |
|  |  | TK1 |
|  |  | ZNF329 |
|  |  | SNHG3 |
|  |  | PGP |
|  |  | VEZF1 |
|  |  | TBC1D4 |
|  |  | CAMKMT |
|  |  | MSL2 |
|  |  | PLA2G3 |
|  |  | MOXD1 |
|  |  | CCDC84 |
|  |  | FAM13C |
|  |  | SREK1IP1 |
|  |  | MYLIP |
|  |  | PHIP |
|  |  | SCAPER |
|  |  | DENND5A |
|  |  | NUP98 |
|  |  | HSPA13 |
|  |  | TMEM158 |
|  |  | KDELR2B |
|  |  | FASTKD3 |
|  |  | BDH1 |
|  |  | MED4 |
|  |  | IKBIP |
|  |  | SS18 |
|  |  | ECM2 |
|  |  | TAF6 |
|  |  | FN1B |
|  |  | IKZF2 |
|  |  | DOCK9 |
|  |  | TAF1A |
|  |  | FKSG49 |
|  |  | HIST1H3H |
|  |  | ZC3H7B |
|  |  | PDE2A |
|  |  | SOCS7 |
|  |  | TUBB2A |
|  |  | HSP47 |
|  |  | ASPM |
|  |  | ACP5B |
|  |  | LRP6 |
|  |  | RNA45SN4 |
|  |  | RRP8 |
|  |  | TTLL12 |
|  |  | MIR650 |
|  |  | PPARA |
|  |  | MGLL |
|  |  | MN1 |
|  |  | SCFD2 |
|  |  | RORA |
|  |  | HSD3B4 |
|  |  | DENND2C |
|  |  | PLK2 |
|  |  | LONRF3 |
|  |  | SMURF1 |
|  |  | ANKRD39 |
|  |  | TNFSF4 |
|  |  | TVP23B |
|  |  | CSTF2 |
|  |  | OPTN |
|  |  | BCL2L11 |
|  |  | SCPEP1 |
|  |  | INHBA |
|  |  | RBP4 |
|  |  | METTL9 |
|  |  | S100A4 |
|  |  | GPR137C |
|  |  | MAP2K5 |
|  |  | PLCB1 |
|  |  | SRXN1 |
|  |  | CPEB2 |
|  |  | IGF2 |
|  |  | NPEPPS |
|  |  | LRRK1 |
|  |  | DENR |
|  |  | SENP2 |
|  |  | JKAMP |
|  |  | MEIG1 |
|  |  | STARD10 |
|  |  | DNMT1 |
|  |  | PCGF5 |
|  |  | JMJD6 |
|  |  | RNF26 |
|  |  | ADAMTS1 |
|  |  | ZNF571 |
|  |  | PRKG1 |
|  |  | ZNF283 |
|  |  | TRIM21 |
|  |  | CADM3 |
|  |  | SBF2 |
|  |  | PCF11 |
|  |  | FABP1 |
|  |  | CYP20A1 |
|  |  | MBD6 |
|  |  | ACKR1 |
|  |  | SORBS1 |
|  |  | ERV3-2 |
|  |  | ATRAID |
|  |  | PEA15 |
|  |  | SLC15A1 |
|  |  | TAT |
|  |  | LRCH2 |
|  |  | KLK13 |
|  |  | GSPT2 |
|  |  | TRIM13 |
|  |  | FNBP4 |
|  |  | STXBP6 |
|  |  | CHST9 |
|  |  | MXI1 |
|  |  | CLCN5 |
|  |  | DRAM2B |
|  |  | FBXW5 |
|  |  | IL3RA |
|  |  | TSEN2 |
|  |  | SAA3 |
|  |  | MAG |
|  |  | MAP3K2 |
|  |  | JMY |
|  |  | KMT5B |
|  |  | CLINT1 |
|  |  | ADORA1 |
|  |  | ZNF451 |
|  |  | SYMPK |
|  |  | UCP1 |
|  |  | RTTN |
|  |  | POLE2 |
|  |  | UGT2B1 |
|  |  | ARRDC3 |
|  |  | STX12 |
|  |  | VNN1 |
|  |  | CED-3 |
|  |  | ZNF322 |
|  |  | DDIT3 |
|  |  | CYLC1 |
|  |  | HOMER1 |
|  |  | ZNF286A |
|  |  | OSR1 |
|  |  | ZNF302 |
|  |  | ENY2 |
|  |  | CALU |
|  |  | PTGR1 |
|  |  | SAP18 |
|  |  | HSPA5 |
|  |  | LIMCH1 |
|  |  | JUNB |
|  |  | 4-Mar |
|  |  | RWDD1 |
|  |  | ALDH8A1 |
|  |  | ASIC2 |
|  |  | TSHZ3 |
|  |  | RHOB |
|  |  | RALA |
|  |  | IGDCC4 |
|  |  | MMAA |
|  |  | CCNG1 |
|  |  | MGME1 |
|  |  | ZNF526 |
|  |  | ACAT2 |
|  |  | GNMT |
|  |  | PPP2R1A |
|  |  | RPAIN |
|  |  | TET2 |
|  |  | EPG5 |
|  |  | TMEM173 |
|  |  | SRSF1 |
|  |  | IFI30 |
|  |  | EEF1E1 |
|  |  | GLUL |
|  |  | AUNIP |
|  |  | ESF1 |
|  |  | ANAPC16 |
|  |  | HK2 |
|  |  | NOTCH2NLA |
|  |  | ELOVL6 |
|  |  | METRNL |
|  |  | IAH1 |
|  |  | ZNF14 |
|  |  | SLC27A4 |
|  |  | AVPR1A |
|  |  | TPK1 |
|  |  | MICAL2 |
|  |  | CYP2C23 |
|  |  | COL14A1 |
|  |  | CPNE3 |
|  |  | ERO1A |
|  |  | DNPH1 |
|  |  | ATP8A1 |
|  |  | IFNA |
|  |  | PARP10 |
|  |  | PLPP6 |
|  |  | ANKRD50 |
|  |  | C12ORF66 |
|  |  | MKNK2B |
|  |  | BCL2L1 |
|  |  | MIRLET7I |
|  |  | EIF2A |
|  |  | GBP6 |
|  |  | JARID2 |
|  |  | USP34 |
|  |  | LEV-8 |
|  |  | CRBN |
|  |  | PRR18 |
|  |  | PTGS2A |
|  |  | CHIC2 |
|  |  | ATPAF1 |
|  |  | ITIH2 |
|  |  | ZNF367 |
|  |  | NCBP1 |
|  |  | ADK |
|  |  | SLC9A1 |
|  |  | NEXN |
|  |  | CRYZ |
|  |  | TTI2 |
|  |  | TGOLN2 |
|  |  | DNAJB9 |
|  |  | MRPS27 |
|  |  | TNN |
|  |  | WBP2 |
|  |  | UBAP2L |
|  |  | MPZL3 |
|  |  | TTYH2 |
|  |  | AGRP |
|  |  | MIR181A1 |
|  |  | SMOC1 |
|  |  | MIR100 |
|  |  | GPR146 |
|  |  | TMEM131 |
|  |  | SORD |
|  |  | USP26 |
|  |  | EIF3C |
|  |  | TIMM8B |
|  |  | SCRN3 |
|  |  | INSYN2B |
|  |  | ACTN3A |
|  |  | TOGARAM1 |
|  |  | SIDT2 |
|  |  | TRAF1 |
|  |  | HSH2D |
|  |  | H1FX |
|  |  | SCAMP1 |
|  |  | ELF2 |
|  |  | TRIM32 |
|  |  | EID3 |
|  |  | PRKY |
|  |  | NPR2 |
|  |  | ANPEP |
|  |  | KRTAP2-3 |
|  |  | PRLR |
|  |  | ZNF569 |
|  |  | NKAPD1 |
|  |  | SNHG10 |
|  |  | PADI2 |
|  |  | ALPI |
|  |  | FTSJ1 |
|  |  | HSF1 |
|  |  | ZNF273 |
|  |  | FAT3 |
|  |  | BAP1 |
|  |  | GLRX5 |
|  |  | MIR3472 |
|  |  | PIP4K2C |
|  |  | LNX2 |
|  |  | CYLD |
|  |  | METTL7A |
|  |  | ITM2A |
|  |  | WTAP |
|  |  | DSG3 |
|  |  | BUB1B |
|  |  | ZNF180 |
|  |  | NFKBIB |
|  |  | SLC46A1 |
|  |  | PRRC2A |
|  |  | DKK1 |
|  |  | LINC00707 |
|  |  | STK35 |
|  |  | RRM1 |
|  |  | APOBEC3C |
|  |  | ABHD11 |
|  |  | RAD17 |
|  |  | INPP5D |
|  |  | KLHL29 |
|  |  | HJURP |
|  |  | BRD7P3 |
|  |  | ARID4A |
|  |  | MAT1A |
|  |  | CALM3 |
|  |  | TSPAN1 |
|  |  | ACIN1 |
|  |  | CMBL |
|  |  | LECT2 |
|  |  | SRSF2 |
|  |  | RAB30 |
|  |  | TMEM163 |
|  |  | GBF1 |
|  |  | FMO2 |
|  |  | IPO11 |
|  |  | UBTD2 |
|  |  | TCF3 |
|  |  | DIPK1A |
|  |  | ZSWIM7 |
|  |  | DEPTOR |
|  |  | SERPINE1 |
|  |  | CCDC88C |
|  |  | B4GALT3 |
|  |  | SPATA5 |
|  |  | BACH1 |
|  |  | GGNBP2 |
|  |  | ARC |
|  |  | NRDE2 |
|  |  | CUL9 |
|  |  | PRPF38A |
|  |  | PTAFR |
|  |  | CENPV |
|  |  | CREBRF |
|  |  | FAM122C |
|  |  | RSAD2 |
|  |  | CPTP |
|  |  | SNORA28 |
|  |  | TM7SF3 |
|  |  | OSCP1 |
|  |  | MLEC |
|  |  | KLC1 |
|  |  | GALE |
|  |  | PLLP |
|  |  | PDK1 |
|  |  | TBC1D10B |
|  |  | TRNQ |
|  |  | GAS-1 |
|  |  | ZNF337 |
|  |  | GXYLT1 |
|  |  | BCCIP |
|  |  | MEST |
|  |  | DYRK1A |
|  |  | IGFBP2B |
|  |  | DLD |
|  |  | CPA5 |
|  |  | CYP2C29 |
|  |  | TMEM30A |
|  |  | DENND4C |
|  |  | KIAA0319 |
|  |  | NEDD9 |
|  |  | PKM |
|  |  | ACTB1 |
|  |  | PSG6 |
|  |  | HSPA1B |
|  |  | PSMD13 |
|  |  | FCER2 |
|  |  | SLC39A11 |
|  |  | HDHD5 |
|  |  | CXCL5 |
|  |  | CDK19 |
|  |  | TRAPPC4 |
|  |  | BTBD7 |
|  |  | S1PR5 |
|  |  | GSTO1 |
|  |  | HNRNPA3 |
|  |  | LAPTM5 |
|  |  | SCNN1A |
|  |  | PPP1R1C |
|  |  | NOLC1 |
|  |  | UNC13C |
|  |  | P2RX4 |
|  |  | CCND2 |
|  |  | COX11 |
|  |  | PDZK1 |
|  |  | QSOX1 |
|  |  | ZNF443 |
|  |  | PCP4 |
|  |  | ZBED2 |
|  |  | SLC34A2 |
|  |  | FAM217B |
|  |  | NPTN |
|  |  | SART3 |
|  |  | UPP1 |
|  |  | FOXP1 |
|  |  | MIR362 |
|  |  | NDUFS5 |
|  |  | CISD1 |
|  |  | MLNR |
|  |  | PDLIM3 |
|  |  | VPS24 |
|  |  | 8-Mar |
|  |  | MRCL3 |
|  |  | GMNN |
|  |  | SNORD87 |
|  |  | TJP2 |
|  |  | SERINC1 |
|  |  | SCP2A |
|  |  | FDXR |
|  |  | SPCS2 |
|  |  | ATD4B |
|  |  | PAPLN |
|  |  | CSF2RA |
|  |  | MCRIP2 |
|  |  | PRKAB1 |
|  |  | RALGAPA1 |
|  |  | PSMD7 |
|  |  | CCDC121 |
|  |  | PPP1R3C |
|  |  | TMTC4 |
|  |  | TTC17 |
|  |  | ELP1 |
|  |  | PFN1 |
|  |  | ZNF654 |
|  |  | MICALL2 |
|  |  | ZNF253 |
|  |  | BCL3 |
|  |  | SLC16A8 |
|  |  | ITGB3 |
|  |  | SPATA13 |
|  |  | TCEA3 |
|  |  | BMPR1B |
|  |  | CLIP1 |
|  |  | TNFB |
|  |  | PDE8B |
|  |  | LTC4S |
|  |  | RUSC1-AS1 |
|  |  | CALHM6 |
|  |  | SEMA5A |
|  |  | SMARCA2 |
|  |  | TRNC |
|  |  | H2BFS |
|  |  | MORN2 |
|  |  | ZNF222 |
|  |  | BCL11A |
|  |  | INHBC |
|  |  | F11R |
|  |  | NAA25 |
|  |  | GSK3A |
|  |  | PI16 |
|  |  | WASH3P |
|  |  | CCDC198 |
|  |  | CEMIP2 |
|  |  | TXN |
|  |  | GPR87 |
|  |  | ACTA2 |
|  |  | PLAC8 |
|  |  | GPT2 |
|  |  | ANKRD1 |
|  |  | TIMM8A1 |
|  |  | DDR1 |
|  |  | RIPOR2 |
|  |  | LAMA4 |
|  |  | ALDOB |
|  |  | ICAL |
|  |  | DCC |
|  |  | RNF157 |
|  |  | CYP2D22 |
|  |  | TMEM185A |
|  |  | ZNF112 |
|  |  | ZNF436 |
|  |  | ZNF385A |
|  |  | IGFBP4 |
|  |  | UBE3C |
|  |  | TRIT1 |
|  |  | HOXC6 |
|  |  | PDSS2 |
|  |  | ATE1 |
|  |  | CLIC2 |
|  |  | RBFA |
|  |  | ZNFX1 |
|  |  | CCDC92 |
|  |  | KLF10 |
|  |  | DCTN5 |
|  |  | GRIM |
|  |  | TTPA |
|  |  | PSME3 |
|  |  | PHF10 |
|  |  | DBR1 |
|  |  | PEX5L |
|  |  | EPS15L1 |
|  |  | NME3 |
|  |  | HIST1H3A |
|  |  | BCAT1 |
|  |  | SLC2A9 |
|  |  | TMSB4X |
|  |  | CEBPB |
|  |  | ZNF398 |
|  |  | LRCH3 |
|  |  | VPS13C |
|  |  | HMGA1 |
|  |  | XIRP2 |
|  |  | CAPN7 |
|  |  | GEM |
|  |  | CCL3 |
|  |  | ANXA4 |
|  |  | DCP1B |
|  |  | UAP1 |
|  |  | KCNJ15 |
|  |  | MIR186 |
|  |  | TULP3 |
|  |  | RETSAT |
|  |  | NPL |
|  |  | FAM110B |
|  |  | CAST |
|  |  | GAB1 |
|  |  | ZNF557 |
|  |  | FAM81A |
|  |  | SNAI2 |
|  |  | KCNN4 |
|  |  | SCAF11 |
|  |  | ZMYM6 |
|  |  | CYP2J2 |
|  |  | ADGRA3 |
|  |  | HEATR3 |
|  |  | GIT2 |
|  |  | CCDC71 |
|  |  | TADA2B |
|  |  | ABHD6 |
|  |  | CANT1B |
|  |  | ATP6V0E1 |
|  |  | SLCO4C1 |
|  |  | TIFA |
|  |  | CCDC58 |
|  |  | FDPS |
|  |  | ZNF658 |
|  |  | NCOA4 |
|  |  | CNPY1 |
|  |  | CYP24A1 |
|  |  | AMD1 |
|  |  | PRKCA |
|  |  | TRIB2 |
|  |  | CCDC115 |
|  |  | COQ2 |
|  |  | TNS2 |
|  |  | CTC1 |
|  |  | ATP8B1 |
|  |  | HIPK1 |
|  |  | MAT2A |
|  |  | NDRG3 |
|  |  | TRIM8 |
|  |  | KAT6B |
|  |  | ZNF383 |
|  |  | JCHAIN |
|  |  | PEPD |
|  |  | SEC22A |
|  |  | GRIA2 |
|  |  | ANXA11 |
|  |  | CSF2RB |
|  |  | LMBR1 |
|  |  | IFI44 |
|  |  | UBXN2A |
|  |  | LANCL1 |
|  |  | PNPLA2 |
|  |  | BCKDHB |
|  |  | ERR |
|  |  | FOXO3A |
|  |  | EAF2 |
|  |  | VPS13B |
|  |  | USP3 |
|  |  | SPART |
|  |  | PLSCR3 |
|  |  | PSMB9 |
|  |  | CRISP1 |
|  |  | VSTM1 |
|  |  | SPOCD1 |
|  |  | SMCR8 |
|  |  | RPS6KB1 |
|  |  | ATP5MD |
|  |  | POLR3B |
|  |  | DHRS2 |
|  |  | RAB8A |
|  |  | RXFP2 |
|  |  | EXOC6 |
|  |  | DNAJC10 |
|  |  | HBA1 |
|  |  | RPS27 |
|  |  | ADIPOQ |
| **GeneCard** | **1108** | ELMO2 |
|  |  | KCNMA1 |
|  |  | PKNOX2 |
|  |  | MEFV |
|  |  | ABCB7 |
|  |  | GGT3P |
|  |  | NCF4 |
|  |  | RAB1B |
|  |  | CHD8 |
|  |  | NTRK1 |
|  |  | LRRC6 |
|  |  | MYO9B |
|  |  | EP300 |
|  |  | PRKCG |
|  |  | TBX10 |
|  |  | KCNA6 |
|  |  | MAX |
|  |  | GRM2 |
|  |  | MIR200B |
|  |  | RPL7A |
|  |  | HMX1 |
|  |  | EA3 |
|  |  | ITGA3 |
|  |  | ENSG00000224836 |
|  |  | ELANE |
|  |  | GTPBP1 |
|  |  | ISL1 |
|  |  | CD1A |
|  |  | FOXP3 |
|  |  | WDR45 |
|  |  | ENSG00000271238 |
|  |  | PYY |
|  |  | KCNC1 |
|  |  | MBS2 |
|  |  | CD38 |
|  |  | RIMBP3B |
|  |  | GYPC |
|  |  | LINC00328 |
|  |  | EMC1 |
|  |  | CDK1 |
|  |  | P2RX3 |
|  |  | TUBB |
|  |  | MYH6 |
|  |  | HCRTR2 |
|  |  | GMPPA |
|  |  | NME8 |
|  |  | FCN1 |
|  |  | CDCA4 |
|  |  | CARD8 |
|  |  | CELA1 |
|  |  | RSPH4A |
|  |  | POLG |
|  |  | RAG1 |
|  |  | ARSH |
|  |  | TACR1 |
|  |  | GNRHR |
|  |  | HTR3A |
|  |  | ZBED1 |
|  |  | POF1B |
|  |  | NCAM1 |
|  |  | IZUMO2 |
|  |  | CNE4 |
|  |  | FGF1 |
|  |  | LOC108281111 |
|  |  | COX10 |
|  |  | CIITA |
|  |  | NQO2 |
|  |  | SPATA31A7 |
|  |  | EPGN |
|  |  | OSR2 |
|  |  | ANKLE2 |
|  |  | SLC9A3 |
|  |  | PTPN2 |
|  |  | IL18BP |
|  |  | GFI1 |
|  |  | FHL1 |
|  |  | RPL4 |
|  |  | DLEC1 |
|  |  | KCNA4 |
|  |  | IL23R |
|  |  | SKAP2 |
|  |  | SCHIP1 |
|  |  | TAGAP |
|  |  | GOT2 |
|  |  | ZCCHC12 |
|  |  | RIC3 |
|  |  | GJB5 |
|  |  | PDGFRB |
|  |  | DTNB |
|  |  | GPR158 |
|  |  | WARS1 |
|  |  | MYF5 |
|  |  | AMELX |
|  |  | SLC2A10 |
|  |  | HCRTR1 |
|  |  | TAB2 |
|  |  | DDI2 |
|  |  | PGLYRP1 |
|  |  | RAB5A |
|  |  | ICA1L |
|  |  | CA9 |
|  |  | MIR491 |
|  |  | SLC29A3 |
|  |  | SDHD |
|  |  | CELIAC2 |
|  |  | TLR5 |
|  |  | LINC01194 |
|  |  | IGFBP2 |
|  |  | NOTCH2 |
|  |  | SIGLEC1 |
|  |  | CLEC4M |
|  |  | MGP |
|  |  | DVL1 |
|  |  | MAB21L2 |
|  |  | KRT74 |
|  |  | ITK |
|  |  | JAGN1 |
|  |  | PTPN4 |
|  |  | MAP6 |
|  |  | MAS1L |
|  |  | CDH11 |
|  |  | NRXN1 |
|  |  | DNAAF5 |
|  |  | PHYH |
|  |  | NIPA2 |
|  |  | GBX2 |
|  |  | BLK |
|  |  | HLA-DQA1 |
|  |  | ZMYND10 |
|  |  | SNW1 |
|  |  | SLAMF6 |
|  |  | CR1 |
|  |  | ADAD1 |
|  |  | FAM13A |
|  |  | TOR1A |
|  |  | PABPN1 |
|  |  | KIR2DS5 |
|  |  | ADGRF5 |
|  |  | KCNH1 |
|  |  | TBC1D24 |
|  |  | DGCR8 |
|  |  | CCL11 |
|  |  | FCGR2A |
|  |  | HTR1A |
|  |  | LALBA |
|  |  | F2RL2 |
|  |  | CRHR1 |
|  |  | MBS1 |
|  |  | DRC3 |
|  |  | F13A1 |
|  |  | FMR1 |
|  |  | CCDC117 |
|  |  | APELA |
|  |  | IFT172 |
|  |  | MIR222 |
|  |  | OCM2 |
|  |  | PNMA2 |
|  |  | SLC30A8 |
|  |  | ACR |
|  |  | NKX2-3 |
|  |  | GP1BB |
|  |  | OCLN |
|  |  | CNE-2 |
|  |  | ATP13A2 |
|  |  | NAGLU |
|  |  | PAX6 |
|  |  | IGKC |
|  |  | LOC102724770 |
|  |  | SNX18 |
|  |  | NETO1 |
|  |  | CXCL2 |
|  |  | CLDN3 |
|  |  | PUS3 |
|  |  | NKX3-1 |
|  |  | CELA3B |
|  |  | LOC100506321 |
|  |  | UFD1 |
|  |  | CD28 |
|  |  | SMARCA4 |
|  |  | MASP2 |
|  |  | CLPTM1L |
|  |  | SCT |
|  |  | SKI |
|  |  | KRT16 |
|  |  | ARHGAP29 |
|  |  | SCARA3 |
|  |  | CNE5 |
|  |  | PVALB |
|  |  | MIR1306 |
|  |  | GSC2 |
|  |  | PRDM10 |
|  |  | CDC45 |
|  |  | TBX3 |
|  |  | TAZ |
|  |  | SCIMP |
|  |  | GAS2L2 |
|  |  | CD247 |
|  |  | SOX14 |
|  |  | SPTBN2 |
|  |  | PTH |
|  |  | PTLS |
|  |  | BARX1 |
|  |  | LOC106020709 |
|  |  | GRIK3 |
|  |  | ADIRF |
|  |  | STPG2 |
|  |  | MED15 |
|  |  | ITGAX |
|  |  | CASR |
|  |  | MT-CO1 |
|  |  | PRPH |
|  |  | LRP12 |
|  |  | FGF10 |
|  |  | TSPEAR |
|  |  | SGSH |
|  |  | TNFRSF18 |
|  |  | PLG |
|  |  | TYR |
|  |  | CNE6 |
|  |  | VCAN |
|  |  | POTEB2 |
|  |  | PANK2 |
|  |  | RNH1 |
|  |  | JUP |
|  |  | LINC01193 |
|  |  | ACP1 |
|  |  | KIAA1586 |
|  |  | RAB11FIP5 |
|  |  | CD84 |
|  |  | GATA2 |
|  |  | TCF20 |
|  |  | MIR199A1 |
|  |  | KCNQ3 |
|  |  | SHOX |
|  |  | CAV2 |
|  |  | S100A7 |
|  |  | TBX22 |
|  |  | HPSE |
|  |  | DNAAF6 |
|  |  | RFFL |
|  |  | MIR196A2 |
|  |  | IL9 |
|  |  | MKX |
|  |  | ATP2B3 |
|  |  | RPN2 |
|  |  | CRTC1 |
|  |  | C4BPA |
|  |  | DCN |
|  |  | COL11A1 |
|  |  | S100A12 |
|  |  | VAMP7 |
|  |  | ACTN1 |
|  |  | HLA-C |
|  |  | ITGAE |
|  |  | FUT2 |
|  |  | FZD4 |
|  |  | GALNS |
|  |  | SERPINB3 |
|  |  | HLA-DRB3 |
|  |  | SYNM |
|  |  | FLT4 |
|  |  | MIR483 |
|  |  | ADAMTSL1 |
|  |  | GJB4 |
|  |  | DTNA |
|  |  | AMHR2 |
|  |  | CTTN |
|  |  | ITGAL |
|  |  | NDUFA5 |
|  |  | CH25H |
|  |  | SDHA |
|  |  | FAM230B |
|  |  | DRG2 |
|  |  | LOC107988023 |
|  |  | DARS2 |
|  |  | XRCC2 |
|  |  | ATOX1 |
|  |  | BCL2L12 |
|  |  | ZAP70 |
|  |  | SLC6A2 |
|  |  | LPP |
|  |  | MIR193B |
|  |  | MIR146A |
|  |  | TGFB2 |
|  |  | KLK6 |
|  |  | LZTR1 |
|  |  | ARVCF |
|  |  | NBEAL1 |
|  |  | LOC108745275 |
|  |  | HOXC13 |
|  |  | NLRC4 |
|  |  | RNF170 |
|  |  | GNAI3 |
|  |  | CD4 |
|  |  | DSP |
|  |  | DGCR9 |
|  |  | PTK2B |
|  |  | SDHB |
|  |  | NUDT10 |
|  |  | MBL2 |
|  |  | MFSD6 |
|  |  | SH2D1A |
|  |  | MUSK |
|  |  | STRADB |
|  |  | CDK15 |
|  |  | IL21-AS1 |
|  |  | RARS1 |
|  |  | BMPR1A |
|  |  | TMEM127 |
|  |  | CCR9 |
|  |  | SSX2 |
|  |  | PRDM9 |
|  |  | TBX5 |
|  |  | GJB6 |
|  |  | KRT13 |
|  |  | DRD5 |
|  |  | PAFAH1B1 |
|  |  | H2AC18 |
|  |  | RAPH1 |
|  |  | DOCK8 |
|  |  | RBM24 |
|  |  | ATP4A |
|  |  | PI4KA |
|  |  | POU2AF1 |
|  |  | VWA8 |
|  |  | CYP21A2 |
|  |  | WIZ |
|  |  | MGAT1 |
|  |  | MIR502 |
|  |  | PARVA |
|  |  | RNF34 |
|  |  | NKRF |
|  |  | MBTPS1 |
|  |  | SECISBP2 |
|  |  | XRCC6 |
|  |  | GABRR2 |
|  |  | PAX9 |
|  |  | MIR1269B |
|  |  | ABO |
|  |  | RIPPLY3 |
|  |  | STAT4 |
|  |  | EFTUD2 |
|  |  | SCN4A |
|  |  | MYOT |
|  |  | HIRA |
|  |  | SMCR5 |
|  |  | ANTXR2 |
|  |  | TAPBP |
|  |  | RET |
|  |  | MID1 |
|  |  | ALDH1A2 |
|  |  | GABRG1 |
|  |  | MIR574 |
|  |  | CD58 |
|  |  | CCN6 |
|  |  | IFNL3 |
|  |  | GRHL2 |
|  |  | EXOSC9 |
|  |  | KLRC2 |
|  |  | VGLL1 |
|  |  | PLAGL1 |
|  |  | MAGT1 |
|  |  | LLGL1 |
|  |  | FTL |
|  |  | SCUBE1 |
|  |  | ACCS |
|  |  | FAM230A |
|  |  | CYP2A6 |
|  |  | LOC109245079 |
|  |  | EYA1 |
|  |  | PITX2 |
|  |  | TRPV2 |
|  |  | LOC108745276 |
|  |  | TEX49 |
|  |  | FA2H |
|  |  | MIR363 |
|  |  | MAPRE3 |
|  |  | FKBP10 |
|  |  | SLC1A2 |
|  |  | INVS |
|  |  | NTF4 |
|  |  | TLR8 |
|  |  | LZTS1 |
|  |  | GABRB2 |
|  |  | CD27 |
|  |  | YDJC |
|  |  | ORAI1 |
|  |  | TPH1 |
|  |  | NEK1 |
|  |  | MPP2 |
|  |  | ENSG00000281530 |
|  |  | PTER |
|  |  | WAS |
|  |  | ZBTB7A |
|  |  | LOC106020712 |
|  |  | ARRB1 |
|  |  | DCAF7 |
|  |  | TCTEX1D2 |
|  |  | WDR60 |
|  |  | CAMP |
|  |  | LINC01191 |
|  |  | CYP2A13 |
|  |  | PTX3 |
|  |  | SCGB3A1 |
|  |  | S100A1 |
|  |  | NAGS |
|  |  | KERA |
|  |  | BCL2L13 |
|  |  | DYNC2H1 |
|  |  | MTUS1 |
|  |  | CNE7 |
|  |  | GABRA6 |
|  |  | MMEL1 |
|  |  | MGMT |
|  |  | HOXA7 |
|  |  | RNF138 |
|  |  | HSFX2 |
|  |  | MT-ND1 |
|  |  | CNE-5 |
|  |  | LIX1 |
|  |  | KPNA5 |
|  |  | CELIAC7 |
|  |  | CHAD |
|  |  | FGF14 |
|  |  | POLB |
|  |  | TOR1B |
|  |  | GAPT |
|  |  | UBASH3A |
|  |  | LOC110806262 |
|  |  | TAS2R16 |
|  |  | IRF4 |
|  |  | PDPN |
|  |  | MIR423 |
|  |  | NAPA |
|  |  | KRT14 |
|  |  | IL1RAPL2 |
|  |  | GPR65 |
|  |  | STXBP2 |
|  |  | DEDD |
|  |  | SEPTIN5 |
|  |  | IFITM3 |
|  |  | VPS37C |
|  |  | CLDN5 |
|  |  | MYOM2 |
|  |  | STX1A |
|  |  | BMPR2 |
|  |  | MSH5 |
|  |  | GRIK1 |
|  |  | NOD1 |
|  |  | IL12RB2 |
|  |  | PSORS1C1 |
|  |  | SEC24C |
|  |  | AXIN2 |
|  |  | TAF1 |
|  |  | MIR200A |
|  |  | PIK3CA |
|  |  | LINC01546 |
|  |  | KDM4C |
|  |  | ERBB3 |
|  |  | CELIAC8 |
|  |  | GSX1 |
|  |  | SLC1A1 |
|  |  | PMP2 |
|  |  | TGM6 |
|  |  | CCDC65 |
|  |  | WDR35 |
|  |  | KCNQ2 |
|  |  | GRM5 |
|  |  | FLACC1 |
|  |  | CXCL12 |
|  |  | NTN1 |
|  |  | OPRD1 |
|  |  | C19orf12 |
|  |  | MBS3 |
|  |  | NGLY1 |
|  |  | CD63 |
|  |  | LGI1 |
|  |  | KIR3DS1 |
|  |  | PRKX-AS1 |
|  |  | TSG101 |
|  |  | TNFRSF1B |
|  |  | CACNA1S |
|  |  | WNT2B |
|  |  | LOC109136576 |
|  |  | USB1 |
|  |  | GPN1 |
|  |  | CNTNAP2 |
|  |  | FRK |
|  |  | PAX8 |
|  |  | IKBKE |
|  |  | MRPL40 |
|  |  | EDEM1 |
|  |  | ADH1A |
|  |  | CFTR |
|  |  | GSTM3 |
|  |  | LOXL3 |
|  |  | LOC108021842 |
|  |  | CD48 |
|  |  | DGCR6 |
|  |  | COL17A1 |
|  |  | DMAP1 |
|  |  | IFNA4 |
|  |  | NEUROG1 |
|  |  | DGCR6L |
|  |  | MATN2 |
|  |  | TGFBR2 |
|  |  | DGCR12 |
|  |  | TLR9 |
|  |  | MSLN |
|  |  | FGF3 |
|  |  | FOXN1 |
|  |  | CLEC16A |
|  |  | NOG |
|  |  | PPP2R2B |
|  |  | RBM8A |
|  |  | SCUBE2 |
|  |  | KCNB1 |
|  |  | DDX58 |
|  |  | LOC106020711 |
|  |  | RPGR |
|  |  | USP22 |
|  |  | FABP12 |
|  |  | CYP2B6 |
|  |  | UNC119 |
|  |  | MIR342 |
|  |  | KRT5 |
|  |  | SIX1 |
|  |  | MIR371A |
|  |  | CARF |
|  |  | GP5 |
|  |  | MIR874 |
|  |  | CSN2 |
|  |  | HSD11B1 |
|  |  | KITLG |
|  |  | SETX |
|  |  | ADAM22 |
|  |  | VTI1B |
|  |  | PYGM |
|  |  | PRAME |
|  |  | MEX3C |
|  |  | ATG16L1 |
|  |  | TRA |
|  |  | KRBOX4 |
|  |  | HSFX1 |
|  |  | PRRT2 |
|  |  | LINC00293 |
|  |  | ESPN |
|  |  | PAM16 |
|  |  | HOXB2 |
|  |  | CRKL |
|  |  | DVL1P1 |
|  |  | CDKN3 |
|  |  | HEYL |
|  |  | APPL1 |
|  |  | OCA2 |
|  |  | PDYN |
|  |  | NPC1 |
|  |  | RASSF1 |
|  |  | PITPNB |
|  |  | MIR649 |
|  |  | IL7 |
|  |  | GATA4 |
|  |  | CAMK2N2 |
|  |  | CEACAM3 |
|  |  | HBA2 |
|  |  | CNE8 |
|  |  | HAPLN1 |
|  |  | MIR490 |
|  |  | RAB7A |
|  |  | KANSL1-AS1 |
|  |  | HMSD |
|  |  | IFT52 |
|  |  | PSTPIP1 |
|  |  | CNTNAP1 |
|  |  | BYSL |
|  |  | ARRB2 |
|  |  | IGF1 |
|  |  | NKX2-5 |
|  |  | UBB |
|  |  | SPECC1L |
|  |  | HLA-DRB5 |
|  |  | B3GAT1 |
|  |  | PCOLCE |
|  |  | LBX1 |
|  |  | OXT |
|  |  | TANGO2 |
|  |  | MIR132 |
|  |  | KRT1 |
|  |  | IMPDH2 |
|  |  | MT-ATP6 |
|  |  | CNE9 |
|  |  | CHRNA5 |
|  |  | HTR7 |
|  |  | GLE1 |
|  |  | EVI2A |
|  |  | RIBC1 |
|  |  | AIRE |
|  |  | CELIAC11 |
|  |  | FOXA2 |
|  |  | MEIS2 |
|  |  | NFKB2 |
|  |  | IFT140 |
|  |  | ERLIN2 |
|  |  | TMEM117 |
|  |  | WDR19 |
|  |  | EDNRA |
|  |  | MIR9-1 |
|  |  | LINC00381 |
|  |  | POGZ |
|  |  | GHRL |
|  |  | IRF3 |
|  |  | LTBP1 |
|  |  | FGF8 |
|  |  | EPX |
|  |  | WNT3 |
|  |  | CFAP298 |
|  |  | OPRK1 |
|  |  | NTAN1 |
|  |  | LOC108684022 |
|  |  | CD99 |
|  |  | MEG8 |
|  |  | RARB |
|  |  | NKTR |
|  |  | FXN |
|  |  | SPG11 |
|  |  | AP3B1 |
|  |  | KIAA0586 |
|  |  | DNAAF1 |
|  |  | TNFSF12 |
|  |  | CHRNA2 |
|  |  | RSPO2 |
|  |  | TTC21B |
|  |  | TPMT |
|  |  | MIR144 |
|  |  | MEGF10 |
|  |  | SULT1A2 |
|  |  | GAA |
|  |  | MYOD1 |
|  |  | KLRC1 |
|  |  | SCN1A |
|  |  | WNT3A |
|  |  | MIR29B1 |
|  |  | GABBR1 |
|  |  | DISC1 |
|  |  | USP4 |
|  |  | CAPN6 |
|  |  | CLTCL1 |
|  |  | CHAF1A |
|  |  | DEFB4A |
|  |  | VWA2 |
|  |  | FTH1 |
|  |  | COG4 |
|  |  | IKBKG |
|  |  | OR13H1 |
|  |  | EDAR |
|  |  | GNRH1 |
|  |  | FYN |
|  |  | KCND3 |
|  |  | ITGB1 |
|  |  | BVES |
|  |  | PSMC4 |
|  |  | TTC12 |
|  |  | NAIP |
|  |  | UCA1 |
|  |  | FHIT |
|  |  | HAND2 |
|  |  | HRCT1 |
|  |  | EA7 |
|  |  | DGCR5 |
|  |  | WNT5A |
|  |  | RANBP1 |
|  |  | ENO2 |
|  |  | BMP7 |
|  |  | CFHR1 |
|  |  | SLC7A4 |
|  |  | LOC108449888 |
|  |  | NELFCD |
|  |  | CELIAC5 |
|  |  | MIR183 |
|  |  | ADH1C |
|  |  | FCGR3A |
|  |  | GAS8 |
|  |  | PSMD2 |
|  |  | ATXN2 |
|  |  | LAMC3 |
|  |  | GABRG3 |
|  |  | EDA2R |
|  |  | PTPN14 |
|  |  | EPHB3 |
|  |  | FAM53A |
|  |  | GAP43 |
|  |  | TMC6 |
|  |  | MDH2 |
|  |  | CFLAR-AS1 |
|  |  | APEX1 |
|  |  | ACKR2 |
|  |  | SCARF2 |
|  |  | NATD1 |
|  |  | TNFRSF8 |
|  |  | NLGN3 |
|  |  | LOC108449897 |
|  |  | GP2 |
|  |  | CHIT1 |
|  |  | PLOD2 |
|  |  | ABT1 |
|  |  | LOC108663985 |
|  |  | PRNT |
|  |  | CTSF |
|  |  | ZNF74 |
|  |  | KIAA0556 |
|  |  | MMP12 |
|  |  | TNFSF13 |
|  |  | GABRA1 |
|  |  | METAP1 |
|  |  | EPCAM |
|  |  | PLXND1 |
|  |  | MIR338 |
|  |  | TEAD4 |
|  |  | ALS2 |
|  |  | MIR335 |
|  |  | KIAA1109 |
|  |  | CCT7 |
|  |  | MMP11 |
|  |  | SNAP29 |
|  |  | CDH3 |
|  |  | HOXB1 |
|  |  | MTUS2 |
|  |  | SLC1A6 |
|  |  | NECTIN1 |
|  |  | HOTAIR |
|  |  | FCGRT |
|  |  | CCR6 |
|  |  | COL11A2 |
|  |  | GATA3 |
|  |  | HHEX |
|  |  | CTNS |
|  |  | CTNNBIP1 |
|  |  | BRPF1 |
|  |  | ACP5 |
|  |  | TBCE |
|  |  | PLCB4 |
|  |  | SAG |
|  |  | HOXA2 |
|  |  | AASS |
|  |  | VPREB1 |
|  |  | NAE1 |
|  |  | KDF1 |
|  |  | NRL |
|  |  | SDHAF2 |
|  |  | SRPX2 |
|  |  | CTLA4 |
|  |  | TLR7 |
|  |  | PDGFA |
|  |  | SELPLG |
|  |  | DEFA1 |
|  |  | HLA-DPB1 |
|  |  | CTSE |
|  |  | TUSC3 |
|  |  | SLC25A11 |
|  |  | EMP3 |
|  |  | MAPK8 |
|  |  | ESS2 |
|  |  | SRP54 |
|  |  | CHRNB3 |
|  |  | HLA-DQA2 |
|  |  | MIR143 |
|  |  | FCGR3B |
|  |  | NFE2L1 |
|  |  | FAM3D-AS1 |
|  |  | PATE1 |
|  |  | ANKK1 |
|  |  | COPS3 |
|  |  | OR1C1 |
|  |  | IL17RA |
|  |  | PTPRN |
|  |  | SRC |
|  |  | MIR30E |
|  |  | TFAP2A |
|  |  | LOC109245078 |
|  |  | SGCD |
|  |  | TWIST2 |
|  |  | GFER |
|  |  | CD209 |
|  |  | SBDS |
|  |  | GABRB3 |
|  |  | HOXD3 |
|  |  | DDX1 |
|  |  | SPAG1 |
|  |  | MIR373 |
|  |  | TAS2R13 |
|  |  | STK11 |
|  |  | LOXL4 |
|  |  | MIR455 |
|  |  | KIR2DS1 |
|  |  | PDCD1 |
|  |  | DYNC2LI1 |
|  |  | ATXN1 |
|  |  | CNOT8 |
|  |  | GABRG2 |
|  |  | CHRNA3 |
|  |  | F5 |
|  |  | DCANP1 |
|  |  | ENSG00000235105 |
|  |  | LOC106020710 |
|  |  | NRXN3 |
|  |  | FCGR1A |
|  |  | LAP3 |
|  |  | IMPDH1 |
|  |  | ICOS |
|  |  | NKX2-6 |
|  |  | UGT1A |
|  |  | WRAP53 |
|  |  | PTPRU |
|  |  | LOC116309126 |
|  |  | UBE3A |
|  |  | SHOX2 |
|  |  | TSSK2 |
|  |  | TOLLIP |
|  |  | NOD2 |
|  |  | IL21 |
|  |  | TPH2 |
|  |  | AKR1A1 |
|  |  | ENGASE |
|  |  | P2RX7 |
|  |  | DHX58 |
|  |  | LINC00540 |
|  |  | IL18R1 |
|  |  | TBX6 |
|  |  | GLB1 |
|  |  | HCRT |
|  |  | CELIAC6 |
|  |  | BACH2 |
|  |  | CELIAC10 |
|  |  | NME1 |
|  |  | EEC2 |
|  |  | LACTB |
|  |  | SRRM2 |
|  |  | DES-LCR |
|  |  | TCOF1 |
|  |  | GJA5 |
|  |  | PYCARD |
|  |  | DGCR10 |
|  |  | TMEM54 |
|  |  | EYA2 |
|  |  | DCAF17 |
|  |  | MAGEA4 |
|  |  | VAMP2 |
|  |  | NBN |
|  |  | PLAU |
|  |  | ATP1A3 |
|  |  | DELEC1 |
|  |  | MATR3 |
|  |  | RFX5 |
|  |  | TXNDC15 |
|  |  | PKP1 |
|  |  | CELIAC9 |
|  |  | NRP1 |
|  |  | MIR30A |
|  |  | GRK6 |
|  |  | HPS6 |
|  |  | CARD11 |
|  |  | THRIL |
|  |  | PHF3 |
|  |  | KCNA2 |
|  |  | IFNA2 |
|  |  | LOC644462 |
|  |  | HLA-E |
|  |  | EVC2 |
|  |  | LMLN |
|  |  | MUC1 |
|  |  | TEAD3 |
|  |  | AICDA |
|  |  | TLR6 |
|  |  | STIM1 |
|  |  | TBX2 |
|  |  | TMEM237 |
|  |  | IFITM5 |
|  |  | SDHAF3 |
|  |  | RAI14 |
|  |  | TUBA3C |
|  |  | GCM2 |
|  |  | HOXB3 |
|  |  | PIGR |
|  |  | CDC25C |
|  |  | LOC108021843 |
|  |  | ARF1 |
|  |  | KRT3 |
|  |  | CDX2 |
|  |  | MIR615 |
|  |  | CHGA |
|  |  | CACNA1B |
|  |  | IFT80 |
|  |  | GNB1L |
|  |  | TTBK2 |
|  |  | HLA-DQB1 |
|  |  | MT-CYB |
|  |  | ECM1 |
|  |  | CHRNB4 |
|  |  | OGG1 |
|  |  | HOXA3 |
|  |  | RPS8 |
|  |  | DLX4 |
|  |  | MSX2 |
|  |  | USP9X |
|  |  | DYTN |
|  |  | FCN2 |
|  |  | COG2 |
|  |  | CELIAC13 |
|  |  | HTR3B |
|  |  | FAF1 |
|  |  | HMX2 |
|  |  | TEAD2 |
|  |  | GABRB1 |
|  |  | EEC1 |
|  |  | KLRK1 |
|  |  | LYST |
|  |  | ADH7 |
|  |  | HLA-DPA1 |
|  |  | PLET1 |
|  |  | RSPH1 |
|  |  | NALCN |
|  |  | UNC13D |
|  |  | SOX3 |
|  |  | TMC8 |
|  |  | CALR |
|  |  | MMACHC |
|  |  | CD7 |
|  |  | MIR3618 |
|  |  | STX11 |
|  |  | KCNC3 |
|  |  | DNAJA1 |
|  |  | SCGB1D4 |
|  |  | ARMC4 |
|  |  | POMK |
|  |  | ADAM10 |
|  |  | SMCP |
|  |  | TBX1 |
|  |  | HIRIP3 |
|  |  | ACO1 |
|  |  | OPRL1 |
|  |  | LIG4 |
|  |  | BMP4 |
|  |  | RNF168 |
|  |  | TJP1 |
|  |  | CCKBR |
|  |  | CCK |
|  |  | SIT1 |
|  |  | SEC22B |
|  |  | GLI3 |
|  |  | TWNK |
|  |  | LHPP |
|  |  | NTRK3 |
|  |  | FBN1 |
|  |  | HYDIN |
|  |  | TNFRSF13C |
|  |  | DHRS3 |
|  |  | DROSHA |
|  |  | PUS10 |
|  |  | RFXANK |
|  |  | COASY |
|  |  | ADA |
|  |  | ERVW-1 |
|  |  | ANXA5 |
|  |  | CCDC114 |
|  |  | DGCR11 |
|  |  | MIR744 |
|  |  | KIAA0040 |
|  |  | CPA6 |
|  |  | MIR18B |
|  |  | BLOC1S1 |
|  |  | SLC37A4 |
|  |  | TGM1 |
|  |  | TMBIM4 |
|  |  | TSPOAP1 |
|  |  | MYD88 |
|  |  | PML |
|  |  | EBAG9 |
|  |  | DGCR |
|  |  | AFF2 |
|  |  | ZDHHC8 |
|  |  | PLXNA2 |
|  |  | CD244 |
|  |  | CD24 |
|  |  | NSF |
|  |  | ATP1A2 |
|  |  | TG |
|  |  | IFNA17 |
|  |  | LOXL2 |
|  |  | HOMER2 |
|  |  | BCL2L2-PABPN1 |
|  |  | MIR486-1 |
|  |  | CFI |
|  |  | ZNF699 |
|  |  | ETFDH |
|  |  | IL37 |
|  |  | KREMEN1 |
|  |  | GP9 |
|  |  | DMBT1 |
|  |  | NUTM1 |
|  |  | TTC28 |
|  |  | TSPO |
|  |  | FGF9 |
|  |  | CACNA1A |
|  |  | MIR196A1 |
|  |  | VGLL2 |
|  |  | GRIN1 |
|  |  | SCN2A |
|  |  | XPA |
|  |  | GNPTAB |
|  |  | TRPM1 |
|  |  | CCKAR |
|  |  | HOXA@ |
|  |  | BFAR |
|  |  | SLC30A10 |
|  |  | PRTN3 |
|  |  | NPBWR1 |
|  |  | GJB2 |
|  |  | PRKD1 |
|  |  | SIX4 |
|  |  | CD19 |
|  |  | DLX5 |
|  |  | PRR7 |
|  |  | IGLON5 |
|  |  | CTSL |
|  |  | TPO |
|  |  | LTF |
|  |  | TNIP2 |
|  |  | CD81 |
|  |  | MYH7 |
|  |  | SH2B3 |
|  |  | PTS |
|  |  | ADCY6 |
|  |  | GDF5 |
|  |  | TBX20 |
|  |  | GOLIM4 |
|  |  | CHRM2 |
|  |  | MAGEA1 |
|  |  | GABRA4 |
|  |  | LOC108021844 |
|  |  | MIR139 |
|  |  | KCNC4 |
|  |  | CYP26B1 |
|  |  | MIR29C |
|  |  | AQP5 |
|  |  | MPDZ |
|  |  | CCL21 |
|  |  | HFE |
|  |  | MIR659 |
|  |  | SMN1 |
|  |  | EDARADD |
|  |  | CR2 |
|  |  | IFT81 |
|  |  | CHRD |
|  |  | IFNA1 |
|  |  | ADH1B |
|  |  | SARDH |
|  |  | PPFIA1 |
|  |  | HBS1L |
|  |  | JAK3 |
|  |  | HEY2 |
|  |  | ATP12A |
|  |  | UGT2B11 |
|  |  | HMX3 |
|  |  | CHERP |
|  |  | FCN3 |
|  |  | NPTX1 |
|  |  | CLEC4G |
|  |  | SCN8A |
|  |  | RAB27A |
|  |  | PAX3 |
|  |  | CCL25 |
|  |  | PKD2L1 |
|  |  | ADH6 |
|  |  | F12 |
|  |  | STAC3 |
|  |  | SPAG7 |
|  |  | HKDC1 |
|  |  | EA8 |
|  |  | MIR1307 |
|  |  | PARD3B |
|  |  | RAG2 |
|  |  | PMM2 |
|  |  | KRT85 |
|  |  | CELIAC12 |
|  |  | IRAK4 |
|  |  | PABPC1P2 |
|  |  | NRG1 |
|  |  | DDX41 |
|  |  | DGCR2 |
|  |  | TAOK1 |
|  |  | VSIG10 |
|  |  | DNAAF4 |
|  |  | JPH3 |
|  |  | CASK |
|  |  | HSPG2 |
|  |  | LRPAP1 |
|  |  | IGHE |
|  |  | C2CD6 |
|  |  | PTPRD-AS2 |
|  |  | DGS2 |
|  |  | KLRD1 |
|  |  | VHL |
|  |  | PCNT |
|  |  | ERCC2 |
|  |  | DCLRE1C |
|  |  | XPO5 |
|  |  | TOP1 |
|  |  | CTSC |
