# Supplementary material for: Network Pharmacology Identifies the Mechanisms of Sang-Xing-Zhi-Ke-Fang against Pharyngitis
Source: Evid Based Complement Alternat Med. 2020 Oct 12;2020:2421916. doi: 10.1155/2020/2421916 (PMC7576344; doi:10.1155/2020/2421916)
Supplement: Supplementary Materials — Table S1: 102 bioactive compounds obtained and screened out from TCMSP, BATMAN-TCM, and literature. Table S2: 886 targets of bioactive compounds collected using TCMSP and UniProt. Table S3: targets related to pharyngitis, including 5150 targets from CTD and 1803 targets from GeneCards with 695 targets duplicated. Table S4: 387 overlapping targets related to 19 bioactive compounds. Table S5: the results of topological features of the PPI network, including the values of topological features of 354 targets, while the other 33 targets were unrelated to each other target in the network. Table S6: the results of KEGG pathway enrichment, including 43 KEGG pathways were recognized as P < 0.05 with 28 pathways being recognized as P < 0.01. [file 2421916.f1.zip › Supplementary materials/Suppplementary Table S5.docx]

| NO. | Target name | Degree Centrality | Betweenness Centrality | Closeness Centrality |
| --- | --- | --- | --- | --- |
| 1 | ALB | 132 | 0.10194051 | 0.57963875 |
| 2 | SREBF1 | 101 | 0.04648343 | 0.54058193 |
| 3 | APOB | 88 | 0.02798912 | 0.52373887 |
| 4 | APOE | 87 | 0.03066411 | 0.52765321 |
| 5 | PPARG | 84 | 0.02496889 | 0.53082707 |
| 6 | MAPK3 | 82 | 0.03803816 | 0.52923538 |
| 7 | HMGCR | 79 | 0.02839721 | 0.51911765 |
| 8 | EGF | 77 | 0.0326961 | 0.51683748 |
| 9 | LDLR | 74 | 0.01786479 | 0.5 |
| 10 | FASN | 74 | 0.01620772 | 0.502849 |
| 11 | PPARA | 74 | 0.01758367 | 0.5130814 |
| 12 | PTGS2 | 73 | 0.03636725 | 0.50573066 |
| 13 | SREBF2 | 72 | 0.02642683 | 0.4862259 |
| 14 | APP | 68 | 0.02026671 | 0.50500715 |
| 15 | LEP | 67 | 0.01366234 | 0.50864553 |
| 16 | ABCA1 | 66 | 0.02050839 | 0.49509116 |
| 17 | SCD | 66 | 0.01940855 | 0.48891967 |
| 18 | APOA1 | 65 | 0.0104694 | 0.48891967 |
| 19 | CAT | 59 | 0.0231604 | 0.50070922 |
| 20 | ADIPOQ | 59 | 0.01236963 | 0.49788434 |
| 21 | CYP3A4 | 59 | 0.01670894 | 0.48356164 |
| 22 | ESR1 | 58 | 0.0240653 | 0.49509116 |
| 23 | DGAT1 | 54 | 0.00895186 | 0.47446237 |
| 24 | CYP2B6 | 53 | 0.01261496 | 0.48422497 |
| 25 | APOC3 | 51 | 0.00521994 | 0.45844156 |
| 26 | ACACA | 51 | 0.0040295 | 0.47638327 |
| 27 | CREB1 | 51 | 0.01442992 | 0.49164345 |
| 28 | SIRT1 | 50 | 0.01736748 | 0.49301676 |
| 29 | MAPK14 | 48 | 0.00877424 | 0.47702703 |
| 30 | FABP1 | 48 | 0.01145724 | 0.46693122 |
| 31 | CAV1 | 48 | 0.01458166 | 0.48092643 |
| 32 | SQLE | 47 | 0.00778386 | 0.44853875 |
| 33 | AGT | 47 | 0.00760543 | 0.47510094 |
| 34 | CLU | 47 | 0.00773464 | 0.45844156 |
| 35 | LIPE | 47 | 0.00794991 | 0.47638327 |
| 36 | FADS1 | 47 | 0.00692009 | 0.45607235 |
| 37 | LIPC | 46 | 0.00999712 | 0.44968153 |
| 38 | CYP2E1 | 46 | 0.01342474 | 0.4738255 |
| 39 | IL4 | 45 | 0.00870765 | 0.47192513 |
| 40 | ACOX1 | 45 | 0.0035187 | 0.45784695 |
| 41 | HMGCS1 | 44 | 0.00325402 | 0.44853875 |
| 42 | ACLY | 44 | 0.01071917 | 0.46083551 |
| 43 | CYP19A1 | 44 | 0.01030837 | 0.47255689 |
| 44 | CYP2C9 | 44 | 0.00700349 | 0.46754967 |
| 45 | SCARB1 | 44 | 0.00915057 | 0.47003995 |
| 46 | FDFT1 | 43 | 0.00425437 | 0.45140665 |
| 47 | DGAT2 | 43 | 0.00166345 | 0.44291092 |
| 48 | APOA4 | 43 | 0.00578781 | 0.45314506 |
| 49 | CD44 | 43 | 0.01055526 | 0.46816976 |
| 50 | CYP51A1 | 41 | 0.00425467 | 0.44180225 |
| 51 | HSD3B1 | 41 | 0.00690686 | 0.44180225 |
| 52 | DHCR7 | 41 | 0.00378824 | 0.42891859 |
| 53 | PON1 | 41 | 0.00478681 | 0.46693122 |
| 54 | LSS | 40 | 0.00646986 | 0.44570707 |
| 55 | ACE | 40 | 0.00407366 | 0.47255689 |
| 56 | ABCG2 | 40 | 0.02206365 | 0.46816976 |
| 57 | F2 | 39 | 0.00736964 | 0.47319035 |
| 58 | KRAS | 39 | 0.00749875 | 0.46143791 |
| 59 | LCAT | 39 | 0.00395196 | 0.46204188 |
| 60 | NR3C1 | 39 | 0.00692536 | 0.47003995 |
| 61 | FABP4 | 38 | 0.0045635 | 0.44796954 |
| 62 | ABCB1 | 38 | 0.01106088 | 0.46693122 |
| 63 | HNF4A | 38 | 0.01122196 | 0.47574124 |
| 64 | HSD17B6 | 37 | 0.00560882 | 0.45548387 |
| 65 | NR1H3 | 37 | 0.00190687 | 0.44402516 |
| 66 | INSIG1 | 37 | 0.00236122 | 0.42996346 |
| 67 | POR | 37 | 0.01372369 | 0.4192399 |
| 68 | MTTP | 36 | 0.0018486 | 0.45083014 |
| 69 | CETP | 36 | 0.0013625 | 0.45372751 |
| 70 | SC5D | 36 | 0.00196807 | 0.42326139 |
| 71 | CYP2C19 | 36 | 0.00306847 | 0.44968153 |
| 72 | CYP1A1 | 36 | 0.00390879 | 0.45725389 |
| 73 | G6PC | 36 | 0.00849248 | 0.47319035 |
| 74 | PLA2G1B | 36 | 0.00413761 | 0.45725389 |
| 75 | ABCC2 | 36 | 0.01087837 | 0.44346734 |
| 76 | GPAM | 36 | 0.00459497 | 0.43206854 |
| 77 | MSMO1 | 35 | 0.00190003 | 0.42787879 |
| 78 | CYP1A2 | 35 | 0.00291643 | 0.44235589 |
| 79 | FDPS | 34 | 0.00246037 | 0.43154034 |
| 80 | RXRA | 34 | 0.00356788 | 0.46325459 |
| 81 | TGFB1 | 34 | 0.00269969 | 0.44740177 |
| 82 | MVK | 34 | 0.00234987 | 0.42839806 |
| 83 | EDN1 | 34 | 0.00203124 | 0.45372751 |
| 84 | ABCG1 | 34 | 0.0034198 | 0.45489691 |
| 85 | APOC2 | 33 | 0.00274755 | 0.43688119 |
| 86 | HSD3B2 | 33 | 0.00289557 | 0.39662921 |
| 87 | ANXA1 | 33 | 0.00520067 | 0.4525641 |
| 88 | DHCR24 | 32 | 0.00238868 | 0.43472906 |
| 89 | ACSL3 | 32 | 0.00240201 | 0.42174432 |
| 90 | CYP2C8 | 32 | 0.00208744 | 0.41529412 |
| 91 | MGLL | 32 | 0.00586825 | 0.44740177 |
| 92 | AGTR1 | 31 | 0.00301903 | 0.45198464 |
| 93 | CYP3A5 | 31 | 0.00252802 | 0.43101343 |
| 94 | NFKB1 | 30 | 0.00267135 | 0.43742255 |
| 95 | IDI1 | 30 | 0.0012722 | 0.41480611 |
| 96 | SOD1 | 30 | 0.00849554 | 0.46083551 |
| 97 | CCR5 | 30 | 0.00484165 | 0.43960149 |
| 98 | IL18 | 30 | 0.00885929 | 0.44291092 |
| 99 | CEBPA | 30 | 0.00166733 | 0.45314506 |
| 100 | VLDLR | 30 | 0.0013901 | 0.44069913 |
| 101 | ELOVL6 | 30 | 0.00743677 | 0.42124105 |
| 102 | NSDHL | 29 | 0.00116612 | 0.40856481 |
| 103 | CYP11A1 | 29 | 0.00352821 | 0.43634116 |
| 104 | ALOX5 | 29 | 0.00291429 | 0.42736077 |
| 105 | PLA2G4A | 29 | 0.00237261 | 0.41973841 |
| 106 | ELOVL5 | 29 | 0.00242505 | 0.41529412 |
| 107 | SCD5 | 29 | 0.00184853 | 0.42376951 |
| 108 | CD36 | 28 | 6.48E-04 | 0.43742255 |
| 109 | SLC27A2 | 28 | 0.00101595 | 0.41874259 |
| 110 | CYP4F2 | 28 | 0.00339299 | 0.44514502 |
| 111 | LIPG | 28 | 0.00278586 | 0.41627358 |
| 112 | STAR | 27 | 0.00429426 | 0.44402516 |
| 113 | ACAT2 | 27 | 0.00321628 | 0.39091916 |
| 114 | G6PD | 27 | 0.00542378 | 0.45489691 |
| 115 | PLA2G6 | 27 | 0.0109596 | 0.41142191 |
| 116 | FA2H | 27 | 0.01674437 | 0.40762125 |
| 117 | PTGS1 | 26 | 0.00193595 | 0.42891859 |
| 118 | SLCO1B1 | 26 | 0.00321628 | 0.43206854 |
| 119 | SYK | 26 | 0.00382344 | 0.42839806 |
| 120 | AKR1C3 | 25 | 0.00195257 | 0.40435281 |
| 121 | CNR1 | 25 | 0.00490191 | 0.44291092 |
| 122 | RBP4 | 25 | 0.00332975 | 0.43259804 |
| 123 | CES1 | 25 | 0.00667315 | 0.43472906 |
| 124 | PRKCA | 25 | 0.00470647 | 0.4263285 |
| 125 | FABP3 | 25 | 7.85E-04 | 0.43905473 |
| 126 | SLCO1A2 | 25 | 0.00917285 | 0.42073897 |
| 127 | CH25H | 25 | 0.00114794 | 0.41383353 |
| 128 | PLA2G2A | 25 | 0.00352154 | 0.43580247 |
| 129 | B2M | 24 | 0.0053958 | 0.41383353 |
| 130 | SHH | 24 | 0.00946798 | 0.44069913 |
| 131 | SMAD2 | 24 | 0.00216105 | 0.43366093 |
| 132 | ANGPTL3 | 24 | 0.00206125 | 0.40296804 |
| 133 | ORM1 | 24 | 0.00139664 | 0.41480611 |
| 134 | CYP2J2 | 24 | 0.00155288 | 0.38537118 |
| 135 | CFTR | 24 | 0.00309535 | 0.44125 |
| 136 | ACSL5 | 24 | 0.00540987 | 0.40113636 |
| 137 | ACSL4 | 24 | 0.0013953 | 0.41578327 |
| 138 | F5 | 23 | 0.00272843 | 0.41094296 |
| 139 | MVD | 23 | 3.61E-04 | 0.39222222 |
| 140 | CALR | 23 | 0.0027318 | 0.42944039 |
| 141 | APOC1 | 23 | 2.26E-04 | 0.42174432 |
| 142 | HSD17B12 | 23 | 0.00413846 | 0.39887006 |
| 143 | LMNA | 23 | 0.01753861 | 0.44910941 |
| 144 | RAC1 | 23 | 0.0110362 | 0.40296804 |
| 145 | CYP2D6 | 23 | 0.00337123 | 0.43154034 |
| 146 | CYP4F3 | 23 | 0.00115223 | 0.40389016 |
| 147 | LCN2 | 22 | 0.0024666 | 0.42124105 |
| 148 | VDR | 22 | 0.00136509 | 0.44627054 |
| 149 | CSF1 | 22 | 0.00119224 | 0.4253012 |
| 150 | MSR1 | 22 | 4.02E-04 | 0.41383353 |
| 151 | LBR | 21 | 0.00619173 | 0.38833883 |
| 152 | CYP1B1 | 21 | 8.99E-04 | 0.41238318 |
| 153 | SIGMAR1 | 21 | 7.60E-04 | 0.39265851 |
| 154 | PLA2G4C | 21 | 4.85E-04 | 0.39707537 |
| 155 | NLRP3 | 20 | 0.00233901 | 0.44514502 |
| 156 | LRP1 | 20 | 6.86E-04 | 0.43312883 |
| 157 | HSD17B1 | 20 | 5.43E-04 | 0.38621444 |
| 158 | DRD2 | 20 | 0.02297079 | 0.42478941 |
| 159 | HSPA1A | 20 | 0.00524231 | 0.43850932 |
| 160 | EBP | 20 | 8.48E-04 | 0.38079827 |
| 161 | ANXA2 | 20 | 0.00335318 | 0.4304878 |
| 162 | STS | 20 | 0.0014503 | 0.39397321 |
| 163 | ABCC1 | 20 | 0.00415122 | 0.41046512 |
| 164 | PLA2G3 | 20 | 1.18E-04 | 0.39485459 |
| 165 | NFYA | 19 | 8.22E-04 | 0.41480611 |
| 166 | LEPR | 19 | 8.28E-04 | 0.44291092 |
| 167 | CYP11B2 | 19 | 0.00105937 | 0.40113636 |
| 168 | PRKAA1 | 19 | 0.00599212 | 0.41973841 |
| 169 | ANGPTL4 | 19 | 0.00291821 | 0.41627358 |
| 170 | ADRB2 | 19 | 7.82E-04 | 0.42891859 |
| 171 | AKT2 | 19 | 0.0125943 | 0.42787879 |
| 172 | PRKACA | 19 | 0.0014378 | 0.42996346 |
| 173 | CEL | 19 | 0.00185663 | 0.43101343 |
| 174 | ALOX12B | 19 | 5.76E-04 | 0.37754011 |
| 175 | GC | 19 | 7.45E-04 | 0.41578327 |
| 176 | PLA2G5 | 19 | 1.16E-04 | 0.39222222 |
| 177 | APOD | 19 | 0.00676256 | 0.40113636 |
| 178 | APOF | 18 | 5.51E-05 | 0.39752252 |
| 179 | MBTPS1 | 18 | 0.00666265 | 0.42376951 |
| 180 | CYP2A6 | 18 | 0.00129404 | 0.39529675 |
| 181 | FABP2 | 18 | 1.92E-04 | 0.40809249 |
| 182 | FDX1 | 17 | 0.0014458 | 0.37394068 |
| 183 | NFKBIA | 17 | 0.00205766 | 0.41627358 |
| 184 | NPC1 | 17 | 0.00728803 | 0.42478941 |
| 185 | AKR1C1 | 17 | 5.67E-04 | 0.37713675 |
| 186 | LDLRAP1 | 17 | 4.53E-04 | 0.40668203 |
| 187 | RAC2 | 17 | 0.00547333 | 0.39529675 |
| 188 | CCL3 | 17 | 2.73E-04 | 0.41190198 |
| 189 | SHBG | 17 | 0.00137385 | 0.4263285 |
| 190 | SLC27A5 | 17 | 8.00E-04 | 0.40342857 |
| 191 | F8 | 16 | 0.00159679 | 0.4071511 |
| 192 | CYP11B1 | 16 | 4.90E-04 | 0.37002096 |
| 193 | INSIG2 | 16 | 1.78E-04 | 0.40574713 |
| 194 | MED15 | 16 | 5.33E-04 | 0.41046512 |
| 195 | ELOVL2 | 16 | 3.05E-04 | 0.3917869 |
| 196 | BDKRB2 | 16 | 0.00423548 | 0.37593184 |
| 197 | ACADVL | 16 | 6.54E-04 | 0.41874259 |
| 198 | SLC22A8 | 16 | 7.50E-04 | 0.35620585 |
| 199 | DPP4 | 16 | 8.91E-04 | 0.4253012 |
| 200 | NFYC | 15 | 3.63E-04 | 0.38663746 |
| 201 | TGFBR1 | 15 | 6.36E-04 | 0.40389016 |
| 202 | PRKAA2 | 15 | 4.97E-04 | 0.40903824 |
| 203 | FAAH | 15 | 0.00281069 | 0.43154034 |
| 204 | SYP | 15 | 0.00654123 | 0.4253012 |
| 205 | ELOVL3 | 15 | 1.13E-04 | 0.39309577 |
| 206 | PRKCB | 15 | 0.00116024 | 0.41578327 |
| 207 | MFGE8 | 15 | 2.27E-04 | 0.4071511 |
| 208 | SLCO2B1 | 15 | 6.10E-04 | 0.35584677 |
| 209 | LRP8 | 14 | 1.32E-04 | 0.39618406 |
| 210 | EPHX2 | 14 | 7.48E-04 | 0.40856481 |
| 211 | CAMP | 14 | 8.33E-04 | 0.40342857 |
| 212 | SULT2B1 | 14 | 3.32E-04 | 0.38162162 |
| 213 | HCK | 14 | 4.87E-04 | 0.40481651 |
| 214 | NR5A2 | 14 | 9.59E-04 | 0.39529675 |
| 215 | ABCB4 | 14 | 0.00274427 | 0.39752252 |
| 216 | DHRS11 | 14 | 2.06E-04 | 0.38327904 |
| 217 | CD209 | 14 | 3.23E-04 | 0.39887006 |
| 218 | SLC22A7 | 14 | 1.57E-04 | 0.35406219 |
| 219 | SLC47A1 | 14 | 1.26E-04 | 0.35692619 |
| 220 | CFB | 14 | 0.00180099 | 0.41334895 |
| 221 | NPC2 | 13 | 0.00138546 | 0.41046512 |
| 222 | FGF1 | 13 | 4.38E-04 | 0.40903824 |
| 223 | NCF2 | 13 | 5.50E-04 | 0.3870614 |
| 224 | CD24 | 13 | 3.74E-04 | 0.40621404 |
| 225 | XBP1 | 13 | 2.90E-04 | 0.4304878 |
| 226 | CD81 | 13 | 0.00498301 | 0.40903824 |
| 227 | CELSR2 | 13 | 7.85E-05 | 0.39222222 |
| 228 | F12 | 13 | 7.68E-04 | 0.39048673 |
| 229 | HLA-A | 12 | 0.00845599 | 0.36205128 |
| 230 | FDXR | 12 | 0.00163785 | 0.37275607 |
| 231 | MYLIP | 12 | 5.84E-05 | 0.40342857 |
| 232 | PTGDS | 12 | 0.00283454 | 0.42581423 |
| 233 | TGFBR2 | 11 | 3.66E-04 | 0.38579235 |
| 234 | PIK3CG | 11 | 0.00124145 | 0.38079827 |
| 235 | F13A1 | 11 | 9.13E-05 | 0.39309577 |
| 236 | RBP1 | 11 | 6.19E-05 | 0.38663746 |
| 237 | PPARD | 11 | 2.80E-04 | 0.39932127 |
| 238 | FAS | 11 | 2.20E-04 | 0.41142191 |
| 239 | TSPO | 11 | 3.90E-04 | 0.40022676 |
| 240 | SLC22A6 | 11 | 6.19E-05 | 0.33877159 |
| 241 | MIF | 11 | 1.68E-04 | 0.40574713 |
| 242 | FANCG | 10 | 0.00510014 | 0.34881423 |
| 243 | FANCC | 10 | 0.00378408 | 0.33942308 |
| 244 | HES1 | 10 | 0.00751431 | 0.35692619 |
| 245 | ATP5A1 | 10 | 8.77E-04 | 0.37956989 |
| 246 | AKR1D1 | 10 | 2.88E-04 | 0.36168033 |
| 247 | DRD3 | 10 | 6.94E-05 | 0.37997847 |
| 248 | DRD4 | 10 | 6.94E-05 | 0.37997847 |
| 249 | BARD1 | 10 | 0.00852069 | 0.35947047 |
| 250 | CYP2A13 | 10 | 1.45E-04 | 0.36168033 |
| 251 | NRP1 | 10 | 1.65E-04 | 0.3870614 |
| 252 | SLC22A11 | 10 | 6.83E-05 | 0.33812261 |
| 253 | SLC47A2 | 10 | 1.55E-05 | 0.33619048 |
| 254 | APITD1 | 9 | 0.00176364 | 0.31046614 |
| 255 | FANCF | 9 | 0.00271337 | 0.33207902 |
| 256 | ATP5B | 9 | 5.99E-04 | 0.38919515 |
| 257 | FANCD2 | 9 | 4.52E-04 | 0.29392173 |
| 258 | PDZK1 | 9 | 7.25E-04 | 0.39485459 |
| 259 | GPR132 | 9 | 3.75E-05 | 0.38579235 |
| 260 | S100A9 | 9 | 4.50E-04 | 0.38537118 |
| 261 | PTCH1 | 8 | 3.36E-04 | 0.362423 |
| 262 | PYCARD | 8 | 0.0057086 | 0.35801217 |
| 263 | LRP6 | 8 | 2.34E-04 | 0.38286334 |
| 264 | ABHD6 | 8 | 0.00162486 | 0.36391753 |
| 265 | PHLPP2 | 8 | 2.68E-04 | 0.36279548 |
| 266 | NCEH1 | 8 | 3.48E-04 | 0.36391753 |
| 267 | ANPEP | 8 | 1.04E-04 | 0.38079827 |
| 268 | CSNK2A1 | 8 | 1.79E-04 | 0.39485459 |
| 269 | FABP7 | 8 | 2.60E-04 | 0.39309577 |
| 270 | ERLIN2 | 8 | 5.06E-04 | 0.39222222 |
| 271 | LRP5 | 8 | 6.85E-04 | 0.39752252 |
| 272 | FADS3 | 8 | 1.27E-04 | 0.35873984 |
| 273 | ABCD3 | 8 | 9.42E-05 | 0.37002096 |
| 274 | SLCO1C1 | 8 | 3.03E-05 | 0.33844679 |
| 275 | SLCO4A1 | 8 | 4.84E-05 | 0.33651096 |
| 276 | C17orf70 | 7 | 0 | 0.28699187 |
| 277 | C19orf40 | 7 | 0 | 0.28699187 |
| 278 | SEC24C | 7 | 3.04E-04 | 0.35054618 |
| 279 | SEC24D | 7 | 3.04E-04 | 0.35054618 |
| 280 | FGFR4 | 7 | 1.32E-04 | 0.36963351 |
| 281 | KPNB1 | 7 | 2.05E-04 | 0.37834941 |
| 282 | FXN | 7 | 3.73E-04 | 0.35477387 |
| 283 | CAV3 | 7 | 1.30E-04 | 0.35406219 |
| 284 | ABHD12 | 7 | 5.26E-04 | 0.34040501 |
| 285 | SEC24B | 7 | 3.04E-04 | 0.35054618 |
| 286 | INHBA | 7 | 1.61E-04 | 0.34778325 |
| 287 | MT-CO1 | 7 | 2.13E-04 | 0.40205011 |
| 288 | CA9 | 7 | 7.08E-05 | 0.40250855 |
| 289 | ACSM3 | 7 | 2.45E-04 | 0.37956989 |
| 290 | DAG1 | 6 | 1.03E-04 | 0.34675835 |
| 291 | CYP4F12 | 6 | 1.06E-04 | 0.35019841 |
| 292 | C1S | 6 | 2.33E-05 | 0.38537118 |
| 293 | HIST1H3A | 6 | 6.95E-04 | 0.36656282 |
| 294 | MAPKAPK2 | 5 | 1.09E-05 | 0.37002096 |
| 295 | FABP9 | 5 | 2.48E-04 | 0.35159363 |
| 296 | CERS5 | 5 | 0.00583303 | 0.31322094 |
| 297 | ABCA12 | 5 | 2.54E-04 | 0.36963351 |
| 298 | NFE2L1 | 5 | 1.56E-06 | 0.37673426 |
| 299 | EHD1 | 4 | 0.00607374 | 0.31974638 |
| 300 | CHGA | 4 | 0.00128944 | 0.38162162 |
| 301 | SCUBE2 | 4 | 2.08E-04 | 0.33877159 |
| 302 | NR1D1 | 4 | 9.79E-05 | 0.34139265 |
| 303 | SGMS2 | 4 | 2.07E-04 | 0.3342803 |
| 304 | STX12 | 4 | 0.00218776 | 0.34778325 |
| 305 | C19orf80 | 3 | 0 | 0.31322094 |
| 306 | VPS4A | 3 | 0.00570536 | 0.31974638 |
| 307 | DISP1 | 3 | 1.20E-04 | 0.31887986 |
| 308 | HSPA1B | 3 | 0 | 0.34405458 |
| 309 | TMEM97 | 3 | 0 | 0.34846989 |
| 310 | RORA | 3 | 1.28E-04 | 0.35441767 |
| 311 | IFITM3 | 3 | 3.29E-04 | 0.29614094 |
| 312 | EPB41L3 | 3 | 0.01129925 | 0.23223684 |
| 313 | AHCTF1 | 3 | 5.00E-04 | 0.32837209 |
| 314 | FABP12 | 3 | 0 | 0.3374761 |
| 315 | CNTNAP2 | 3 | 0.01704445 | 0.30119454 |
| 316 | SGMS1 | 3 | 1.27E-04 | 0.33052434 |
| 317 | LPGAT1 | 3 | 1.07E-05 | 0.32208029 |
| 318 | C19orf12 | 3 | 0.00566572 | 0.31405694 |
| 319 | STX3 | 3 | 2.98E-04 | 0.31128748 |
| 320 | SEC14L2 | 3 | 7.58E-05 | 0.34950495 |
| 321 | ABHD2 | 3 | 4.64E-05 | 0.30222603 |
| 322 | SLC16A1 | 3 | 3.90E-05 | 0.32990654 |
| 323 | NLRX1 | 3 | 7.69E-04 | 0.31349911 |
| 324 | RNF168 | 2 | 0 | 0.26581325 |
| 325 | RBP5 | 2 | 2.58E-06 | 0.32385321 |
| 326 | FAM13A | 2 | 0 | 0.29465776 |
| 327 | FAM13B | 2 | 0 | 0.29465776 |
| 328 | EPB41L5 | 2 | 0 | 0.18866916 |
| 329 | CNTNAP1 | 2 | 0 | 0.18866916 |
| 330 | IP6K2 | 2 | 0 | 0.26701967 |
| 331 | PIR | 2 | 1.02E-04 | 0.31801802 |
| 332 | CYB5R1 | 2 | 0 | 0.29713805 |
| 333 | NLRC3 | 2 | 0 | 0.26402393 |
| 334 | RNF145 | 2 | 0 | 0.34405458 |
| 335 | PYGL | 2 | 0 | 0.33052434 |
| 336 | FAM63A | 2 | 0 | 0.36963351 |
| 337 | LCN6 | 2 | 1.07E-05 | 0.32867784 |
| 338 | CLIP3 | 2 | 0.00566572 | 0.30042553 |
| 339 | PMP2 | 2 | 6.02E-06 | 0.33942308 |
| 340 | ARV1 | 2 | 0 | 0.3342803 |
| 341 | GNPTAB | 2 | 5.71E-04 | 0.30042553 |
| 342 | PNPLA8 | 2 | 2.11E-05 | 0.34472656 |
| 343 | DDR1 | 2 | 1.04E-05 | 0.35620585 |
| 344 | EDIL3 | 2 | 0 | 0.35159363 |
| 345 | VPS4B | 1 | 0 | 0.24244505 |
| 346 | RAB11FIP2 | 1 | 0 | 0.24244505 |
| 347 | CA7 | 1 | 0 | 0.33619048 |
| 348 | FAM73A | 1 | 0 | 0.23915989 |
| 349 | TPRG1L | 1 | 0 | 0.29864636 |
| 350 | FAM20B | 1 | 0 | 0.29638959 |
| 351 | LCN15 | 1 | 0 | 0.28652597 |
| 352 | CLN8 | 1 | 0 | 0.23867478 |
| 353 | PLSCR3 | 1 | 0 | 0.332705 |
| 354 | FAM49A | 1 | 0 | 0.23117223 |
