# Supplementary material for: Network Pharmacology Identifies the Mechanisms of Sang-Xing-Zhi-Ke-Fang against Pharyngitis
Source: Evid Based Complement Alternat Med. 2020 Oct 12;2020:2421916. doi: 10.1155/2020/2421916 (PMC7576344; doi:10.1155/2020/2421916)
Supplement: Supplementary Materials — Table S1: 102 bioactive compounds obtained and screened out from TCMSP, BATMAN-TCM, and literature. Table S2: 886 targets of bioactive compounds collected using TCMSP and UniProt. Table S3: targets related to pharyngitis, including 5150 targets from CTD and 1803 targets from GeneCards with 695 targets duplicated. Table S4: 387 overlapping targets related to 19 bioactive compounds. Table S5: the results of topological features of the PPI network, including the values of topological features of 354 targets, while the other 33 targets were unrelated to each other target in the network. Table S6: the results of KEGG pathway enrichment, including 43 KEGG pathways were recognized as P < 0.05 with 28 pathways being recognized as P < 0.01. [file 2421916.f1.zip › Supplementary materials/Suppplementary Table S6.docx]

| Term | Number of pathway gene | P value |
| --- | --- | --- |
| hsa00591:Linoleic acid metabolism | CYP3A4,CYP2J2,CYP2C19,CYP2C9,CYP2C8,CYP2E1,CYP1A2,PLA2G4A,PLA2G1B,PLA2G2A,PLA2G6,PLA2G3, PLA2G4C, PLA2G5 | 1.20E-11 |
| hsa04370:VEGF signaling pathway | PIK3CG,PRKCA,PTGS2,MAPKAPK2,PRKCB,PLA2G4A,KRAS,RAC2,MAPK14,MAPK3,RAC1,PLA2G4C, AKT2 | 2.69E-06 |
| hsa04726:Serotonergic synapse | PRKCA,PLA2G4A,APP,CYP2J2,KRAS,CYP2C19,PTGS2, CYP2C9, CYP2C8, CYP2D6, MAPK3, PTGS1, ALOX12B, PRKACA, ALOX5, PLA2G4C, PRKCB | 4.54E-06 |
| hsa04664:Fc epsilon RI signaling pathway | IL4,PIK3CG,PRKCA,PRKCB,PLA2G4A,KRAS,RAC2,MAPK14,MAPK3,RAC1, PLA2G4C, SYK, AKT2 | 8.83E-06 |
| hsa00592:alpha-Linolenic acid metabolism | ACOX1,PLA2G4A,PLA2G2A,PLA2G1B,PLA2G6, PLA2G4C, PLA2G3, PLA2G5 | 3.08E-05 |
| hsa05212:Pancreatic cancer | PIK3CG, KRAS, RAC2, TGFBR1, MAPK3, TGFBR2, RAC1, NFKB1, SMAD2, EGF, TGFB1, AKT2 | 3.17E-05 |
| hsa05142:Chagas disease (American trypanosomiasis) | PIK3CG,CCL3,TGFBR1, TGFBR2, NFKBIA, NFKB1, SMAD2, BDKRB2, CALR, TGFB1, ACE, MAPK14, MAPK3, FAS, AKT2 | 3.98E-05 |
| hsa00980:Metabolism of xenobiotics by cytochrome P450 | CYP3A4, CYP2A13, CYP3A5, CYP1B1, CYP1A1, CYP2B6, CYP2C9, CYP2D6, CYP2A6, CYP2E1, CYP1A2, AKR1C1 | 1.09E-04 |
| hsa04071:Sphingolipid signaling pathway | PRKCA, PIK3CG, SGMS2, CERS5, NFKB1, SGMS1, BDKRB2, PRKCB, KRAS, RAC2, MAPK14, MAPK3, RAC1, ABCC1, AKT2 | 1.93E-04 |
| hsa05204:Chemical carcinogenesis | CYP3A4, CYP2A13, CYP3A5, CYP1B1, CYP1A1, CYP2C19, PTGS2, CYP2C9, CYP2C8, CYP2A6, CYP2E1, CYP1A2 | 2.22E-04 |
| hsa04014:Ras signaling pathway | PRKCA, PIK3CG, FGFR4, CSF1, NFKB1, PRKCB, PLA2G4A,KRAS, RAC2, RAC1, MAPK3,PLA2G1B, PLA2G2A, PLA2G6, PRKACA, FGF1, PLA2G3, PLA2G4C, EGF, PLA2G5, AKT2 | 4.17E-04 |
| hsa04380:Osteoclast differentiation | PIK3CG,NCF2,TGFBR1,CSF1,CREB1,PPARG,TGFBR2,NFKBIA,NFKB1,TGFB1,MAPK14,MAPK3, RAC1, AKT2, SYK | 4.83E-04 |
| hsa05210:Colorectal cancer | PIK3CG,KRAS,RAC2, TGFBR1, MAPK3, TGFBR2, RAC1, SMAD2, TGFB1, AKT2 | 5.51E-04 |
| hsa04068:FoxO signaling pathway | PIK3CG,TGFBR1,TGFBR2,SMAD2,SIRT1, TGFB1, G6PC, KRAS, MAPK14, MAPK3, PRKAA1, CAT, PRKAA2, EGF, AKT2 | 6.08E-04 |
| hsa00830:Retinol metabolism | CYP3A4, CYP3A5, DGAT1, CYP1A1, CYP2B6, CYP2C9, CYP2C8, HSD17B6, CYP2A6, CYP1A2 | 7.00E-04 |
| hsa04010:MAPK signaling pathway | PRKCA, FGFR4, TGFBR1, TGFBR2, NFKB1, HSPA1A, HSPA1B, MAPKAPK2, TGFB1, PRKCB, PLA2G4A, KRAS, RAC2, MAPK14, RAC1, MAPK3, PRKACA, FAS, FGF1, PLA2G4C, EGF, AKT2 | 7.04E-04 |
| hsa00982:Drug metabolism - cytochrome P450 | CYP3A4, CYP3A5, CYP2C19, CYP2B6, CYP2C9, CYP2C8, CYP2D6, CYP2A6, CYP2E1, CYP1A2 | 0.001095 |
| hsa04662:B cell receptor signaling pathway | PIK3CG, KRAS, RAC2, MAPK3, RAC1, CD81, NFKBIA, NFKB1, AKT2, SYK | 0.001218 |
| hsa05160:Hepatitis C | PIK3CG, PPARA, LDLR, RXRA, NFKBIA, NFKB1, KRAS, MAPK14, MAPK3, CD81, SCARB1, EGF, AKT2, NR1H3 | 0.001755 |
| hsa05164:Influenza A | PRKCA, PIK3CG, IL18, FDPS, NFKBIA, NLRX1, HSPA1A, NFKB1, HSPA1B, NLRP3, PRKCB, MAPK14, MAPK3, PYCARD, FAS, AKT2 | 0.002805 |
| hsa05200:Pathways in cancer | PPARD, PTGS2, PPARG, NFKBIA, NFKB1, BDKRB2, TGFB1, SHH, AGTR1, KRAS, RAC2, RAC1, PRKACA, FAS, FGF1, EGF, AKT2, PRKCA, CEBPA, PIK3CG, TGFBR1, RXRA, TGFBR2, SMAD2, PRKCB, MAPK3, PTCH1 | 0.004590903 |
| hsa04270:Vascular smooth muscle contraction | PRKCA, AGTR1, PLA2G4A, MAPK3, PLA2G2A, PLA2G1B, PLA2G6, PRKACA, PLA2G4C, PLA2G3, PLA2G5, PRKCB | 0.005242244 |
| hsa05231:Choline metabolism in cancer | PRKCA, PIK3CG, PLA2G4A, KRAS, RAC2, MAPK3, RAC1, EGF, PLA2G4C, PRKCB, AKT2 | 0.005313631 |
| hsa05223:Non-small cell lung cancer | PRKCA, PIK3CG, KRAS, RXRA, MAPK3, EGF, PRKCB, AKT2 | 0.005361934 |
| hsa05220:Chronic myeloid leukemia | PIK3CG, KRAS, TGFBR1, MAPK3, TGFBR2, NFKBIA, NFKB1, TGFB1, AKT2 | 0.006169641 |
| hsa00565:Ether lipid metabolism | PLA2G4A, PLA2G2A, PLA2G1B, PLA2G6, PLA2G4C, PLA2G3, PLA2G5 | 0.007113171 |
| hsa04668:TNF signaling pathway | PIK3CG, PTGS2, MAPK14, CSF1, CREB1, MAPK3, EDN1, NFKBIA, NFKB1, FAS, AKT2 | 0.007947413 |
| hsa04024:cAMP signaling pathway | PIK3CG, PPARA, ACOX1, DRD2, CREB1, NFKBIA, NFKB1, CFTR, ADRB2, RAC2, MAPK3, RAC1, PTCH1, PRKACA, LIPE, AKT2 | 0.009234847 |
| hsa05205:Proteoglycans in cancer | PRKCA, CAV3, PIK3CG, CAV1, ESR1, TGFB1, PRKCB, KRAS, CD44, MAPK14, MAPK3, RAC1, PRKACA, PTCH1, FAS, AKT2 | 0.010083223 |
| hsa00564:Glycerophospholipid metabolism | PLA2G4A, LPGAT1, LCAT, PLA2G2A, PLA2G1B, PLA2G6, PLA2G4C, PLA2G3, GPAM, PLA2G5 | 0.010609632 |
| hsa04750:Inflammatory mediator regulation of TRP channels | PRKCA, PIK3CG, PLA2G4A, CYP2J2, MAPK14, PLA2G6, PRKACA, BDKRB2, PLA2G4C, PRKCB | 0.012864872 |
| hsa04666:Fc gamma R-mediated phagocytosis | PRKCA, PIK3CG, RAC2, HCK, MAPK3, RAC1, PRKCB, AKT2, SYK | 0.015151434 |
| hsa04723:Retrograde endocannabinoid signaling | PRKCA, PTGS2, MAPK14, ABHD6, CNR1, FAAH, MAPK3, MGLL, PRKACA, PRKCB | 0.015458954 |
| hsa05221:Acute myeloid leukemia | PIK3CG, CEBPA, PPARD, KRAS, MAPK3, NFKB1, AKT2 | 0.019977771 |
| hsa04621:NOD-like receptor signaling pathway | IL18, MAPK14, MAPK3, PYCARD, NFKBIA, NFKB1, NLRP3 | 0.019977771 |
| hsa04150:mTOR signaling pathway | PRKCA, PIK3CG, MAPK3, PRKAA1, PRKAA2, PRKCB, AKT2 | 0.023376466 |
| hsa05161:Hepatitis B | PRKCA, PIK3CG, KRAS, TGFBR1, CREB1, MAPK3, NFKBIA, NFKB1, FAS, TGFB1, PRKCB, AKT2 | 0.023849236 |
| hsa04961:Endocrine and other factor-regulated calcium reabsorption | PRKCA, VDR, ESR1, PRKACA, BDKRB2, PRKCB | 0.028393557 |
| hsa04015:Rap1 signaling pathway | PRKCA, PIK3CG, FGFR4, DRD2, CSF1, PRKCB, KRAS, RAC2, MAPK14, CNR1, MAPK3, RAC1, EGF, FGF1, AKT2 | 0.031470906 |
| hsa04919:Thyroid hormone signaling pathway | PRKCA, PIK3CG, KRAS, RXRA, MAPK3, ESR1, SLCO1C1, PRKACA, PRKCB, AKT2 | 0.032820588 |
| hsa05166:HTLV-I infection | PIK3CG, TSPO, NRP1, TGFBR1, CREB1, TGFBR2, HLA-A, FDPS, NFKBIA, NFKB1, SMAD2, CALR, TGFB1, KRAS, XBP1, PRKACA, AKT2 | 0.035173777 |
| hsa04915:Estrogen signaling pathway | PIK3CG, KRAS, CREB1, MAPK3, ESR1, PRKACA, HSPA1A, HSPA1B, AKT2 | 0.036493436 |
| hsa05214:Glioma | PRKCA, PIK3CG, KRAS, MAPK3, EGF, PRKCB, AKT2 | 0.038257182 |
